# Nitrogen heterocycles form peptide nucleic acid precursors in complex prebiotic mixtures

**DOI:** 10.1038/s41598-019-45310-z

**Published:** 2019-06-26

**Authors:** Laura E. Rodriguez, Christopher H. House, Karen E. Smith, Melissa R. Roberts, Michael P. Callahan

**Affiliations:** 1 Department of Geosciences and Penn State Astrobiology Research Center, The Pennsylvania State University, 220 Deike Building, University Park, PA 16802 USA; 2 Department of Chemistry and Biochemistry, Boise State University, 312 Science Building, Boise, ID 83725 USA; 3 Astrochemistry Laboratory and Goddard Center for Astrobiology, National Aeronautics and Space Administration Goddard Space Flight Center, Greenbelt, MD 20771 USA

**Keywords:** Origin of life, Mass spectrometry, Astrobiology, Early solar system, RNA

## Abstract

The ability to store information is believed to have been crucial for the origin and evolution of life; however, little is known about the genetic polymers relevant to abiogenesis. Nitrogen heterocycles (N-heterocycles) are plausible components of such polymers as they may have been readily available on early Earth and are the means by which the extant genetic macromolecules RNA and DNA store information. Here, we report the reactivity of numerous N-heterocycles in highly complex mixtures, which were generated using a Miller-Urey spark discharge apparatus with either a reducing or neutral atmosphere, to investigate how N-heterocycles are modified under plausible prebiotic conditions. High throughput mass spectrometry was used to identify N-heterocycle adducts. Additionally, tandem mass spectrometry and nuclear magnetic resonance spectroscopy were used to elucidate reaction pathways for select reactions. Remarkably, we found that the majority of N-heterocycles, including the canonical nucleobases, gain short carbonyl side chains in our complex mixtures via a Strecker-like synthesis or Michael addition. These types of N-heterocycle adducts are subunits of the proposed RNA precursor, peptide nucleic acids (PNAs). The ease with which these carbonylated heterocycles form under both reducing and neutral atmospheres is suggestive that PNAs could be prebiotically feasible on early Earth.

## Introduction

The ability to store information was a critical step for the origin and evolution of life as we know it. Today life on Earth relies on DNA to store genetic information; however, the catalytic properties of RNA (among other evidence) has led to the widely-accepted hypothesis that early life relied solely on RNA as its primary genetic molecule^1^. Numerous studies have shown how the constituents of RNA (i.e. nucleobases, sugars, phosphate) may have formed *in situ* or were delivered to the early Earth^2–6^, and several studies have shown that ribonucleotides, the subunits of RNA, can be generated under prebiotic conditions^7–10^.

Alternatively, RNA was possibly preceded by a polymer that could spontaneously form from simpler precursors, aptly named a pre-RNA^11,12^. Proposed pre-RNAs have backbones different from that of RNA or utilize nitrogen heterocycles (N-heterocycles) other than the canonical nucleobases to store information. Of particular interest are those that are capable of spontaneously forming double helices with RNA via Watson-Crick base pairing as such supramolecular ordering provides a facile mechanism for the chemical evolution of pre-RNAs to RNA. Several such pre-RNAs have been studied including threose nucleic acid (TNA)^13^, glyoxylate-ribonucleic acid (gaRNA)^14^, glycol nucleic acid (GNA)^15^, and peptide nucleic acids (PNAs)^16^.

A further complication to elucidating the structures of plausible pre-RNA molecules stems from the diverse inventory of N-heterocycles from which they may have pooled (as abiotic reactions that generate the nucleobases also yield a variety of other N-heterocycles)^2,17,18^. In addition, a wide-range of N-heterocycles has been identified in carbonaceous meteorites, suggesting that delivery of extraterrestrial materials may have also served as a source for these molecules^4,19^. Unlike the canonical nucleobases, some of these alternative heterocycles (e.g. 2,4,6-triaminopyrimidine (TAP), melamine, and barbituric acid) can spontaneously form glycosides with ribose or other sugars and adopt Watson-Crick-like base pairs in aqueous solutions^20,21^. Given this, it has been proposed that the biological nucleobases are the result of evolutionary pressures and that the earliest genetic polymers incorporated alternative N-heterocycles^22^.

Considering the vast number of N-heterocycles and molecular backbones that could form a pre-RNA molecule, the number of plausible structures for genetic polymers relevant to the origin of life seem endless. Here, we investigated the chemistry of various N-heterocycles in complex mixtures (produced by spark discharge experiments) in order to simulate conditions approaching the chemical complexity expected for the prebiotic environment. We chose to focus on Miller-Urey mixtures as they generate a plethora of diverse organic compounds, including N-heterocycles, under conditions that may have been widespread on early Earth^3,17,23^. Moreover, many organics produced by spark discharges have been found in meteorites^23,24^ and are formed by various prebiotic reactions including HCN polymerization^2^, Fischer-Tropsch type synthesis^18^, and laboratory simulations of hydrothermal vent chemistry^25^. These observations imply that the canonical nucleobases and other N-heterocycles—whether they formed *in situ* or were delivered to primordial Earth—probably existed in complex mixtures like those generated by spark discharge experiments.

The reactivity of N-heterocycles in their chemical environment may not only have a profound effect on the heterocycle’s chemical properties (e.g. increasing solubility or facilitating the formation of more complex molecules), but also on whether they maintain the ability to base pair and store information. We show here that multiple prebiotically plausible pathways exist for the robust formation of carbonylated N-heterocycles in Miller-Urey mixtures and discuss how these structures could serve as precursors for the formation of PNAs on the early Earth.

## Method Rationale and Limitations

The primary goals of this study were to (1) elucidate the adducts that N-heterocycles commonly form in mixtures simulating the chemical complexity expected on early Earth and (2) evaluate plausible pre-RNA molecules compatible with these more complex structures. To this end, we studied the reactivity of 53 N-heterocycles representing five classes (pyridines, pyrimidines, triazines, purines, and pteridines) with complex mixtures produced under both reducing and neutral atmospheres to elucidate chemical trends, which resulted in hundreds of samples. Given the high number of samples and the complexity of each reaction mixture, it was not feasible to pursue product purification and structure elucidation via liquid chromatography-mass spectrometry, which precluded us from estimating product yields and determining which conformational isomer formed in any particular case. Instead we chose to analyze samples using high-resolution mass spectrometry (HRMS) with a direct analysis in real-time (DART) ion source to enable high sample throughput. The high mass resolution (>30,000 at *m/z* 400) and mass accuracy (typically <5 ppm) of the instrument permitted the assignment of molecular formulas, which enabled the recognition and identification of N-heterocycle adducts. DART was also selected for ionization because it readily protonates nitrogen-containing compounds, is less sensitive to matrix effects, does not usually produce salt adducts, and we found Penning ionization to be minimal, enabling us to rapidly analyze complex mixtures with minimal sample preparation^26^.

## Results and Discussion

### Reactivity trends in Miller-Urey mixtures

Miller-Urey spark discharge mixtures generate a plethora of organic compounds including aldehydes, nitriles, hydroxy acids, and amino acids^23,27–29^; these organics hold the potential to generate sugar, alcohol, electrophilic carbonyl, or nucleophilic side chains on N-heterocycles. The resulting side chain would impact the subsequent reactivity of the N-heterocycles and hence influence the types of molecular polymers they may form. For example, sugar and alcohol side chains can be readily phosphorylated^10^ or glyoxylated^14^ forming monomers akin to RNA or gaRNA; alternatively, hydroxymethylated N-heterocycles can spontaneously oligomerize^30^. Meanwhile, the formation of carboxylic acid and aldehyde side chains could serve as the precursor for PNAs should their carbonyls be attacked by amino acids. Lastly, nucleophilic side chains could facilitate the formation of organometallic clusters (e.g. with iron) as good nucleophiles often make decent ligands. To investigate whether N-heterocycles generate adducts that would favor the formation of a specific type of genetic polymer, we characterized the reactivity of N-heterocycles (Supplementary Table S1) in Miller-Urey spark discharge mixtures by searching for adducts with sugar, alcohol, carbonyl (i.e. electrophilic), and nucleophilic side chains (Supplementary Table S2).

**Figure 1:**
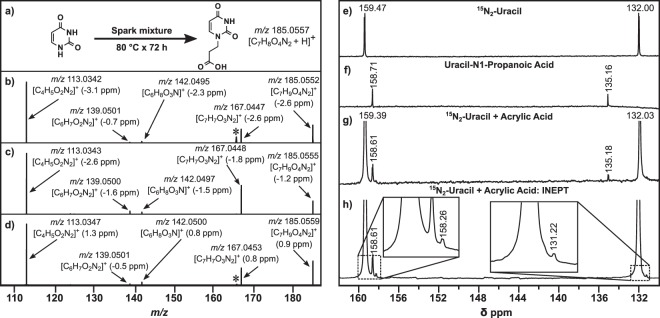
Elucidating the structure and formation of the major adduct formed in uracil-spark mixtures. Note that relative mass error given in ppm is calculated as 10^6^ × (*mass*_experimental_ − *mass*_theoretical_)/(*mass*_theoretical_). (**a**) depicts the adduct (*m/z* 185.0552) identified from DART-MS analysis of uracil incubated with a Miller-Urey spark discharge mixture generated under a reducing atmosphere (0.4 N_2_, 0.1 CO_2_, 0.25 CH_4_, 0.25 H_2_). (**b**,**c**) show the product ion spectra (MS/MS) of the precursor ion (*m/z* 185.05) at 40% collision energy (14 eV) isolated from uracil incubated with: (**b**) spark mixture and (**c**) acrylic acid. (**d**) shows the MS/MS of uracil-N1-propanoic acid standard. The * indicates an instrument artifact. (**e–h**) ^15^N-NMR results confirm that acrylic acid adds preferentially to the N1 position of uracil. (**e**) ^15^N_2_-Uracil produces two doublets corresponding to N1 (δ132.00) and N3 (δ159.47). (**f**) Uracil-N1-propanoic acid standard shows that addition at the N1 position shifts the N1 peak downfield (δ135.16) and N3 peak upfield (δ158.71). (**g**) The four peaks produced from ^15^N_2_-Uracil incubated with acrylic acid (100 °C x 3 h in D_6_-dimethyl sulfoxide (D_6_-DMSO)) correspond to uracil and uracil-N1-propanoic acid. (**h**) INEPT spectrum of the reaction mixture shows peaks only for nitrogens that have protons directly attached; the N1 peak (δ135.18) was not observed, confirming an addition at the N1 position. Note that the presence of two additional peaks (δ158.26 and δ131.22), suggests that the C5 adduct is a minor product in this reaction. The tops of the spectra have been truncated due to the peak height of uracil. All NMR spectra were obtained in D_6_-DMSO.

Figure 1(a–d) summarizes the strategy used to characterize select products observed in heterocycle-spark reaction mixtures using a uracil adduct (*m/z* 185.0556 = uracil-C_3_H_5_O_2_, Fig. 1a) that was formed under a reducing atmosphere (N_2_-CO_2_-CH_4_-H_2_) as an example. Tandem mass spectrometry (MS/MS) of the uracil adduct (Fig. 1b) shows a pattern consistent with a terminal carboxylic acid group (*m/z* 167.0447 = [M-H_2_O]^+^; *m/z* 139.0497 = [M-CH_2_O_2_]^+^). When uracil is incubated with acrylic acid in a separate reaction, we observed a product with identical mass (*m/z* = 185.0555) as the uracil-spark adduct. Comparison of the MS/MS spectra in Fig. 1(b,c) reveals that the fragmentation pattern of the uracil-spark and uracil-acrylic acid adducts match. The fragmentation pattern of a commercial reference standard of uracil-N1-propanoic acid (Fig. 1d) and ^15^N-NMR spectra (Fig. 1g,h) of uracil incubated with acrylic acid indicates that the reaction proceeded at the N1 position in the spark reaction mixture. Thus, the N-heterocycle adduct at *m/z* 185.0556 in the heterocycle-spark reaction mixture was identified as uracil-N1-propanoic acid.

Using these methods, we confirmed that specific organics in the spark discharge mixture react readily with a range of N-heterocycles (Supplementary Tables S3–S6; Supplementary Figs S3–S25); from this information we inferred that products having side chains with identical chemical compositions and thus the same Δ*m/z* (i.e. the difference in mass between the N-heterocycle adduct and parent N-heterocycle) were generated via the same mechanisms.

#### Reducing atmosphere

N-heterocycles were incubated with spark discharge mixtures generated under a strongly reducing atmosphere as they were originally perceived to be more conducive for organic synthesis than neutral gases^31^. Although, Earth’s early atmosphere was likely near neutral (N_2_, CO_2_) with trace amounts of H_2_, CO, and H_2_S^32^, volcanic outgassing and bolide impacts could have generated large, but transient amounts of reduced gases^33^.

**Figure 2:**
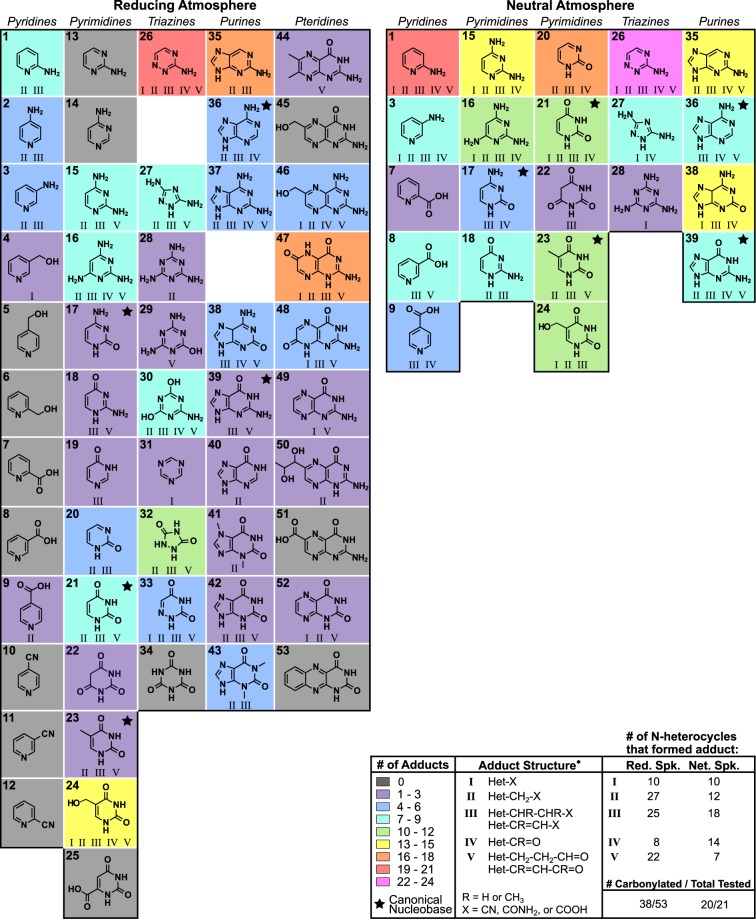
The reactivity of N-heterocycles when incubated with a Miller-Urey spark discharge mixture formed under a reducing (i.e. Red. Spk.) or neutral (i.e. Net. Spk.) atmosphere. Heterocycles are annotated by both their reactivity (background colors) and by which of the most common carbonyls they form (listed as I-V in the legend). Products of hydrolysis (e.g. CN/CONH_2_/COOH) were counted as a single adduct. *Structures I-V are assignments based on accurate mass measurements (typically <5 ppm error). Note [**22**, **25**, **31**, and **51**] were not ionized by the DART source and may be more reactive than shown. For compound names and a complete list of corresponding adducts, see Supplementary Tables S3 and S4.

Figure 2 summarizes the reactivity of 53 N-heterocycles in spark discharge mixtures produced from a 1 bar atmosphere containing 0.4 N_2_, 0.1 CO_2_, 0.25 H_2_, and 0.25 CH_4_ (i.e. reducing spark mixture) over a 0.2 M phosphate buffer solution (adduct details listed in Supplementary Table S3). Markedly, with the exception of 4-pyridinecarboxylic acid [**9**], N-heterocycles with cyano or carboxylic acid groups did not produce detectable adducts. Conversely, the majority of N-heterocycles without these electron withdrawing groups (EWGs), including the canonical nucleobases, formed at least one adduct containing a carbonyl carbon. These carbonylated heterocycles were 1–3 carbons in length, sometimes methylated (at either the α or β-carbon on the side chain), and contained a terminal aldehyde, nitrile, amide, or carboxylic acid functional group.

#### Neutral atmosphere

Given that Earth’s early atmosphere was likely near neutral based on current models^32^, we wanted to see whether N-heterocycles would carbonylate from a mixture generated under a N_2_-CO_2_ atmosphere lacking any reduced gases. Previous work has shown that neutral gas mixtures (1:1 N_2_:CO_2_) over an aqueous buffer form amino acids in higher yields^34^ than previously thought^28^, albeit at levels lower than that of reducing atmospheres. It is predicted that neutral atmospheres are not as conducive for amino acid synthesis partially because they produce less HCN and NH_3_^34^, which incidentally are likely precursors of the reactants that carbonylated N-heterocycles in our reducing spark mixture. Moreover, neutral atmospheres generate higher levels of nitrates and nitrites, the latter of which can go on to form nitrosamines from amino acids^34^ and N-heterocycles^35^.

Twenty-one N-heterocycles were incubated with a spark discharge mixture formed under a 1 bar atmosphere of 0.5 N_2_ and 0.5 CO_2_ gases (i.e. neutral spark mixture). Heterocycles were selected because of either their reactivity in the reducing spark mixture (Fig. 2 Reducing Atmosphere) or their relevance to previous pre-RNA studies^20,21,36–38^ (see Supplementary Table S1). In the majority of cases, the number of adducts formed were the same or higher compared to those using spark reaction mixtures from a reducing atmosphere. In addition, we identified at least one carbonylated adduct for almost every N-heterocycle tested (Fig. 2 Neutral Atmosphere), with many forming the same products as when they were incubated with the reducing spark mixture (see Supplementary Table S4 for a complete list of adducts). It should be noted that in addition to forming carbonylated adducts, several N-heterocycles (including the canonical purines) formed products with masses corresponding to various sugar-like side chains, including 4-, 5-, and 6-carbon sugars or sugar alcohols (see Supplementary Tables S4 and S6). However, these adducts were uncommon (i.e. each only formed by 2–5 N-heterocycles) and additional work is needed to verify the identity of the side chains.

Given that almost all the N-heterocycles with carboxylic acid groups were unreactive under a reducing atmosphere, we were intrigued to find that all three pyridine carboxylic acids tested were reactive [**7**, **8**, **9**], with [**8** and **9**] forming carbonylated heterocycles; 2-pyridinecarboxylic acid [**7**] formed a single adduct containing an alcohol side chain. One possible explanation for the reactivity of these compounds in the neutral but not reducing spark mixture may be that the former produces considerably fewer nucleophiles like HCN and ammonia^39^ that would otherwise sequester the carbonylating reactants^23,40^. This suggests that heterocycles with carboxylic acids can act as a nucleophile to form carbonylated heterocycles under certain favorable reaction conditions; indeed, previous studies have shown that such heterocycles undergo carbonylation in isolated reactions^41,42^.

#### Reactivity trends of N-heterocycles

We surmised that most of the N-heterocycle adducts were formed by the N-heterocycles attacking electrophiles; thus, those that were unreactive (and ionized by DART) can be considered poor nucleophiles. To some extent pK_a_ can be used to predict whether heterocycles will be unreactive as those having an exceptionally low pK_a_ are strong acids, but very poor nucleophiles. Being strong acids, these heterocycles hold on to their electrons tightly, hindering their interaction with electrophiles. This value is especially important for heterocycles whose conjugate base is neutral (e.g. pyridine as the conjugate base for pyridinium cations) and thus significantly less nucleophilic than conjugate bases that are negatively charged (e.g. deprotonated uracil). In accordance with this pattern the reported pK_a_ values of the conjugate acids (i.e. pK_aH_) for 2-, 3-, and 4-cyanopyridines (all of which were unreactive in the reducing spark mixture) are very low (−0.26, 1.36, and 1.90, respectively). The pK_aH_ of the ring nitrogen in the cyanopyridines reflects how the cyano group pulls electrons from the ring nitrogen via resonance effects, decreasing its nucleophilicity and deactivating the molecule towards electrophilic reactions. In addition to decreasing the electron density surrounding the ring nitrogen of pyridine, cyano groups also decrease the nucleophilicity of the ring N via steric hindrance as the bulky cyano group partially blocks the ring N from electrophilic attack.

Although carboxylic acids (R-COOH) are EWGs, once they deprotonate and form carboxylates (R-COO^−^) they are only slightly deactivating. In our reaction mixtures (pH 8), all of our carboxylic acid heterocycles exist as carboxylates. In consequence, the pK_aH_ of the ring nitrogen is only slightly less than pyridine (~5 for pyridine carboxylic acids). Despite what their pK_aH_ indicates, the pyridine carboxylic acids remain relatively weak nucleophiles; therefore, their low reactivity is probably due to steric hindrance from the bulky side chain near the ring nitrogen. This would also explain why 4-pyridinecarboxylic acid [**9**] is the only reactive pyridine carboxylic acid in the reducing spark mixture and why 2-pyridinecarboxylic acid [**7**] is the least reactive of the three in the neutral spark mixture (Supplementary Table S4). This trend is also consistent with 4- and 2-hydroxymethylpyridine [**5**, **6**] being unreactive in the reducing spark mixture despite the ring nitrogen being a decent base (pK_aH_ ~9); 3-hydroxymethylpyridine [**4**] is slightly reactive, forming two detectable adducts, only one of which is from electrophilic attack. In comparison, 4-aminopyridine [**2**], which has a similar pK_aH_ (9.2) to 4-hydroxymethylpyridine [**5**] (pK_aH_ 8.9) but is not sterically hindered, generated 6 detectable adducts while [**5**] was unreactive.

#### Robust Reactions of N-Heterocycles in Miller-Urey Mixtures

**Figure 3:**
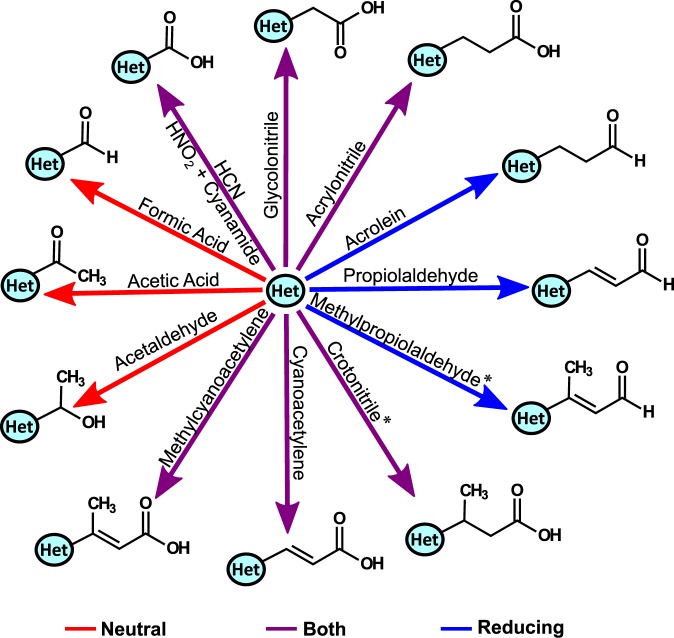
The formation of major adducts identified from N-heterocycles (labeled Het) incubated with Miller-Urey spark discharge mixtures. Major reactions were identified by grouping adducts based on chain length, terminal functional group, and whether the chain was saturated (see Supplementary Table S6 for details). This grouping revealed that the majority contained alkyl and acrylic side chains 1–3 carbons in length with terminal aldehyde or CN/CONH_2_/COOH groups. Of these groups, only the individual adducts that that were formed by at least 10 N-heterocycles were deemed major and included in this figure. Blue, red, and purple arrows indicate the reaction was robust when N-heterocycles were incubated with mixtures formed under a reducing atmosphere, neutral atmosphere, and both atmospheres, respectively. For clarity, reactants are shown as their nitrile precursors and adducts as their final hydrolysis product (carboxylic acids and aldehydes). *Indicates that it is equally plausible that a structural isomer of the reactant (methacrylonitrile and 3-butyn-2-one for crotonitrile and methylpropiolaldehyde, respectively) attacked the N-heterocycle, forming a structural isomer of the structure shown (see Supplementary Table S2 for the possible structures).

We investigated whether N-heterocycles gain sugar, alcohol, carbonyl, or nucleophilic side chains in prebiotically plausible complex mixtures. Our results show that in both reducing and neutral spark mixtures the majority of adducts contained carbonyl side chains (see Supplementary Table S6); in comparison, the formation of nucleophilic, sugar, and alcohol side chains (with the exception of acetaldehyde and cyanamide adducts in the neutral spark mixture) were exceptionally low (see Supplementary Table S6). Figure 2 summarizes the most common reactions of N-heterocycles observed in spark discharge mixtures (only the hydrolysis product of each adduct (i.e. acids) will be referred to hereafter). We assigned reactants based on their availability in spark mixtures (i.e. compounds that have been reported in the literature), demonstrated reactivity with N-heterocycles based on isolated reactions resulting in the same N-heterocycle adduct *m/z* (Table 1, Supplementary Table S5), and (for adducts with sufficient abundance) MS/MS spectra (Supplementary Figs S7–S25); in select cases we also compared the MS/MS of the spark adduct with that from an isolated reaction—in every case the fragmentation pattern matched. Notably, the only reactions that were major in both reducing and neutral spark mixtures were those that generated carboxylic acid side chains (Fig. 3).

**Table 1:** Organics predicted to have reacted with N-heterocycles incubated in spark discharge mixtures.

*Predicted Reactant* Structure	Literature that identifies reactant in spark discharge mixtures	Tested N-heterocycles (this study) *See* Supplementary Table S5 *for details*	Relevant Studies from the Literature
**N-heterocycles (listed as bold numbers) that form target adduct from predicted reactant in isolated reactions:**			
*HCN* or *Nitrous Acid* + *Cyanamide* HNO_2_ + NH_2_-CN	*HCN*: (31, 34, 39) *HNO*_2_: (34) *Cyanamide*: Urea (23, 39)^*^ (Cyanamide + H_2_O)	*NaCN*: #**24**, **26**	*NaCN*: #**24** (74) *HCN*: #**48** (74) *Nitrous Acid*: cytidine, adenosine, xanthosine and **39**, **42** (35)
*Glycolonitrile* HO-CH_2_-CN	(31, 39)	#**1**, **3**, **16**, **21**, **24**, **26**, **27** **36**	*Formaldehyde + HCN*: #**21** (74)
*Acrylonitrile/amide/acid* H_2_C = CH-CN	β-alanine (23, 24, 34, 76)^*^ (NH_3_ + Acrylonitrile)	#**1**, **3**, **17**, **18**, **21**, **23**, **24**, **36**	#**21** and **36** (42), #**23** and **39** (43)
*Crotonitrile/amide/acid* H_3_C-CH = CH-CN or *Methacrylonitrile/amide/acid* ^‡^ H_2_C = C(CH_3_)-CH-CN	β-aminobutyric acid (24)^*^ (NH_3_ + Crotonitrile) β-aminoisobutyric acid (24)^*^ (NH_3_ + Methacrylonitrile)	*Crotonitrile*: #**1**, **20**, **21**, **27**, **36**	^¶^*Crotonaldehyde*: #**23** (42) *Ethylcrotonate*: #**21**, **23**, **36**, **39** (43)
*Cyanoacetylene* or *Propiolamide/ Propiolic Acid* H-C≡C-CN	(40)	^§^ *Propiol Amide/Acid*: #**1**, **3**, **15**, **17**, **18**, **21**, **23**, **24**, **35**, **36**	*Cyanoacetylene*: #**23** (77), Cytidine monophosphate and Adenosine monophosphate (78), Urea and Ammonia (40)
*Methylcyanoacetylene* H_3_C-C≡C-CN	(Methyl acetylene (•C_2_-CH_3_) + HCN) (79)^†^	^§^ *2-butynoic acid*: #**1**, **16** ^§^ See crotonitrile	
*Acrolein* H_2_C = CH-CHO	(47)	—	#**15**, **17**, **18**, **21**, **22**, **23**, **24**, **36**, **37**, **38**, **39**, **40**, **41** (42)
*Propiolaldehyde* H-C≡CH-CHO	(46, 47) Mechanism: •C_2_H + CH_2_O (80) or:C = C = C: + 2 H_2_O (46)	#** 21**, **36** ^§^ See propiolamide/acid	# **17**, **21**, **23**, **36**, **39** (77)
*2-methylpropiolaldehyde* H_3_C-C≡C-CHO or *3-Butyn-2-one* ^‡^ H-C≡C-CO-CH_3_	H_3_C-C_2_• + CH_2_O (80)^†^:C = C = C: + CH_4_ + 2 H_2_O (46, 81, 82)^†^	^§^ See propiolaldehyde and methylcyanoacetylene	*3-butyn-2-one:* #**23** (77), adenosine (83), various anilines (84)
*Acetaldehyde* H_3_C-CO-H	Alanine (34)^*^ (NH_3_ + HCN + Acetaldehyde)	—	# **21** (74)
*Acetic Acid* H_3_C-COOH	(23, 76)	—	*Ammonium Acetate* + *Acetic Acid*: anilines (85)
*Formic Acid* H-COOH	(23, 76)	#**16**, **21**, **27**, **36**	Ethylenediamine and aromatic amines (86), aminopyrimidines (7)

References 7, 23, 31, 34, 35, 39, 40, 42, 43, 46, 47, 74–86 are listed in parenthesis. N-heterocycles are listed as bold numbers following # (refer to Fig. 2 and Supplementary Table S1 for structures and names, respectively). ^*^ or ^†^Indicates that the reactant was not directly detected, but that its formation in spark mixtures is inferred based on the identification of: ^*^The product of the predicted reactant with other spark compounds (mechanism shown in parenthesis) or ^†^Compounds that combine to form the predicted reactant. ^‡^Methacrylonitrile/amide/acid and 3-butn-2-one are equally plausible for generating the observed methylated carbonyl side chain as crotonitrile/amide/acid and 2-methylpropiolaldehyde, respectively. Note that Wolman and colleagues^24^ previously detected β-aminobutyric acid (from crotonitrile) and β-aminoisobutyric acid (from methacrylonitrile) in approximately equal amounts in spark discharge mixtures generated under a reducing atmosphere (CH_4_, N_2_, H_2_O, with trace NH_3_). Although the reactivity of methacrylonitrile and 3-butyn-2-one was not investigated, they are likely more reactive than their respective isomers, crotonitrile and 2-methylpropiolaldehyde, as they are methylated at the α-carbon and are thus not sterically hindered at the β-carbon which is the site of nucleophilic attack during the Michael addition.

^**§**^The reactivity of the predicted reactant can be inferred from that of a similar organic (e.g. those with different terminal functional groups) or a double instead of triple bond. It has been shown that the most reactive Michael acceptors are those with aldehydes (vs nitriles/esters/acids)^42,77^ and triple bonds (e.g. cyanoacetylene) rather than double (e.g. acrylonitrile)^78^. See Supplementary text, section 2.4 for details.

^¶^Organics with a methyl group attached to the β-carbon of an acrylic compound; the reactivity of these organics with N-heterocycles suggests that the methyl group of crotonitrile would not inhibit its ability to behave as a Michael acceptor in a reaction with N-heterocycles.

#### HCN/Cyanamide adducts

**Figure 4:**
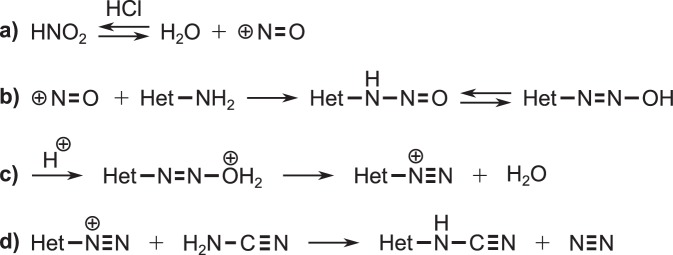
Proposed formation mechanism of cyanamide adducts from N-heterocycles with exocyclic amine groups that were incubated with a neutral spark reaction mixture. (**a**) Nitrous acid forms nitrosonium ions. (**b**) The amine groups of N-heterocycles undergo nitrosation and eventually form (**c**) diazonium cations that undergo (**d**) nucleophilic substitution with cyanamide (H_2_N-CN) or the cyanamide derivative, urea (H_2_N-CONH_2_).

In the aqueous buffer (0.2 M phosphate buffer, pH 8), about 10% of HCN is deprotonated (pKa 9.2); the resulting anion is an excellent nucleophile in reactions with N-heterocycles whose rings are sufficiently electron deficient. Accordingly, we found that the majority of cyanide adducts in reducing atmospheres were formed by pteridines and triazines that possessed a strongly electrophilic carbon susceptible to nucleophilic attack (e.g. [**26**], [**31**], and [**33**]). Thus, it was surprising to find that in the neutral spark mixture, pyrimidines and triazines containing two or more electron donating amine groups (e.g. melamine [**28**] and TAP [**16**]) formed cyano adducts, especially considering that Cleaves and coworkers^34^ measured significantly less HCN in spark discharge mixtures generated under an identical atmospheric ratio (1:1 N_2_:CO_2_). Given this, we propose that these cyano-adducts are formed via an intermediate that was present in the neutral but not reducing spark mixture. One such possibility is nitrosamines via nitrous acid generated from the spark discharge through a N_2_-CO_2_ atmosphere^34^. In water nitrous acid readily forms nitrosonium ions (Fig. 4a) that then react with nucleophilic amine groups forming nitrosamines (Fig. 4b). For aromatic compounds like the N-heterocycles, loss of water from the nitrosamine gives aryl diazonium cations (Fig. 4c)—excellent leaving groups that lower the activation energy for nucleophilic attack. Subsequent attack of the aryl diazonium cations by nucleophilic cyanamide would generate an adduct with identical mass to that expected from the parent heterocycle undergoing HCN nucleophilic substitution (Fig. 4d).

#### Glycolonitrile adducts

Intriguingly, N-heterocycles with an acetic acid group, our most consistently observed adduct, are the “nucleoside” subunit of the PNA shown to readily base pair with RNA^16^ (i.e. PNAs whose heterocycles are linked to a N-(2-aminoethyl)glycine (AEG) backbone by an acetamide bridge: Het-CH_2_-CO-AEG), hereafter referred to as aegPNA. These carbonylated heterocycles formed via a Strecker-like synthesis on the N-heterocycle with glycolonitrile (or alternatively, formaldehyde, followed by HCN). Notably, when we tested the reactivity of glycolamide with guanazole we did not observe the corresponding acetic amide nor acetic acid adduct (a detailed discussion of the reactivity of glycolonitrile with guanazole can be found in the Supplementary text, section 2.2 and Supplementary Fig. S5). These results coincide with previous observations that glycolonitrile—and by extension, its precursors, HCN and formaldehyde—are abundant products of spark discharges under reducing atmospheres^39^. Our results also show that N-heterocycles form the heterocycle-acetic acid molecules even in mixtures produced under neutral atmospheres where the concentrations of HCN are suspected to be relatively low^31,34^. While concentrations of HCN decrease under increasingly neutral atmospheres, the proportional amounts of formaldehyde and glycolonitrile may increase. Accordingly, it was found that glycolonitrile yields were highest under a N_2_-H_2_-CO_2_ atmosphere (compared to the same atmosphere but with CO or CH_4_ instead of CO_2_); notably the highest yields were found when the H_2_:CO_2_ ratio was 3 (compared to 0.5)^39^.

#### Michael adducts

The majority of the reactions depicted in Fig. 3 are Michael additions of N-heterocycles with 3-carbon-long α,β-conjugated compounds with a terminal carbonyl group (derived from methylcyanoacetylene, cyanoacetylene, crotonitrile, methylpropiolaldehyde, propiolaldehyde, acrolein, and acrylonitrile). Previous work has repeatedly shown that these types of reactions are regioselective with carbonylation being thermodynamically favored at the N1 and N9 positions of pyrimidines and purines, respectively^42–45^—the sites where ribose attaches to the canonical nucleobases in RNA. In accordance with this, we found that the Michael acceptors propiolic acid and acrylic acid preferentially add to the N1 position of uracil (Fig. 1; Supplementary Fig. S6).

#### Reactions unique to either reducing or neutral atmospheres

We identified several reactions that were common in either the reducing or the neutral spark mixtures, but not in both. The addition of the Michael acceptors acrolein, propiolaldehyde, and methylpropiolaldehyde were frequently observed only in reactions with a reducing atmosphere, which concurs with the fact that all three are easily generated via methane^46,47^ (Table 1). However, it should be noted that regarding the methylpropiolaldehyde and acrolein adducts, this observation may be misleading as the majority of the N-heterocycles that formed these adducts in the reducing spark mixture were not studied in the neutral spark mixture.

On the other hand, a greater presence of acetaldehyde, acetic acid, and formic acid adducts under a N_2_-CO_2_ atmosphere are consistent with measurements of excess formaldehyde and proportionally less HCN in neutral spark mixtures^39^. Since HCN sequesters aldehydes, low HCN yields correspond to higher acetaldehyde concentrations^48^ (for a discussion of the lack of observed formaldehyde adducts see Supplementary text, section 2.1). The paucity of free HCN may have been due to the efficient formation of HCN products (such as glycolonitrile) by cyanohydrin reactions as deduced by our results here and as seen in prior work^34^; alternatively, hydrolysis of HCN to formic acid could have been exacerbated by nitric and nitrous acids present in the neutral spark mixture^34^. Presumably, relatively high formic acid-to-HCN (and in parallel acetic acid-to-acetonitrile) ratios accounts for the robust production of formyl and acetyl adducts in the neutral mixtures.

### Discussion of Other Prebiotic Reactions Relevant to Our Results

Remarkably, the majority of the organics that carbonylated N-heterocycles were Michael acceptors that can also act as N-heterocycle precursors (e.g. urea + cyanoacetylene, propiolic acid, or acrylonitrile → cytosine/uracil)^46,49–51^. In addition, it has been shown that carbonylated nucleobases can be generated via purine and pyrimidine precursors that reacted with the carbonylating organics (e.g. hydantoic acid (from urea + glycolonitrile + 2 H_2_O) + cyanoacetaldehyde → cytosine/uracil-acetic acid)^52^. Overall, these previous studies, together with our results, suggest that there are multiple chemical pathways by which carbonylated heterocycles may have formed on early Earth. For an in-depth discussion of trends of nucleobase carbonylation and the possibility for a one-pot synthesis of carbonylated nucleobases in Miller-Urey-type reaction mixtures (based on results from previous publications and our observations of the spark mixtures generated for this study) see Supplementary text, sections 2.4 and 2.5; see Table S7 for yields of carbonylated nucleobases in isolated reactions obtained by previous studies.

Under both reducing and neutral atmospheres, the most common adducts were those containing carbonyl side chains 1- to 3-carbons long. The carbonyl carbon of these side chains, being electron deficient, is susceptible to nucleophilic attack by electron-rich molecules (e.g. amino acids)—the exception being the carbonyl resulting from nitrosation and cyanamide (see Supplementary text, section 2.3). Previous work has shown that amino acids readily react with N-heterocycles containing aldehyde side chains in aqueous solution, forming nucleobase-peptide molecules connected via an imine bond^41^. The formation of amide bonds may be facilitated by subjecting solutions containing N-heterocycle adducts, amino acids, and hydroxy acids to wet-dry cycles, as the latter compound promotes peptide bond formation^53^. Intriguingly, both amino acids and hydroxy acids are generated in relatively high yields from Miller-Urey reactions^23,27^. In fact, AEG, the backbone of aegPNA, has been identified in spark discharges of CH_4_, N_2_, NH_3_, and H_2_O atmospheres^52^, albeit in low yields and in the presence of numerous other amino acids^23^. Given this, a diverse set of PNA monomers containing AEG and various other amino acids is likely to be generated from carbonylated heterocycles within complex mixtures.

The resulting PNA monomers with aldehyde, acrylic, and carboxylic acid side chains could subsequently polymerize via Knoevenagel condensations^41^, free radical-induced polymerization^54^, or continuous wet-dry cycles (akin to amino acid polymerization^53^), respectively. As a diverse set of PNA monomers would be available during polymerization, the resulting macromolecule would likely be composed of a hetero-peptide backbone rather than a uniform backbone like aegPNA. Alternatively, carbonylated heterocycles can be attacked by an existing nucleophilic backbone such as poly-AEG^55^ or HCN polymer. Moreover, the carbonyl side chains, being hydrophilic, help solubilize N-heterocycles which in turn may facilitate the supramolecular assembly of carbonylated heterocycles and peptide-nucleobase monomers that base pair in solution^56^. The diversity of these polymerization mechanisms suggests that a facile transition of carbonylated heterocycles to complex polymers is prebiotically plausible under a broad range of conditions.

Our results suggest that formylation of N-heterocycles, including the aminopyrimidines studied here, is more favorable under a neutral atmosphere. Intriguingly, formylated 5,6-diaminopyrimidines can become N9 purine nucleosides in formose reaction mixtures subjected to drying^7^. As neutral atmospheres generate more free aldehydes than reducing atmospheres^39^, they may also be more conducive than reducing atmospheres for sugar synthesis and hence the conversion of formylated 5,6-diaminopyrmidines to purine glycosides. Therefore, formylation of N-heterocycles under neutral atmospheres could serve as a first-step towards PNA monomers as well as TNA and RNA purine nucleosides.

## Conclusions

**Figure 5:**
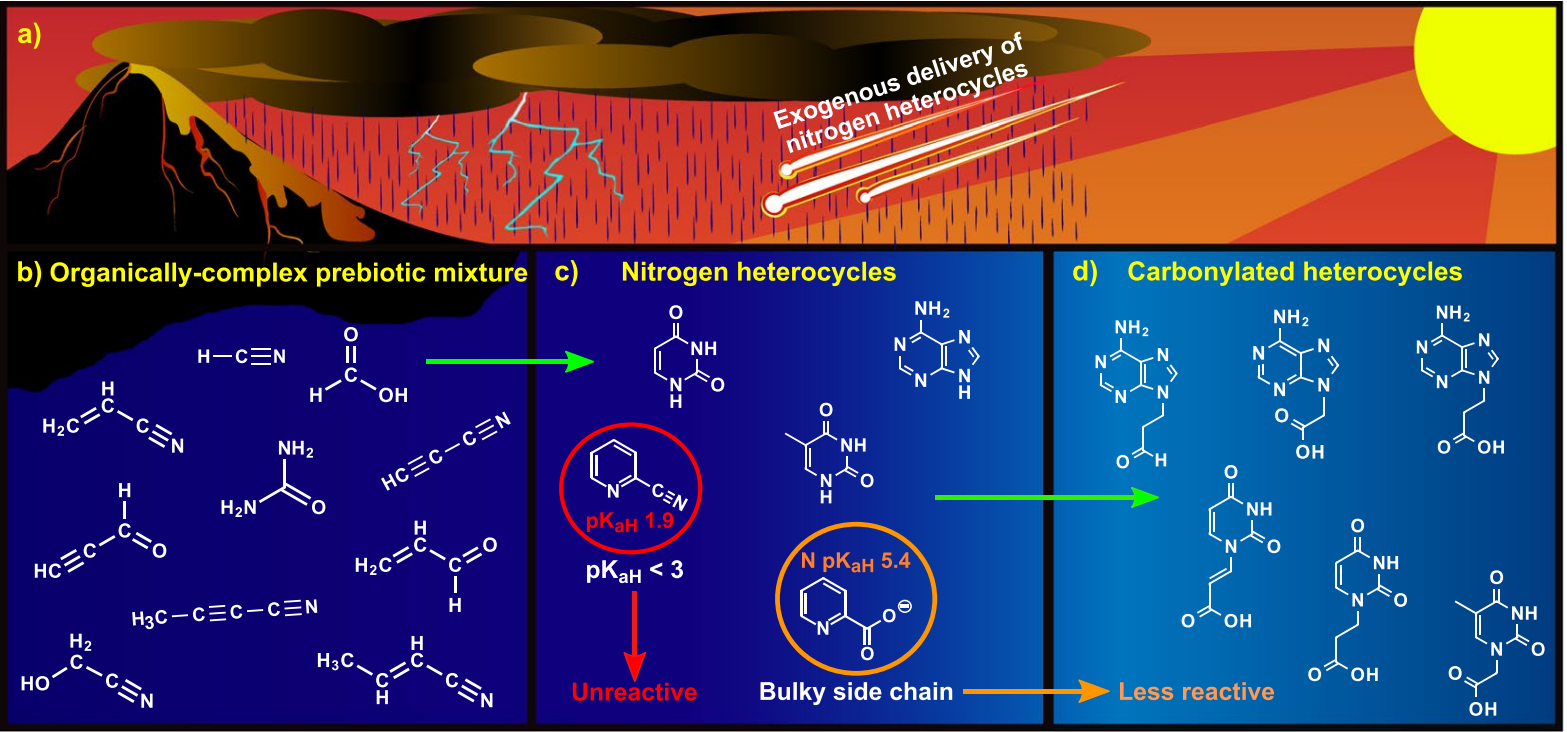
The chemical evolution of N-heterocycles into plausible pre-RNA monomers in aqueous solutions on early Earth. (**a**) N-heterocycles may have been delivered to the early Earth by meteorites^4,19^. Alternatively, electric discharges through either a reducing or neutral atmosphere produce (**b**) a complex mixture of organics that can combine to form (**c**) N-heterocycles^2,3,17,46^. Strongly acidic N-heterocycles (as indicated by low pK_aH_), such as those with cyano and amide groups conjugated to the ring, deactivate the N-heterocycle for electrophilic attack. Similarly, bulky side chains such as carboxylates also decrease the nucleophilicity of the ring nitrogen, and thus the heterocycle’s reactivity in complex mixtures. (**d**) Organics formed in electric discharges readily react with a wide range of N-heterocycles to form adducts with a carbonyl side chain, which can serve as precursors for PNA monomers.

Here we report plausible prebiotic pathways for the robust formation of carbonylated N-heterocycles in highly complex organic mixtures, which are expected to be present on early Earth (Fig. 5). The majority of these carbonylated heterocycles are formed by a wide range of N-heterocycles via both a Strecker-like synthesis with glycolonitrile and Michael additions with 3-carbon acrylic and propiolic derivatives. Intriguingly, previous work has shown that Michael additions tend to primarily occur at the N1 and N9 position of pyrimidines and purines, respectively^42–45^ (Fig. 1, Supplementary Fig. S6), hence generating pyrimidine and purine carbonylated heterocycles capable of forming Watson-Crick base pairs. Notably, strongly acidic N-heterocycles (e.g. cyanopyridines) were unreactive and those with bulky side chains conjugated to the ring (e.g. carboxylates, hydroxymethyl, etc.) were mostly inert in spark discharge mixtures; thus, once formed, these compounds are not as likely as other N-heterocycles to be incorporated into a primitive genetic polymer. Conversely, the majority of N-heterocycles which react readily with sugars were also reactive with a range of electrophiles in both reducing and neutral spark mixtures. Thus, although these heterocycles could have readily formed glycosides via ribose, their versatile reactivity with carbonylating organics may have impeded their incorporation into ribonucleoside polymers.

While it has been argued that a reducing atmosphere would have been more favorable for the origins of life on Earth^39^, we have demonstrated here that neutral atmospheres are just as conducive at producing carbonylated heterocycles. These results are in accordance with the consensus that the early Earth had a near neutral atmosphere^32^ and demonstrate how carbonylated heterocycles could have been produced from N-heterocycles on the early Earth under a broad range of atmospheric conditions.

Although we did not determine yields, the reproducibility of products in replicates (see Supplementary text, section 2.6) and with a large number of N-heterocycles in both heated and unheated reactions (see Supplementary Tables S3–S5) confirms that the formation of such adducts is robust. Furthermore, as the very organics that form N-heterocycles may also carbonylate them, the co-formation of N-heterocycles and their corresponding PNA precursors is prebiotically feasible. Given that there are many chemical pathways by which PNA precursors may form, and that these reactions occur under a broad range of conditions, it is possible that the carbonylated heterocycles identified here would have been readily available for the formation of PNAs on early Earth. PNAs are particularly interesting in regards to the origins of life as those with a backbone composed of AEG have been shown to form double helices with themselves and RNA and, importantly, are capable of auto-catalytic and cross-catalytic template-based replication^57,58^—characteristics that are important for any viable genetic polymer. Additionally, as PNAs are composed of N-heterocycles and amino acids, they provide a plausible avenue for the co-evolution of proteins and nucleic acids.

Intriguingly, the organics generated from spark discharges—including amino and hydroxy acids in addition to N-heterocycles and the derivatives of organics that carbonylate them (e.g. β-amino acids and glycine which are amine derivatives of Michael acceptors and glycolonitrile, respectively)—have been identified in meteorites^23,24^ and experiments modeling organic synthesis at hydrothermal vents^25^, Titan’s atmosphere^59^, as well as the hydrolysis of tholins expected on Titan and Triton^60,61^. In fact, glycolonitrile, acrylonitrile, cyanoacetylene, acrolein, and propiolaldehyde have all been detected in the interstellar medium^62–65^; some of these organics have also been identified in interstellar ice analogs (i.e. acrolein, glycolonitrile)^66,67^, comets (i.e. cyanoacetylene, propiolaldehyde)^68,69^, and Titan’s atmosphere (i.e. acrylonitrile and cyanoacetylene)^70,71^. Furthermore, AEG and diamino acids, both of which could serve as the backbone for PNAs, have been detected in interstellar ice analogs^72^; diamino acids have also been identified in the Murchison meteorite^73^. These observations illustrate that the reactants that produce carbonylated heterocycles and potentially PNA monomers form in a wide variety of environments. In consequence, the chemical evolution of N-heterocycles to carbonylated adducts in Miller-Urey mixtures, as described here, may be a common phenomenon in the Solar System, potentially extending the formation of PNA precursors from early Earth environments to meteorites and other planetary bodies.

## Materials and Methods

All glassware was thoroughly washed with ultrapure water, wrapped in aluminum foil, and heated at 480 °C for 8 hours to ensure removal of organic compounds. All solutions were stored in anoxic vials stoppered with gas-tight blue rubber butyl stoppers; solutions were prepared with 0.2 M N_2_-purged potassium phosphate buffer (pH 8.0) with sodium sulfide nonahydrate (0.006 g/L) to act as an oxygen scrub.

### Spark discharge experiments

A 1000 mL round bottom flask attached to an Electrotechnics BD50E Tesla coil and two tungsten electrodes (tips 1 cm apart) was used to conduct spark discharge experiments (Supplementary Fig. S1). The flask was filled with 250 mL of a 0.2 M phosphate buffer solution (pH 8.0) and injected (in bars) with either a reducing (0.4 N_2_, 0.1 CO_2_, 0.25 H_2_, 0.25 CH_4_) or neutral (0.5 N_2_, 0.5 CO_2_) gas mixture. The flask was placed inside a water bath (~5 °C) and sparked at 40 kV for 72 hours; the water bath was employed to maintain a constant temperature in the flask. To evaluate the reactivity of N-heterocycles in complex mixtures, 1 mL from a heterocycle stock solution was reacted with 2 mL aliquots of fresh spark product (resulting in an initial heterocycle concentration of 1 mM). The reaction mixture was either incubated at 80 °C for 1–7 days or frozen immediately and kept at −80 °C; the latter was done to determine whether (1) adducts formed in the absence of heating and (2) if heating facilitated their formation. Reactions were stopped by flash freezing with dry ice and stored at −80 °C until their analysis.

### Sample analysis

Samples were analyzed using a Thermo Scientific LTQ Orbitrap XL hybrid mass spectrometer equipped with an IonSense DART ion source (IonSense, Saugus, MA, USA). The resolving power of the orbitrap was set to 30,000 (at *m/z* 400) and mass spectra were collected over a range of *m/z* 50–500. The mass spectrometer was run under positive ion mode and the DART ion source operated at 350 °C with He gas; guanine was analyzed at 450 °C. N-heterocycles that did not ionize were re-analyzed in negative mode using a DART ion source temperature of 450 °C. Due to the complexity of the reaction mixtures, a targeted approach was used to identify adducts (see Supplementary Table S2). Heterocycle adducts were identified using molecular formulae as determined by accurate mass measurements (typically <5 ppm mass error) and comparison to controls that were processed in parallel. Some adducts were confirmed by incubating N-heterocycles (1 mM) with the predicted reactant (1 mM) and matching product ion spectra (MS/MS) along with comparison to reference standards when available. NMR spectra were obtained using a Bruker Avance III HD 500 spectrometer at 50.7 MHz and 298 K. Additional experimental details are described in the SI text (see *Supplementary text*, *section 1*.*0*).

## Supplementary information

Supplementary Info

## Data Availability

Individual mass spectra will be deposited at the public Penn State data commons (http://www.datacommons.psu.edu) upon publication. All other data generated or analyzed during this study are included in this published article (and its Supplementary Information files).
