# Supplementary material for: Nitrogen heterocycles form peptide nucleic acid precursors in complex prebiotic mixtures
Source: Sci Rep. 2019 Jun 26;9:9281. doi: 10.1038/s41598-019-45310-z (PMC6594999; doi:10.1038/s41598-019-45310-z)
Supplement: Supplementary file 1 — Supplementary Info [file 41598_2019_45310_MOESM1_ESM.pdf]

Supplementary Information for

Nitrogen heterocycles form peptide nucleic acid precursors in complex prebiotic mixtures

Laura E. Rodriguez[a], Christopher H. House[a,*], Karen E. Smith[a,b], Melissa R. Roberts[b] and Michael P. Callahan[b,c,*]

[a]Department of Geosciences and Penn State Astrobiology Research Center, The Pennsylvania State University, 220 Deike Building, University Park, PA 16802, USA; [b]Department of Chemistry and Biochemistry, Boise State University, 312 Science Building Boise, ID 83725, USA; [c]Astrochemistry Laboratory and Goddard Center for Astrobiology, National Aeronautics and Space Administration Goddard Space Flight Center, Greenbelt, MD 20771, USA

Corresponding Authors: Christopher H. House (chrishouse@psu.edu) and Michael P. Callahan (michaelcallahan914@boisestate.edu)

This PDF file includes:

Supplementary text
Figs. S1 to S25
Tables S1 to S7
References for SI

Table of Contents

1.0 Materials and Methods……………………………………………………………………3

1.1 Chemical Solutions and Glassware……………………………………………...3
1.2 Sample Analysis by DART-MS………………………………………………... 4
1.3 Characterizing Spark Adducts…………………………………………………..5

2.0 Additional Results and Discussion………………………………………………...6

2.1 The missing formaldehyde-heterocycle adduct……………………………………6
2.2 N-heterocycle reactions with glycolonitrile………………………………………. 7
2.3 The nucleophilic side chain resulting from nitrosation and cyanamide/urea……... 9
2.4 Reaction trends of carbonylation inferred from the results of previous studies…...9
2.5 The possibility for a one-pot synthesis of carbonylated heterocycles in spark mixtures…………………………………………………………………………...13
2.6 Variability between replicates of Miller-Urey spark discharge experiments…….16

3.0 SI Tables…………………………………………………………………………...17

4.0 SI Figures…………………………………………………………………………57

5.0 SI References……………………………………………………………………... 82

1.0 Materials and Methods

1.1 Chemical Solutions and Glassware

All reagents were purchased with purities of 95% to ≥99% and were used without further purification. The nitrogen heterocycles (N-heterocycles) were purchased from Schircks Lab (Switzerland) [6-hydroxymethylpterin], Alfa Chemistry [3,5-dimethyluracil], Maybridge [isoguanine], BeanTown Chemical [barbituric acid], Oxchem [2-hydroxymethylpyridine, 2-hydroxypyrimidine hydrochloride], Tokyo Chemical Industry [ammeline, ammelide], and Toronto Research Chemicals [6-biopterin]; all other N-heterocycles were obtained from Sigma-Aldrich, Acros Organics, or Alfa Aesar. For NMR analysis, Deuterated D_6-dimethyl sulfoxide (D_6-DMSO) and $^{15}N_2$-uracil were purchased from Sigma-Aldrich. Reagents to test the reactivity of N-heterocycles in isolated reactions were bought from Tokyo Chemical Industry [acrylonitrile (stabilized with 4-methoxyphenol)], Santa Cruz Biotechnology, Inc. [propiolaldehyde], and Sigma-Aldrich [Formic acid (95%), NaCN, glycolic acid nitrile solution (i.e., glycolonitrile, ~70% in water containing ~0.5% phosphoric acid stabilizer), acrylamide, acrylic acid (stabilized with 200 ppm 4-methoxyphenol), crotonitrile (mixture of *cis* and *trans*), propiolamide, propiolic acid, and 2-butynoic acid]. Ultrapure N_2 gas (99.999%), Bone-Dry grade CO_2 gas (99.9%), ultrapure 4:1 N_2:CO_2 gas mixture (99.999%), ultrapure CH_4 gas (99.999%), and ultrapure H_2 gas (99.999%) were used to conduct spark discharge experiments. This study exclusively used ultrapure doubly distilled water (18 MΩ·cm) from a Barnstead NANOpure Infinity ultrapure water system; prior to making any solutions, the ultrapure water was deoxygenated by sparging with ultrapure N_2 gas and subsequently autoclaved.

Stock solutions and samples were exclusively handled in an anoxic chamber filled with nitrogen gas. Reactions were carried out in vials that had been triply flushed with ultrapure nitrogen, sealed and crimped with air-tight blue rubber butyl stoppers, and subsequently autoclaved. Stock solutions of N-heterocycles were stored at -80 °C and used within two weeks. Heterocycles that were insoluble were thoroughly mixed and gently heated to promote dissolution prior to distributing the solutions into reaction vials.

1.2 Sample Analysis by DART-MS

A Thermo Scientific LTQ Orbitrap XL hybrid mass spectrometer equipped with a direct analysis in real-time (DART) ion source (IonSense, Saugus, MA, USA) was used to rapidly analyze the chemical profile for each complex reaction mixture. 5 μL aliquots of each sample were spotted in a single window on a 12-sample QuickStrip™ sample card (Ionsense, Saugus, MA, USA) in a positive pressure HEPA-filtered laminar flow bench; note that this was the first time that our samples were exposed to air. After the spots dried, the card was mounted onto a linear rail system (IonSense, Saugus, MA, USA) set to move at 0.2 mm/s. The DART nozzle was spaced at 1.5 mm from the sample card; the sample card was approximately 1.1-1.2 mm from the orbitrap mass spectrometer inlet. Spectra were acquired for 0.55 minutes per sample.

External mass calibration was performed using an electrospray ionization source and a mixture of caffeine, MRFA (L-methionyl-arginyl-phenylalanyl-alanine acetate hydrate) peptide, and Ultramark 1621 in an acetonitrile-methanol-water solution containing 1% acetic acid. After

mass calibration, the DART source was installed, and the mass accuracy was checked using a standard solution of quinine in methanol. Polysiloxane was used as a lock mass in positive ion mode. To ensure adducts detected were from heterocycles incubated with spark generated organics, and not contaminants, potential adducts had to meet strict criteria: (1) the absolute abundance of the product in the reaction mixture had to be at minimum a magnitude larger than a peak of the same *m/z* in the controls and (2) had to have an absolute intensity of at least 10^3 counts.

Appended functional groups of target adducts were determined by subtracting the chemical formula of the starting heterocycle from the chemical formula of identified adducts predicted by Xcalibur software (typical accuracy: <5 ppm error). Product ion spectra (MS/MS) were also acquired using higher-energy collision dissociation (HCD) of 0-120% with an isolation width of 1-2 Da. Structures of daughter ions in the product ion spectra were generated using Thermo Scientific Mass Frontier 7.0 software in many cases.

1.3 Characterizing Spark Adducts

To elucidate the structure and reaction pathway of spark-heterocycle adducts, we studied the reactivity of several heterocycles (1 mM) incubated at 5, 30, or 80 °C for 1-3 days with the organic (1 mM) predicted to have reacted with it in the spark reaction mixture; to test whether (1) reactions proceed in unheated reaction mixtures and (2) if heating facilitates their formation (reflected in their ion intensities), reaction mixtures were also frozen immediately and stored at -80 °C until analysis. The following organics were reacted with heterocycles: formic acid, NaCN, glycolonitrile, acrylonitrile, acrylamide, acrylic acid, crotonitrile, propiolaldehyde, propiolic acid, propiolamide, and 2-butynoic acid. To determine whether adducts in isolated reactions were the

same as those found in reactions with spark discharge organics, MS/MS spectra of target adducts (with sufficient abundance) were obtained via the tandem mass spectrometry settings described previously.

Uracil-spark adducts were further characterized to determine the preferential site on uracil (C5, N1, or N3) for adduct formation. Solutions of 1 mM 1,3-dimethyluracil and 1 mM 3,5-dimethyluracil were reacted with 1 mM glycolonitrile, acrylic acid, or propiolic acid at 30 or 80 °C for 72 hours; reaction mixtures were also frozen immediately and stored at -80 °C until analysis. Additionally, the reaction between $^{15}N_2$-uracil (Sigma-Aldrich) and acrylic acid was investigated using ^{15}N-NMR. For NMR experiments, $^{15}N_2$-uracil (0.04 g) was dissolved with acrylic acid (0.04 mL) in D_6-DMSO (0.56 mL), heated to 100 °C for 3 hours, and analyzed the same day. ^{15}N-spectra were also collected for the following standards: $^{15}N_2$-uracil (0.04 g) and uracil-N-1-propanoic acid (0.1 g, 10k scans) in D_6-DMSO (final volume 0.6 mL). Spectra were recorded using a Bruker Avance III HD 500 spectrometer at 50.7 MHz and 298 K.

2.0 Additional Results and Discussion

2.1 The missing formaldehyde-heterocycle adduct

Uracil, adenine, and other N-heterocycles are known to react readily with formaldehyde[1]. Formaldehyde is one of the major products from a spark discharge reaction[2], thus it came as a surprise that only two N-heterocycles were observed to form adducts with a hydroxymethyl group (Het-CH_2OH). Six heterocycles did produce a N-heterocycle dimer bifurcated by a methylene group (Het-CH_2-Het), which could also be taken as evidence for reactions involving formaldehyde. For example, uracil and cytosine[3] and 2-aminopyridine have been shown to form these dimers with

formaldehyde[4]. One reason that only a few heterocycles reacted with formaldehyde could be that the majority of the formaldehyde generated in the spark was tied up as glycolonitrile (due to reaction with hydrogen cyanide), and therefore unavailable to the N-heterocycles[2,5]. Another possible explanation is that heterocycles with hydroxymethyl groups were rapidly attacked by other nucleophiles present in the mixture (e.g., 5-hydroxymethyluracil (5-HMU) reacts further with many different nucleophiles as observed here (Tables S3-S4) and previous work by Robertson and Miller[1]). Alternatively, it is possible that heterocycle-formaldehyde adducts fragmented during mass spectrometry analysis, making them difficult to detect; for example, the base peak in the mass spectrum of a 5-HMU standard is a fragment at *m/z* 125.0346 corresponding to $C_4H_3O_2N_2$-CH_2^+ and not the molecular ion (*m/z* 143.0451 = $[C_4H_3O_2N_2$-$CH_2OH + H]^+$).

2.2 N-heterocycle reactions with glycolonitrile

We studied the reactivity of guanazole (typically 30 mM) incubated with equimolar amounts of glycolonitrile at different temperatures (80, 100, and 120 °C) and reaction times (1-3 days) (Fig. S5). These reactions were analyzed by direct infusion into an electrospray ionization quadrupole time-of-flight mass spectrometer operating in positive ion mode to acquire full-scan mass spectra and product ion spectra (MS/MS).

For the reaction at 80 °C and 72 h, a significant peak in the mass spectrum was assigned to the guanazole-CH_2-CN adduct. In addition, two more peaks in the mass spectrum were assigned to guanazole-CH_2-(CO)NH_2 and guanazole-CH_2-COOH adducts. Product ion spectra for these three heterocycle adducts were identical to those acquired in reactions involving guanazole with Miller-Urey organic mixtures synthesized from a reducing atmosphere indicating that

glycolonitrile is present in the Miller-Urey organic mixtures and reactions with glycolonitrile would still occur in highly complex organic mixtures. Heating guanazole+glycolonitrile at 80 °C in an open (rather than closed) system facilitated the evaporation of the aqueous solvent, which led to a slight increase in the guanazole-CH_2-CN adduct and a decrease in guanazole-CH_2-(CO)NH_2 and guanazole-CH_2-COOH adducts (relative to guanazole). At higher concentrations (1 M), this effect is more pronounced.

At 100 °C, the guanazole-CH_2-CN adduct steadily decreases as the reaction proceeds from one to three days while the guanazole-CH_2-(CO)NH_2 and guanazole-CH_2-COOH adducts both gradually increase. At 120 °C, the guanazole-CH_2-CN adduct is no longer detected and only the guanazole-CH_2-(CO)NH_2 and guanazole-CH_2-COOH adducts are present in the 24 and 48 h reactions. At the longest reaction time (72 h), only the guanazole-CH_2-COOH adduct is measured. These results suggest that the hydrolysis of the nitrile functional group to an amide group and eventually to a carboxylic acid group is sensitive to both reaction temperature and time. In complex organic mixtures, these heterocycle adducts may undergo further reactions.

We also observed similar adducts with other N-heterocycles reacting with glycolonitrile in isolated reactions and in complex Miller-Urey organic mixtures, which suggests that this reaction pathway is relatively robust and, therefore, more prebiotically plausible. It's also interesting to note that isolated reactions of N-heterocycles with glycolamide (a spontaneous hydrolysis product of glycolonitrile) under the same solution conditions did not produce the anticipated adduct of heterocycle-CH_2-(CO)NH_2, which highlights the selective nature of some reactions and that certain precursors may be necessary to form certain heterocycle adducts.

2.3 The nucleophilic side chain resulting from nitrosation and cyanamide/urea

In general, carbonyl carbons can be thought of as electrophilic and thus susceptible to nucleophilic attack—the exception being urea (NH_2-CO-NH_2) and its substituted derivatives (R-NH-CO-NH_2) as the two amines increase the electron density about the carbonyl carbon, decreasing its electrophilicity. In fact, the terminal amine of these groups is a good nucleophile (as seen in studies of urea), thus the side chains resulting from nitrosation and subsequent nucleophilic substitution with urea (or cyanamide + hydrolysis to the urea derivative) are expected to be nucleophilic. Notably, the corresponding acids (R-NH-CO-OH) resulting from hydrolysis of urea derivatives spontaneously decarboxylate (R-NH_2 + CO_2)[6,7] and—in regards to heterocycle-urea adducts—regenerate the parent N-heterocycle.

2.4 Reaction trends of carbonylation inferred from the results of previous studies

Table S7 lists yields of carbonylated nucleobases derived from previous publications. Although the reactions were conducted under various conditions, general trends can still be ascertained by comparing yields. Notably, nucleobases form adducts via Michael additions more efficiently than reactions with formaldehyde + HCN (the latter of which combine to form glycolonitrile). For example, when Cleaves (2002)[8] incubated nucleobases (1 or 22 mM) with acrolein (10 mM, pH 9, 15 °C, up to 60 min) and Crippa et al. (1993)[9] incubated them with acrylonitrile (1.1 M, pH 8, 50 °C, 24-336 h), the authors found that adducts with 3-carbon long carbonyl side chains at the N9 position for purines and N1 position for pyrimidines were generated in 13-70% and 10-18% yields, respectively; yet when Nelson et al. (2000)[10] reacted the nucleobases (1 mM) with HCN + CH_2O (each at 30-70 mM, pH 9.6, 25 °C) for 1 year the N9-

purine and N1-pyrimdine carbonylated nucleobases were generated only in 0.04-0.15% yields. Although, it should be noted that the latter study used an excess of both formaldehyde and HCN under conditions optimal for their self-polymerization. Thus, the formation of carbonylated adducts would have had to compete against the more rapid, irreversible polymerization of CH_2O and HCN. In fact, experimental work has shown that the rate of HCN polymerization increases with the square of HCN concentration[11]. It is possible that under more realistic prebiotic conditions (1 mM – 10 mM each, pH 6-8) the yields for the acetic acid adducts may be much higher. Given the problems with polymerization, it seems possible that reactions of nucleobases with glycolonitrile (a one-step reaction) may generate the acetic acid adducts in higher yields than the two-step reaction involving formaldehyde + HCN. Alternatively, a step-wise reaction where the nucleobases are first reacted with formaldehyde and then HCN may be more favorable, however given the reactivity of uracil with formaldehyde the reaction is expected to generate higher yields of the C5-carbonylated adducts[1] (e.g. 5-HMU + HCN → U-C5-acetonitrile in >99% yields at pH 7, 100 °C, 25 h); adenine and guanine would likely generate a mixture of N9- and either N6- (for adenine) or N2 (for guanine) isomers as prior work showed that adenine + CH_2O → N6 and N9-adducts and guanosine + CH_2O → N2-adducts[12,13]. Notably both studies found that over time oligomers of adenine/adenosine/guanosine connected by a methylene bridge (from CH_2O) formed, demonstrating that nucleophilic attack of the Het-CH_2OH group by compounds such as $^-$CN is possible. Unfortunately, Nelson and colleagues[10] did not discuss the yields obtained for carbonylated adducts with the side chains at positions other than the N9 for purines and N1 for pyrimidines. Given this, a Strecker-like synthesis with HCN, CH_2O, or glycolonitrile may generate higher yields of 2-carbon long carbonyl side chains than Table S7 suggests, however given the nucleobases' reactivity with formaldehyde, it remains unlikely that these reactions would generate

higher yields of carbonylated side chains at positions N9 for purines and N1 for pyrimidines compared to Michael additions.

Carbonylated nucleobases with three-carbon long side chains are likely to be produced at higher yields than the two-carbon carbonylated adducts for several reasons: (**1**) they are generated via Michael additions which are regioselective, increasing yields for a particular isomer (the N9 adduct for purines and N1 for pyrimidines); (**2**) they involve Michael acceptors which do not readily polymerize in basic (pH 8-9), anoxic solutions (although they do polymerize when exposed to oxygen, UV light, or radicals); (**3**) they are a one-step reaction, whereas carbonylated adducts generated via CH_2O + HCN are susceptible to inhibition due to nucleophiles besides HCN (e.g. NH_3, glycine, methanethiol, etc.) attacking the formaldehyde adduct (we saw this with 5-HMU in the spark and this was also the subject of a paper by Robertson and Miller[1]); and (**4**) there are various pathways to generating three-carbon long carbonyl side chains via the nucleobases: reactants such as acrylonitrile, crotonitrile, cyanoacetylene, acrolein, propiolaldehyde, etc. readily react regioselectively with the nucleobases.

Reaction trends between the various Michael additions can also be ascertained from the data summarized in Table S7. Notably, acrolein (which contains a terminal aldehyde group) is more reactive with the nucleobases than other Michael acceptors containing a vinyl group, but different electron withdrawing group (i.e. H_2C=CH-X, where X = CHO (acrolein); CN (acrylonitrile); $COCH_2CH_3$ (ethylacrylate))[8,9]. In accordance with this, Cleaves[8] found that the reaction rate between adenine or uracil and acrylonitrile was 30,000 and 3,000 times slower compared to their reaction with acrolein. Given this pattern, propiolaldehyde is likely more reactive with the nucleobases than cyanoacetylene (HC≡C-X, X = CHO (propiolaldehyde); X = CN (cyanoacetylene)). This is consistent with the results obtained by Johnson and colleagues[14],

where they observed that under the same conditions, carbonylated thymine is generated in slightly higher yields via propiolaldehyde (76.7%) than cyanoacetylene (62%).

Two additional reaction trends can be ascertained from Table S7. For one, Crippa and colleagues[9] demonstrate that β-alkylated Michael acceptors (i.e. ethylacrylate) are less reactive than their non-methylated counterparts (e.g. ethylcrotonate), likely due to steric hinderance at the reaction site; accordingly, Cleaves[8] also found that under the same conditions thymine reacted more with acrolein than crotonaldehyde. Secondly, although we cannot compare the yields between Michael acceptors with alkyne (e.g. cyanoacetylene) vs vinyl groups (e.g. acrylonitrile) as each were conducted under significantly different conditions, previous work by Furukawa et al.[15] observed that cyanoacetylene was more reactive than acrylonitrile; with the former capable of reacting with the neutral nucleosides, but the latter only reacting with deprotonated nucleosides. The authors inferred that this difference in reactivity was due to the triple bond of cyanoacetylene vs the double bond in acrylonitrile as triple bonds are typically much more susceptible to nucleophilic attack than double bonds.

Given the above observations, if the carbonylating spark organics were present in equal amounts in the spark mixture (and disregarding competing nucleophiles), we would expect the most abundant carbonylated N9-purines and N1-pyrimidines to be those generated from (in decreasing order): propiolaldehyde > acrolein, cyanoacetylene > acrylonitrile, methyl-cyanoacetylene > crotonitrile > glycolonitrile/CH_2O + HCN.

2.5 The possibility for a one-pot synthesis of carbonylated heterocycles in spark mixtures

Intriguingly, a one-pot synthesis for the carbonylated nucleobases is possible in the Miller-Urey spark mixtures. It is known that these spark discharges generate cyanoacetylene, acrylonitrile and urea[2,16]—the precursors to cytosine, and uracil—in addition to NH_4^+ and ^-CN, which upon polymerization, produces adenine, guanine, hypoxanthine, and xanthine[17,18]. Accordingly, the nucleobases have been detected in Miller-Urey spark discharge mixtures[19,20], and in our own mass spectra of the reducing spark mixtures we identified peaks with masses consistent of 2,4-diaminopyridine, cytosine, uracil, thymine, adenine, hypoxanthine, and xanthine; only a mass consistent with thymine was detected in the neutral spark. After their formation in the spark mixture, N-heterocycles could undergo a subsequent reaction with the carbonylating reactants. In fact, we identified several masses corresponding to carbonylated adducts in the reducing spark mixture that were not present in the buffer controls or blanks and without the addition of the N-heterocycle: note that only adducts in the reaction mixture with an intensity greater than 10x that in the spark mixture alone were counted as products and included in Tables S3, S4, and S6.

Previous work gives some indication of the types of yields we may expect from a one-pot synthesis of carbonylated adducts in mixtures akin to spark-discharges. For example, Nelson et al.[10] found that when HCN was polymerized in glycine solutions (pH 9.8, 80 °C, 18 h), adenine-N9-acetic acid (0.0062% yield) and guanine-N9-acetic acid (0.011% yield) were generated in slightly higher yields than their corresponding nucleobases; the N9 adduct was also the dominant isomer identified. The authors predict that carbonylated nucleobases were generated via incorporation of glycine during HCN oligomerization. If this is indeed the case, then the formation of purine-N9-propanoic (A-CH_2-CH_2-COOH) is possible as β-alanine is also generated during HCN oligomerization[21]. In a similar reaction, the polymerization of neat formamide with catalytic

minerals (e.g. TiO_2, montmorillonites, 160 °C, 48 h) generates N9,N6-diformyl-adenine (0.04% yield) and N9-formyl-purine (14-21%) in amounts comparable to adenine and purine, respectively[22,23]; the authors predict that the formylated adducts were generated via formylation of the corresponding nucleobases by formic acid or ammonium formate. As HCN and its hydrolysis products, formamide and formic acid, are amongst the most abundant organics produced in spark discharges, complex mixtures generated from HCN and formamide polymerization (and thus the yields obtained for the carbonylated heterocycles) may be similar to those from spark discharges.

A one-pot synthesis for carbonylated pyrimidines in complex mixtures has not been studied, however it was found that polymerization of NH_4CN generates at least two compounds containing uracil[24,25]. Furthermore, Nelson et al[10] showed that carbonylated pyrimidines can be generated via pyrimidine precursors. Specifically, the authors demonstrated that when cyanoacetaldehyde is incubated with hydantoic acid (i.e. urea-CH_2-COOH) rather than urea, cytosine-N1-acetic acid (rather than cytosine) and uracil-N1-acetic acid (rather than uracil) are generated in good yields (1.8-18%); when cyanoacetaldehyde was incubated with both urea and hydantoic acid, cytosine and the cytosine-N1-acetic acid adduct were generated in 8:1 ratios. As urea is a good nucleophile, it can also undergo Michael additions. For example, when urea is incubated with cyanoacetylene, propiolic acid, or acrylonitrile it generates a urea adduct similar to hydantoic acid that, upon cyclization, forms cytosine and uracil[26–28]. Ferris et al. (1968)[26] demonstrated that when urea undergoes carbonylation, the rate of cyclization (and hence formation of N-heterocycles like cytosine or uracil) increased at higher temperatures and higher pH; cyclization was much slower at room temperature and in solutions at pH ~7-8. If the urea adduct does not cyclize, the primary amine can undergo a subsequent reaction with a carbonylating organic (e.g. propiolic acid, acrylonitrile, glycolonitrile, etc), generating a bis-urea adduct (X-NH-

CO-NH-X, where X = carbonylated side chain) that upon cyclization can form a N1-carbonylated pyrimidine; the latter was demonstrated by Nelson et al. (2000)[10] with hydantoic acid and cyanoacetaldehyde. Remarkably, even if the urea adduct were to cyclize at much greater rates than formation of the bis-urea adducts, the resulting pyrimidine nucleobase would still be exposed to any residual carbonylating reactant and could readily form the nucleobase adduct as demonstrated in our results from N-heterocycles incubated with spark mixtures.

In summary, previous studies have demonstrated the feasibility of (**1**) generating nucleobases in Miller-Urey mixtures, (**2**) the reactivity of nucleobases with Michael acceptors in isolated reactions, and (**3**) the possibility for a one-pot synthesis of carbonylated adducts in complex mixtures similar to those that may be obtained via spark discharge experiments. In our reaction mixtures we identified masses corresponding to nucleobases as well as their adducts, increasing the credibility for a one-pot synthesis of carbonylated nucleobases. Moreover, we found that a wide-range of N-heterocycles, including the biological nucleobases, readily react with organics within the spark mixture under a broad range of conditions; this last observation serves three points: (**1**) that nucleophiles generated in the spark mixture (e.g. NH_3, glycine, ethylenediamine, urea, etc.) did not sequester all of the carbonylating reactants, (**2**) the very reactants that generate carbonylated nucleobases can also serve as precursors to the nucleobases—this suggests that after they form nucleobases may be readily carbonylated, and (**3**) there is a wide-range of mechanisms by which PNA precursors can form in complex mixtures: carbonylated heterocycles can be generated via either the heterocycles or their precursors reacting with various carbonylating organics that are similar in structure (e.g. acrylonitrile vs acrolein vs cyanoacetylene, etc.). Together, these observations suggest that the formation of carbonylated heterocycles in Miller-Urey spark mixtures is robust.

2.6 Variability between replicates of Miller-Urey spark discharge experiments

A total of three spark experiments were conducted under a reducing atmosphere; two experiments were performed under the neutral atmosphere. To test the reproducibility of our results, we investigated how the reactivity of 12 N-heterocycles changed between replicates of the reducing and neutral spark-discharge mixtures. We found that there is some variability in the adducts generated between replicates of Miller-Urey spark discharge experiments (in terms of the specific adducts identified and the intensity at which they were observed), but that overall trends, namely—(A) the reoccurrence of carbonylated adducts, (B) the scarcity of nucleophilic, alcohol, and sugar adducts, and (C) the major reactions described in Figure 3 (*main manuscript*)—remained the same. Note that the degree of variability appears to be more substantial for the neutral spark mixture. This observation is consistent with previous studies where the authors noted considerable variability of the specific products formed and their absolute abundances between duplicates of their Miller-Urey spark discharge experiments[29,30]. In these studies the authors proposed that the variability between replicates is due to minor fluctuations in the spark apparatus; in particular the study by Schlesinger and Miller[29] noted that the power output of the spark generators varied over the duration of the experiment. Indeed, we observed that the visible intensity of the spark decreased from the start to end of each experiment (72 h) and found it necessary to recalibrate the power outage (42 kV) given by the generator prior to starting each run. As the neutral spark experiments were conducted *after* the reducing spark experiments were completed, it is probable that there were greater power fluctuations from the start to end of the neutral spark experiments, which may explain why we observed a greater degree of variability between replicates. These observations are consistent with the previous assertation that BD-50E Tesla coils may not be suitable for prolonged use[31].

3.0 SI Tables

Table S1. Nitrogen heterocycles selected for study. References are listed by number.

Pyridines	Heterocycle	Prebiotic Synthesis Demonstrated	Identified in Meteorites	Forms a nucleoside with ribose
**1**	2-aminopyridine	-	-	-
**2**	4-aminopyridine	-	-	-
**3**	3-aminopyridine	-	-	-
**4**	3-hydroxymethylpyridine	-	-	-
**5**	4-hydroxymethylpyridine	-	-	-
**6**	2-hydroxymethylpyridine	-	-	-
**7**	2-pyridinecarboxylic acid	32	33	-
**8**	3-pyridinecarboxylic acid	32	33	-
**9**	4-pyridinecarboxylic acid	32	33	-
**10**	4-cyanopyridine	34	-	-
**11**	3-cyanopyridine	34	-	-
**12**	2-cyanopyridine	34	-	-
**Pyrimidines**	**Heterocycle**	**Prebiotic Synthesis Demonstrated**	**Identified in Meteorites**	**Forms a nucleoside with ribose**
**13**	2-aminopyrimidine	35, 36	-	-
**14**	4-aminopyrimidine	35, 36	-	-
**15**	2,4-diaminopyrimidine	20, 35, 37	-	-
**16**	2,4,6-triaminopyrimidine	38	-	39
**17**	Cytosine	19, 20, 35–37, 40–43	-	
**18**	Isocytosine	35–37, 41, 43	-	-
**19**	4-hydroxy pyrimidine	25, 36, 40, 41, 43	-	-
**20**	2-hydroxy pyrimidine	35, 36, 41	-	44, 45
**21**	Uracil	19, 20, 35-37, 41-43	46–48	
**22**	Barbituric Acid	20, 35	-	49, 50
**23**	Thymine	19, 22, 36, 43, 51	-	
**24**	5-hydroxymethyluracil	1, 22, 51	-	-
**25**	Orotic Acid	43	-	-
**Triazines**	**Heterocycle**	**Prebiotic Synthesis Demonstrated**	**Identified in Meteorites**	**Forms a nucleoside with ribose**
**26**	3-amino-1,2,4-triazine	-	-	-
**27**	Guanazole	52	-	52
**28**	Melamine	20, 38, 53	-	54
**29**	Ammeline	20, 53	-	-
**30**	Ammelide	20	-	-
**31**	s-Triazine	-	55	-
**32**	Urazole	52	-	52
**33**	6-azauracil	-	-	
**34**	Cyanuric Acid	20	-	-
**Purines**	**Heterocycle**	**Prebiotic Synthesis Demonstrated**	**Identified in Meteorites**	**Forms a nucleoside with ribose**
**35**	2-aminopurine	56, 57	-	-
**36**	Adenine	18, 19, 20, 22, 40, 41, 43, 53, 57, 58	46, 59, 60	61, 62
**37**	2,6-diaminopurine	18, 41, 53	59	-
**38**	Isoguanine	57, 63	-	-
**39**	Guanine	18, 19, 40, 42, 43, 53, 57, 58	46, 59, 60	61
**40**	Hypoxanthine	40, 57, 58, 64	46, 59, 60	61
**41**	3,7-dimethylxanthine	-	-	-
**42**	Xanthine	42, 57	46, 59, 60	61
43	1,3-dimethylxanthine	-	-	-

Pteridines	Heterocycle	Prebiotic Synthesis Demonstrated	Identified in Meteorites	Forms a nucleoside with ribose
**44**	6,7-dimethylpterin	-	-	-
**45**	6-hydroxymethylpterin	-	-	-
**46**	2,4-diamino-6-hydroxymethylpteridine	-	-	-
**47**	Xanthopterin	65, 66	-	-
**48**	Isoxanthopterin	65, 66	-	-
**49**	Pterine	65	-	
**50**	6-Biopterin	-	-	-
**51**	Pterine-6-carboxylic acid	-	-	-
**52**	Lumazine	65, 66	-	-
**53**	Alloxazine	-	-	-

Table S2. List of adducts targeted for detection in heterocycle-spark reaction mixtures. Adducts that have the same structure as proposed pre-RNAs are noted in bold. Abbreviations: CH_2O (Formaldehyde); nuc. (nucleophilic).

Side Chain Category	Hypothetical Reactants (nucleophile vs electrophile)	Δ MW (Da)	Δ Elemental Composition	Side Chain Composition	Possible Adduct Structures (Het =heterocycle)
**Electrophilic Carbonyls**	Formic Acid (electrophile)	28	C O	C H O	Het-CHO 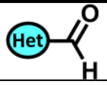
	Formic Acid + NH_3 (Schiff Base) *same mass as HCN (nuc. addition) (see Carbonyl)	27	C H N	C H_2 N	Het-CH=NH 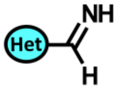
	Acetic Acid (electrophile)	42	C_2 H_2 O	C_2 H_3 O	Het-CO-CH_3 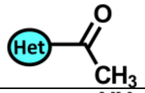
	Acetic Acid + NH_3 (Schiff Base)	41	C_2 H_3 N	C_2 H_4 N	Het-CNH-CH_3 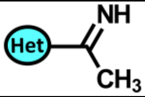
	HCN (nuc. substitution)	25	C N -H	C N	Het-CN 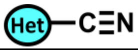
	HCN + H_2O (nuc. substitution)	43	C H O N	C H_2 O N	Het-$CONH_2$ 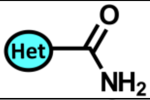
	HCN + 2 H_2O (nuc. substitution)	44	C O_2	C H O_2	Het-COOH 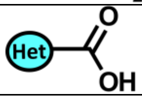
	HCN (nuc. addition) *same mass as adduct from Formic acid + NH_3 (see Carbonyl)	27	C H N	C N	H-Het-CN 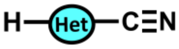
	HCN + H_2O (nuc. addition) *same mass as formamide (electrophile) (see Nucleophile)	45	C H_3 N O	C H_2 O N	H-Het-$CONH_2$ 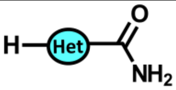
	HCN + 2 H_2O (nucleophilic addition)	46	C H_2 O_2	C H O_2	H-Het-COOH 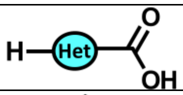
	Glycolonitrile (electrophile)	39	C_2 H N	C_2 H_2 N	Het-CH_2-CN 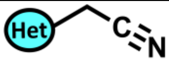
	Glycolonitrile + H_2O (electrophile) *Same mass as adduct from glycine condensation (see Nucleophile)	57	C_2 H_3 O N	C_2 H_4 O N	Het-CH_2-CO-NH_2 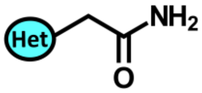
	Glycolonitrile + 2 H_2O (electrophile) *same mass as formic acid + methanediol (see Alcohol) ***PNA subunit**	58	C_2 H_2 O_2	C_2 H_3 O_2	Het-CH_2-COOH 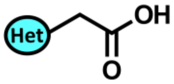
	Acrolein (electrophile, Michael Addition)	56	C_3 H_4 O	C_3 H_5 O	Het-CH_2-CH_2-CHO 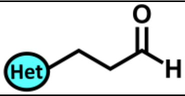
	Crotonaldehyde / Methacrolein (electrophile, Michael Addition)	70	C_4 H_6 O	C_4 H_7 O	Het-CH(CH_3)-CH_2-CHO<br>Het-CH_2-CH(CH_3)-CHO 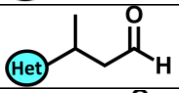
	Propiolaldehyde (electrophile, Michael Addition)	54	C_3 H_2 O	C_3 H_3 O	Het-CH=CH=CHO 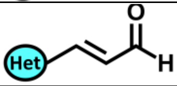
	Methylpropiolaldehyde* (electrophile, Michael Addition) *Refers to 2-methylpropiolaldehyde and 3-butyn-2-one (both of which are equally plausible)	68	C_4 H_4 O	C_4 H_5 O	2-Methylpropiolaldehyde: Het-C(CH_3)=CH-CHO<br>3-butyn-2-one: Het-CH=CH-CO-CH_3 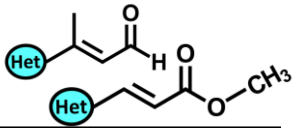

Side Chain Category	Hypothetical Reactants (nucleophile vs electrophile)	Δ MW (Da)	Δ Elemental Composition	Side Chain Composition	Possible Adduct Structures (Het =heterocycle)	
**Electrophilic Carbonyl**	Acrylonitrile<br>(electrophile, Michael Addition)	53	$C_3 H_3 N$	$C_3 H_4 N$	Het-CH_2-CH_2-CN	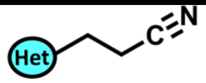
	Acrylonitrile + H_2O /<br>Acrylamide<br>(electrophile, Michael Addition)	71	$C_3 H_5 O N$	$C_3 H_6 O N$	Het-CH_2-CH_2-$CONH_2$	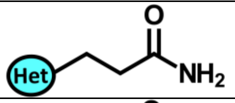
	Acrylonitrile + 2 H_2O /<br>Acrylic Acid<br>(electrophile, Michael Addition)	72	$C_3 H_4 O_2$	$C_3 H_5 O_2$	Het-CH_2-CH_2-COOH	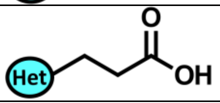
	Crotonitrile /<br>Methacrylonitrile<br>(electrophile, Michael Addition)	67	$C_4 H_5 N$	$C_4 H_6 N$	Het-CH(CH_3)-CH_2-CN<br>Het-CH_2-CH(CH_3)-CN	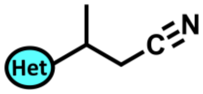
	Crotonitrile + H_2O or Crotonamide/<br>Methacrylonitrile + H_2O or<br>Methacrylamide<br>(electrophile, Michael Addition)	85	$C_4 H_7 O N$	$C_4 H_8 O N$	Het-CH(CH_3)-CH_2-$CONH_2$<br>Het-CH_2-CH(CH_3)-$CONH_2$	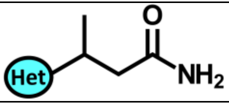
	Crotonitrile + 2 H_2O or<br>Crotonic acid /<br>Methacrylonitrile + 2 H_2O<br>or Methacrylic acid<br>(electrophile, Michael Addition)	86	$C_4 H_6 O_2$	$C_4 H_7 O_2$	Het-CH(CH_3)-CH_2-COOH<br>Het-CH_2-CH(CH_3)-COOH	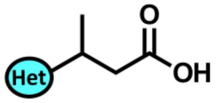
	Ethyl-acrylonitrile	81	$C_5 H_7 N$	$C_5 H_8 N$	Het-CH(CH_2CH_3)-CH_2-CN	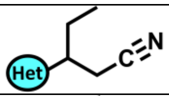
	Ethyl-acrylonitrile + H_2O /<br>Ethyl-acrylamide	99	$C_5 H_9 O N$	$C_5 H_{10} O N$	Het-CH(CH_2CH_3)-CH_2-<br>$CONH_2$	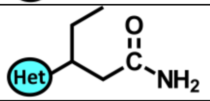
	Ethyl-acrylonitrile + 2 H_2O /<br>Ethyl-acrylic acid	100	$C_5 H_8 O_2$	$C_5 H_9 O_2$	Het-CH(CH_2CH_3)-CH_2-<br>COOH	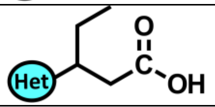
	Cyanoacetylene<br>(electrophile, Michael addition)	51	$C_3 H N$	$C_3 H_2 N$	Het-CH=CH-CN	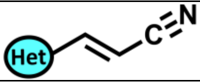
	Cyanoacetylene + H_2O<br>(electrophile, Michael Addition)	69	$C_3 H_3 O N$	$C_3 H_4 O N$	Het-CH=CH-$CONH_2$	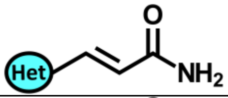
	Cyanoacetylene + 2 H_2O<br>(electrophile, Michael Addition)	70	$C_3 H_2 O_2$	$C_3 H_3 O_2$	Het-CH=CH-COOH	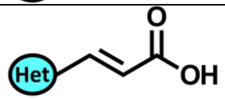
	Methylcyanoacetylene<br>(electrophile, Michael Addition)	65	$C_4 H_3 N$	$C_4 H_4 N$	Het-C(CH_3)=CH-CN	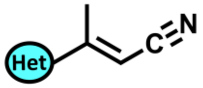
	Methylcyanoacetylene + H_2O<br>(electrophile, Michael Addition)	83	$C_4 H_5 O N$	$C_4 H_6 O N$	Het-C(CH_3)=CH-CO(NH_2)	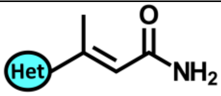
	Methylcyanoacetylene + 2 H_2O<br>(electrophile, Michael Addition)	84	$C_4 H_4 O_2$	$C_4 H_5 O_2$	Het-C(CH_3)=CH-COOH	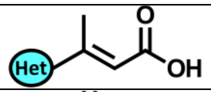
	Glycine nitrile (nuc. substitution)<br>*same mass as CH_2O + cyanamide<br>(see Nucleophile)	54	$C_2 H_2 N_2$	$C_2 H_3 N_2$	Het-NH-CH_2-CN	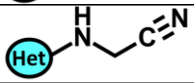
	Glycine amide (nuc. substitution)<br>*same mass as CH_2O + Urea<br>(see Nucleophile)	72	$C_2 H_4 O N_2$	$C_2 H_5 O N_2$	Het-NH-CH_2-$CONH_2$	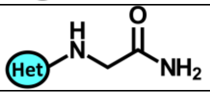

Side Chain Category	Hypothetical Reactants (nucleophile vs electrophile)	Δ MW (Da)	Δ Elemental Composition	Side Chain Composition	Possible Adduct Structures (Het =heterocycle)	
**Electrophilic Carbonyl**	Glycine (nuc. substitution) *same mass as CH_2O + Urea + H_2O (see nucleophile)	73	$C_2 H_3 O_2 N$	$C_2 H_4 O_2 N$	Het-NH-CH_2-COOH	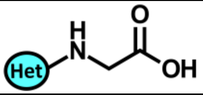
	Alanine (nuc. substitution)	87	$C_3 H_5 O_2 N$	$C_3 H_6 O_2 N$	Het-NH-CH(CH_3)-COOH	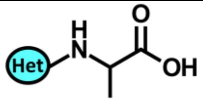
**Nucleophile/ Electrophilic Carbonyl**	Amino-glycine (nuc. substitution)	88	$C_2 H_4 O_2 N_2$	$C_2 H_5 O_2 N_2$	Het-NH-CH(NH_2)-COOH	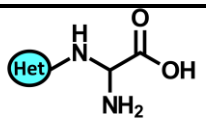
	Cyanogen (electrophile)	52	$C_2 N_2$	$C_2 H N_2$	Het-C(CN)=NH	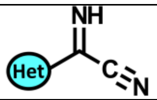
	Cyanoacetonitrile (electrophile) *Same mass as adduct from imidazole (see Nucleophile)	66	$C_3 H_2 N_2$	$C_3 H_3 N_2$	Het-C(CH_2-CN)=NH	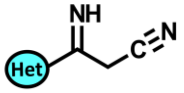
	HCN trimer (electrophile)	81	$C_3 H_3 N_3$	$C_3 H_4 N_3$	Het-C(CH(NH_2)-CN)=NH	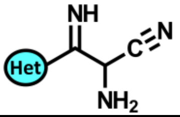
**Nucleophile**	Ammonia (nuc. substitution)	15	H N	H_2 N	Het-NH_2	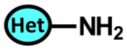
	Methylamine (nuc. substitution)<br><br>CH_2O + NH_3 *same mass/structure as degradation product of CH_2O + Urea + H_2O	29	C H_3 N	C H_4 N	Het-NH-CH_3<br><br>Het-CH_2-NH_2	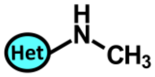 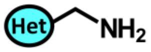
	Formamide (electrophile) *same mass as HCN + H_2O (nuc. addition) (see Carbonyl)	45	C H_3 O N	C H_4 O N	Het-(CHOH)-NH_2	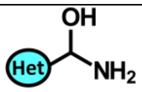
	Formamidine (electrophile)	44	C H_4 N_2	C H_5 N_2	Het-CH(NH_2)-NH_2	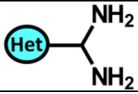
	Guanidine (nuc. substitution)	59	C H_5 N_3	C H_5 N_3	H-Het-NH-C(NH)-NH_2	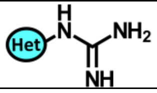
	Urea (nuc.substitution)	58	C H_2 O N_2	C H_3 O N_2	Het-NH-$CONH_2$	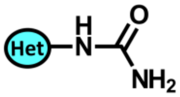
	CH_2O + Cyanamide *same mass as glycine (nuc. substitution) (see Carbonyl)	54	$C_2 H_2 N_2$	$C_2 H_3 N_2$	Het-CH_2-NH-CN	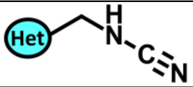
	CH_2O + Cyanamide + H_2O or CH_2O + Urea *same mass as glycine amide (nuc.substitution) (see Carbonyl)	72	$C_2 H_4$ O N_2	$C_2 H_5$ O N_2	Het-CH_2-NH-CO-NH_2	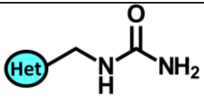
	CH_2O + Urea + H_2O *unstable, degrades to same mass as CH_2O + NH_3/Methylamine) (see Nucleophile)	73	$C_2 H_3 O_2 N$	$C_2 H_4 O_2 N$	Het-CH_2-NH-CO-OH *degradation product: Het-CH_2-NH_2	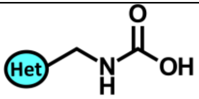

Side Chain Category	Hypothetical Reactants (nucleophile vs electrophile)	Δ MW (Da)	Δ Elemental Composition	Side Chain Composition	Possible Adduct Structures (Het =heterocycle)	
**Nucleophile**	Glycine nitrile (electrophile)	56	$C_2 H_4 N_2$	$C_2 H_5 N_2$	Het-C(CH_2-NH_2)=NH	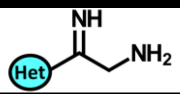
	Glycine condensation (electrophile) *Same mass as adduct from glycolonitrile + H_2O	57	$C_2 H_3$ O N	$C_2 H_4$ O N	Het-CH_2-$CONH_2$	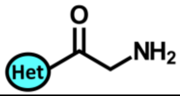
	Imidazole (nuc. substitution) *Same mass as adduct from Cyanoacetonitrile (see Nucleophile/Carbonyl)	66	$C_3 H_2 N_2$	$C_3 H_3 N_2$	Het-Imidazole	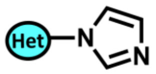
**Alcohol**	Formaldehyde (electrophile)	30	C H_2 O	C H_3 O	Het-CH_2-OH	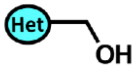
	Formaldehyde linked dimer (electrophile) ***can form larger oligomers**	(Het Mass) x 2 + 13			Het-CH_2-Het	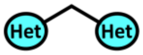
	Acetaldehyde (electrophile)	44	$C_2 H_4$ O	$C_2 H_5$ O	Het-CH(OH)-CH_3	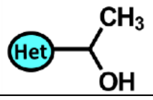
	Acetone (electrophile)	58	$C_3 H_6$ O	$C_3 H_7$ O	Het-C(CH_3)$_2$-OH	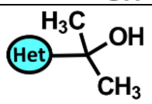
	Glycolaldehyde (electrophile)	60	$C_2 H_4 O_2$	$C_2 H_5 O_2$	Het-CH(OH)-CH_2-OH	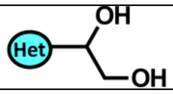
	3-C sugar (e.g., glyceraldehyde; electrophile)	90	$C_3 H_6 O_3$	$C_3 H_7 O_3$	Het-CHOH-CHOH-CH_2OH (i.e., Het-3-C sugar alcohol)	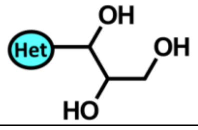
	4-C sugar (e.g., threose; electrophile)	120	$C_4 H_8 O_4$	$C_4 H_9 O_4$	Het-(CHOH-)$_3$-CH_2OH (i.e., Het-4-C sugar alcohol)	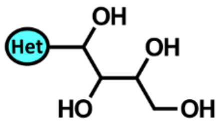
	5-C sugar (e.g., ribose; electrophile)	150	$C_5 H_{10} O_5$	$C_5 H_{11} O_5$	Het-(CHOH)$_4$-CH_2OH (i.e., Het-5-C sugar alcohol)	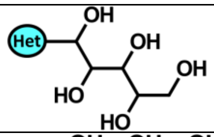
	6-C sugar (e.g., ribose; electrophile)	180	$C_6 H_{12} O_6$	$C_6 H_{13} O_6$	Het-(CHOH)$_5$-CH_2OH (i.e., Het-6-C sugar alcohol)	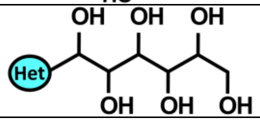
	3-C sugar alcohol condensation<br><br>Hydroxy acetone ***GNA subunit**	74	$C_3 H_6 O_2$	$C_3 H_7 O_2$	Het-CH_2-(CHOH)-CH_2OH /<br><br>Het- COH(CH_3)-CH_2OH	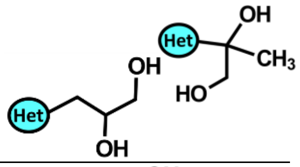
	4-C sugar alcohol condensation	104	$C_4 H_8 O_3$	$C_4 H_9 O_3$	Het-CH_2-(CHOH)$_2$-CH_2OH	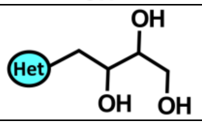

Side Chain Group Category	Hypothetical Reactants (nucleophile vs electrophile)	Δ MW (Da)	Δ Elemental Composition	Elemental Composition of side chain	Possible Adduct Structures (Het =heterocycle)	
**Alcohol**	5-C sugar alcohol condensation	134	$C_5 H_{10} O_4$	$C_5 H_{11} O_4$	Het-CH_2-$(CHOH)_3$-CH_2OH	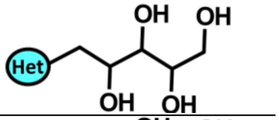
	6-C sugar alcohol condensation	164	$C_6 H_{12} O_5$	$C_6 H_{13} O_5$	Het-CH_2-$(CHOH)_4$-CH_2OH	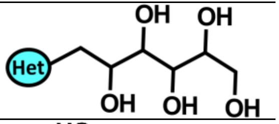
	Acrolein + 3 x Formaldehyde (Aldol Reaction)	118	$C_5 H_{10} O_3$	$C_5 H_{11} O_3$	Het-CH_2-$C(CH_2OH)_2$-CH_2OH	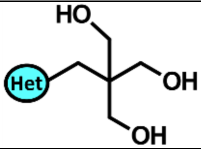
**Sugar**	Acrolein + CH_2O (Aldol Reaction) *Same mass as adduct formed from crotonitrile + 2 H_2O (see Carbonyl)	86	$C_4 H_6 O_2$	$C_4 H_7 O_2$	Het-CH_2-$CH(CH_2OH)$-CHO	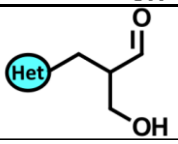
	Acrolein + 2 x CH_2O (Aldol Reaction)	116	$C_5 H_8 O_3$	$C_5 H_9 O_3$	Het-CH_2-$C(CH_2OH)_2$-CHO	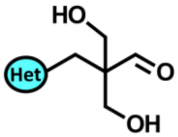
	4-C sugar (e.g., threose) condensation ***TNA nucleoside**	102	$C_4 H_6 O_3$	$C_4 H_7 O_3$	Het-4-C sugar	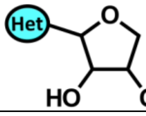
	5-C sugar (e.g., ribose) condensation ***RNA nucleoside**	132	$C_5 H_8 O_4$	$C_5 H_9 O_4$	Het-5-C sugar	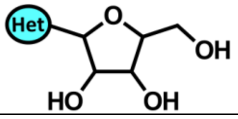
	6-C sugar (e.g., glucose) condensation	162	$C_6 H_{10} O_5$	$C_6 H_{11} O_5$	Het-6-C sugar	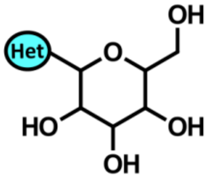
	Formic Acid + Methanediol *Same mass as adduct from glycolonitrile + 2 H_2O (see Carbonyl)	58	$C_2 H_2 O_2$	$C_2 H_3 O_2$	Het-CO-CH_2OH	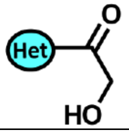
	Formic Acid + 2-Carbon sugar	88	$C_3 H_4 O_3$	$C_3 H_5 O_3$	Het-CO-(CHOH)-CH_2OH	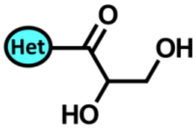
	Formic Acid + 3-Carbon sugar or 4-carbon γ-lactone	118	$C_4 H_6 O_4$	$C_4 H_7 O_4$	Het-CO-$(CHOH)_2$-CH_2OH	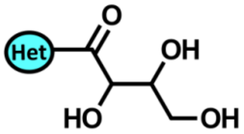

Side Chain Group Category	Hypothetical Reactants (nucleophile vs electrophile)	Δ MW (Da)	Δ Elemental Composition	Elemental Composition of side chain	Possible Adduct Structures (Het =heterocycle)	
**Sugar**	Formic Acid + 4-Carbon sugar or 5-carbon γ-lactone	148	$C_5 H_8 O_5$	$C_5 H_9 O_5$	Het-CO-(CHOH)$_3$-CH$_2$OH	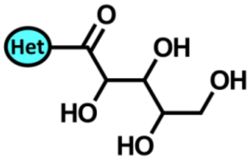
	Formic Acid + 5 Carbon sugar or 6-carbon γ-lactone	178	$C_6 H_{10} O_6$	$C_6 H_{11} O_6$	Het-CO-(CHOH)$_4$-CH$_2$OH	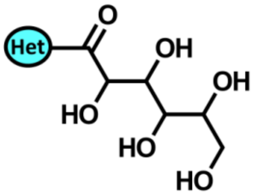

Table S3. Complete list of target adducts identified from spark mixtures generated under a reducing atmosphere. Reaction mixtures were either frozen immediately (-80 ℃) or incubated at 80 ℃ for 72 hours. Target adducts with the highest intensities in the respective mixture are bolded. The intensity of the same adduct identified in different reaction mixtures is provided to facilitate comparisons in replicate mixtures and those generated under a neutral atmosphere. **Acronyms**: A.U. (arbitrary units); ND (no adducts detected); N/A (not analyzed); AD (adducts detected); Red. Spk. (spark mixture generated under a reducing atmosphere of N_2, CO_2, H_2, and CH_4 gases); Net. Spk. (spark mixture generated under a neutral atmosphere of N_2 and CO_2 gases); Rep. (spark replicate); nuc. (nucleophilic). Dashes indicate adduct was not detected above thresholds. * designates notable adducts identified that were not targeted for analysis. † indicates that there was another target adduct with matching mass/formula (see Table S2), however only the most likely adduct is listed (based off the expected chemistry of the N-heterocycle); if the heterocycle can act as both a nucleophile and electrophile then both possible target adducts were provided. The total number of target adducts generated from different reactants is listed (i.e., unique adducts). Thus, adducts generated from the same reactant, but varying degrees of hydrolysis counted only once toward the total listed (e.g., adducts from glycolonitrile, glycolonitrile + H_2O, and glycolonitrile + 2 H_2O would count as one unique adduct); similarly, adducts generated from nucleophilic addition and substitution from the same reactant only counted once (e.g., H-Het-CN and Het-CN). Lastly, products in equilibrium with one another were also counted as a single unique adduct (e.g., adducts from formic acid (Het-CHO) and formic acid + NH_3 (Het-CHNH)—being able to readily convert to one another in the Miller-Urey mixture—count as a single unique adduct).

								Intensity (A.U.)				
**Pyridines**	**Heterocycle m/z (error ppm) Chemical Formula**	**Measured Mass**	**Elemental Composition**	**Side Chain Composition**	**Assigned Structure**	**Candidate Reactant**	**Mass Error (ppm)**	**Red. Spk.**	**Red. Spk. Rep.**	**Net. Spk .**	**Net. Spk. Rep.**	**# Unique Adducts**
1	**2-aminopyridine** m/z = 95.0602 (-2.3 ppm) $[C_5H_7N_2]^+$	**148.0868**	**$C_8H_{10}N_3$**	**C_3H_4N**	**Het-CH_2-CH_2-CN**	**Acrylonitrile**	**-0.7**	**1E+06**	**6E+03**	**1E+03**	**4E+03**	**8**
		179.0815	$C_9H_{11}O_2N_2$	$C_4H_5O_2$	Het-C(CH_3)=CH-COOH	Methylcyanoacetylene + 2 H_2O	-0.2	7E+05	2E+05	8E+03	8E+03	
		176.1181	$C_9H_{14}O_3N_3$	C_5H_8N	Het-CH(CH_2CH_3)-CH_2-CN	Ethyl acrylonitrile	5.2	3E+04	-	4E+05	-	
		134.0712	$C_7H_8N_3$	C_2H_2N	Het-CH_2-CN	Glycolonitrile	-0.7	6E+05	1E+04	4E+04	4E+04	
		162.1024	$C_9H_{12}N_3$	C_4H_6N	Het-CH(CH_3)-CH_2-CN / Het-CH_2-CH(CH_3)-CN	Crotonitrile / Methacrylonitrile	-0.8	5E+05	1E+03	2E+04	-	
		146.0712	$C_8H_8N_3$	C_3H_2N	Het-CH=CH-CN	Cyanoacetylene	-0.5	2E+05	2E+03	5E+03	1E+03	
		160.0868	$C_9H_{10}N_3$	C_4H_4N	Het-C(CH_3)=CH-CN	Methylcyanoacetylene	-0.6	1E+05	-	-	-	
		201.1133	$C_{11}H_{13}N_4$	$C_6H_7N_2$	Het-CH_2-Het	Formaldehyde linked dimer	1.3	2E+04	-	-	-	
		161.0824	$C_8H_9N_4$	$C_3H_3N_2$	Het-C(CH_2-CN)=NH	Cyanoacetonitrile (electrophile) †	1.6	1E+03	1E+03	-	-	
2	**4-aminopyridine** m/z = 95.0601 (-2.6 ppm) $[C_5H_7N_2]^+$	**152.0818**	**$C_7H_{10}ON_3$**	**C_2H_4ON**	**Het-CH_2-$CONH_2$**	**Glycolonitrile + H_2O †**	**-1.0**	**3E+05**	**N/A**	**N/A**	**N/A**	**6**
		134.0712	$C_7H_8N_3$	C_2H_2N	Het-CH_2-CN	Glycolonitrile	-0.8	8E+04	N/A	N/A	N/A	
		201.1129	$C_{11}H_{13}N_4$	$C_6H_7N_2$	Het-CH_2-Het	Formaldehyde linked dimer	-2.7	9E+03	N/A	N/A	N/A	
		162.1023	$C_9H_{12}N_3$	C_4H_6N	Het-CH(CH_3)-CH_2-CN / Het-CH_2-CH(CH_3)-CN	Crotonitrile / Methacrylonitrile	-1.5	5E+03	N/A	N/A	N/A	
		146.0713	$C_8H_8N_3$	C_3H_2N	Het-CH=CH-CN	Cyanoacetylene	-0.1	5E+03	N/A	N/A	N/A	
		160.0868	$C_9H_{10}N_3$	C_4H_4N	Het-C(CH_3)=CH-CN	Methylcyanoacetylene	-0.7	2E+03	N/A	N/A	N/A	
		161.0822	$C_8H_9N_4$	$C_3H_3N_2$	Het-C(CH_2-CN)=NH	Cyanoacetonitrile (electrophile) †	0.2	1E+03	N/A	N/A	N/A	

Table S3 Continued:

Pyridines	Heterocycle<br>m/z (error ppm)<br>Chemical Formula	Measured Mass	Elemental Composition	Side Chain Composition	Assigned Structure	Candidate Reactant	Mass Error (ppm)	Intensity (A. U.)				# Unique Adducts
								Red. Spk.	Red. Spk. Rep.	Net. Spk .	Net. Spk. Rep.	
**3**	**3-aminopyridine**<br>m/z = 95.0601 (-2.5 ppm)<br>$[C_5 H_7 N_2]^+$	**152.0817**	**$C_7 H_{10} O N_3$**	**$C_2 H_4 O N$**	**Het-CH_2-$CONH_2$**	**Glycolonitrile + H_2O †**	**-1.1**	**1E+07**	**2E+06**	**2E+03**	**2E+03**	**6**
		134.0712	$C_7 H_8 N_3$	$C_2 H_2 N$	Het-CH_2-CN	Glycolonitrile	-0.8	9E+05	4E+04	1E+03	1E+03	
		153.0657	$C_7 H_9 O_2 N_2$	$C_2 H_3 O_2$	Het-CH_2-COOH	Glycolonitrile + 2 H_2O †	-0.9	7E+05	3E+05	-	-	
		161.0821	$C_8 H_9 N_4$	$C_3 H_3 N_2$	Het-C(CH_2-CN)=NH	Cyanoacetonitrile (electrophile) †	-0.3	3E+04	-	-	-	
		148.0868	$C_8 H_{10} N_3$	$C_3 H_4 N$	Het-CH_2-CH_2-CN	Acrylonitrile	-1.1	2E+04	-	-	-	
		146.0711	$C_8 H_8 N_3$	$C_3 H_2$ N	Het-CH=CH-CN	Cyanoacetylene	-0.9	2E+04	-	-	-	
		160.0867	$C_9 H_{10} N_3$	$C_4 H_4 N$	Het-C(CH_3)=CH-CN	Methylcyanoacetylene	-1.1	2E+04	-	-	-	
		162.1026	$C_9 H_{12} N_3$	$C_4 H_6 N$	Het-CH(CH_3)-CH_2-CN /<br>Het-CH_2-CH(CH_3)-CN	Crotonitrile /<br>Methacrylonitrile	-1.7	8E+03	8E+03	-	-	
**4**	**3-hydroxymethylpyridine**<br>m/z = 110.0599 (-1.6 ppm)<br>$[C_6 H_8 O N]^+$	**153.0658**	**$C_7 H_9 O_2 N_2$**	**$C H_2 O N$**	**Het-$CONH_2$**	**HCN + H_2O (nuc. substitution)**	**-0.6**	**4E+05**	**2E+05**	**N/A**	**N/A**	**2**
		231.1121	$C_{13} H_{15} O_2 N_2$	$C_7 H_8 O N$	Het-CH_2-Het	Formaldehyde linked dimer	-3.2	3E+03	-	N/A	N/A	
**5**	**4-hydroxymethylpyridine**<br>m/z = 110.0599 (-1.5 ppm)<br>$[C_6 H_8 O N]^+$	No Adducts Detected							N/A	N/A	N/A	**0**
**6**	**2-hydroxymethylpyridine**<br>m/z = 110.0599 (-1.2 ppm)<br>$[C_6 H_8 O N]^+$	No Adducts Detected							N/A	N/A	N/A	**0**
**7**	**2-pyridinecarboxylic acid**<br>m/z = 124.0390 (-2.5 ppm)<br>$[C_6 H_6 O_2 N]^+$	No Adducts Detected							N/A	ND	AD	**0**
**8**	**3-pyridinecarboxylic acid**<br>m/z = 124.0391 (-1.9 ppm)<br>$[C_6 H_6 O_2 N]^+$	No Adducts Detected							N/A	N/A	AD	**0**
**9**	**4-pyridinecarboxylic acid**<br>m/z = 124.0390 (-2.7 ppm)<br>$[C_6 H_6 O_2 N]^+$	**182.0448**	**$C_8 H_8 O_4 N$**	**$C_2 H_3 O_2$**	**Het-CH_2-COOH**	**Glycolonitrile + 2 H_2O †**	**0.4**	**3E+03**	**N/A**	**N/A**	-	**1**
**10**	**4-cyanopyridine**<br>m/z = 105.0446 (-1.5 ppm)<br>$[C_6 H_5 N_2]^+$	No Adducts Detected							N/A	N/A	N/A	**0**
**11**	**3-cyanopyridine**<br>m/z = 105.0446 (-1.5 ppm)<br>$[C_6 H_5 N_2]^+$	No Adducts Detected							N/A	N/A	N/A	**0**
**12**	**2-cyanopyridine**<br>m/z = 105.0446 (-1.2 ppm)<br>$[C_6 H_5 N_2]^+$	No Adducts Detected							N/A	N/A	N/A	**0**

Table S3 Continued:

Pyrimidines	Heterocycle m/z (error ppm) Chemical Formula	Measured Mass	Elemental Composition	Side Chain Composition	Assigned Structure	Candidate Reactant	Mass Error (ppm)	Intensity (A.U.) Red. Spk.	Red. Spk. Rep.	Net. Spk .	Net. Spk. Rep.	# Unique Adducts
**13**	**2-aminopyrimidine** m/z = 96.0554 (-2.2 ppm) $[C_4 H_6 N_3]^+$	No Adducts Detected							N/A	N/A	N/A	**0**
**14**	**4-aminopyrimidine** m/z = 96.0554 (-2.6 ppm) $[C_4 H_6 N_3]^+$	No Adducts Detected							N/A	N/A	N/A	**0**
**15**	**2,4-diaminopyrimidine** m/z = 111.0671 (5.3 ppm) $[C_4 H_7 N_4]^+$	**165.0777**	**$C_7 H_9 O N_4$**	**$C_3 H_3 O$**	**Het-CH=CH=CHO**	**Propiolaldehyde**	**7.0**	**N/A**	**5E+05**	**-**	**-**	**7**
		233.1267	$C_9 H_{13} N_8$	$C_5 H_7 N_4$	Het-CH_2-Het	Formaldehyde linked dimer	3.9	N/A	3E+05	-	-	
		183.0874	$C_7 H_{11} O_2 N_4$	$C_3 H_5 O_2$	Het-CH_2-CH_2-COOH	Acrylonitrile + 2 H_2O	-1.2	N/A	4E+04	-	-	
		169.0718	$C_6 H_9 O_2 N_4$	$C_2 H_3 O_2$	Het-CH_2-COOH	Glycolonitrile + 2 H_2O †	-1.4	N/A	2E+04	-	-	
		179.0919	$C_8 H_{11} O N_4$	$C_4 H_5 O$	Het-C(CH_3)=CH-CHO	Methylpropiolaldehyde	-4.5	N/A	2E+04	-	-	
		150.0778	$C_6 H_8 N_5$	$C_2 H_2 N$	Het-CH_2-CN	Glycolonitrile	7.0	N/A	1E+04	-	-	
		194.1030	$C_8 H_{12} O N_5$	$C_4 H_6 O N$	Het-C(CH_3)=CH-$CONH_2$	Methylcyanoacetylene + H_2O	-3.2	N/A	1E+04	-	-	
		168.0878	$C_6 H_{10} O N_5$	$C_2 H_4 O N$	Het-CH_2-$CONH_2$	Glycolonitrile + H_2O †	6.7	N/A	8E+03	1E+04	-	
		243.1081	$C_9 H_{15} O_4 N_4$	$C_5 H_9 O_4$	Het-5-C sugar	5-C sugar condensation	-6.4	N/A	7E+03	1E+03	1E+03	
**16**	**2,4,6-triaminopyrimidine** m/z = 126.0784 (8.1 ppm) $[C_4 H_8 N_5]^+$	**209.1163**	**$C_8 H_{13} O N_6$**	**$C_4 H_6 O N$**	**Het-C(CH_3)=CH-$CONH_2$**	**Methylcyanoacetylene + H_2O**	**-3.5**	**N/A**	**1E+07**	**-**	**-**	**9**
		165.0894	$C_6 H_9 N_6$	$C_2 H_2 N$	Het-CH_2-CN	Glycolonitrile	6.7	N/A	4E+06	9E+04	4E+04	
		183.1003	$C_6 H_{11} O N_6$	$C_2 H_4 O N$	Het-CH_2-$CONH_2$	Glycolonitrile + H_2O †	7.8	N/A	2E+06	-	-	
		191.1052	$C_8 H_{11} N_6$	$C_4 H_4 N$	Het-C(CH_3)=CH-CN	Methylcyanoacetylene	6.4	N/A	1E+06	1E+05	-	
		177.0894	$C_7 H_9 N_6$	$C_3 H_2 N$	Het-CH=CH-CN	Cyanoacetylene	6.3	N/A	5E+05	-	-	
		184.0838	$C_6 H_{10} O_2 N_5$	$C_2 H_3 O_2$	Het-CH_2-COOH	Glycolonitrile + 2 H_2O †	5.1	N/A	2E+05	-	-	
		154.0727	$C_5 H_8 O N_5$	C H O	Het-CHO	Formic Acid	2.2	N/A	3E+04	2E+04	5E+04	
		210.0980	$C_8 H_{12} O_2 N_5$	$C_4 H_5 O_2$	Het-C(CH_3)=CH-COOH	Methylcyanoacetylene + 2 H_2O	-2.9	N/A	3E+04	-	-	
		180.0883	$C_7 H_{10} O N_5$	$C_3 H_3 O$	Het-CH=CH=CHO	Propiolaldehyde	2.0	N/A	3E+04	-	-	
		244.1041	$C_8 H_{14} O_4 N_5$	$C_4 H_7 O_4$	Het-CO-$(CHOH)_2$-CH_2OH	Formic Acid + 3-Carbon sugar / 4-carbon γ-lactone	0.4	N/A	1E+04	-	-	
		196.0833	$C_7 H_{10} O_2 N_5$	$C_3 H_3 O_2$	Het-CH=CH-COOH	Cyanoacetylene + 2 H_2O	2.2	N/A	3E+03	-	-	
		182.1048	$C_7 H_{12} O N_5$	$C_3 H_5 O$	Het-CH_2-CH_2-CHO	Acrolein	6.4	N/A	3E+03	2E+03	-	
		212.1135	$C_8 H_{14} O_2 N_5$	$C_4 H_7 O_2$	Het-CH(CH_3)-CH_2-COOH / Het-CH_2-CH(CH_3)-COOH	Crotonitrile + 2 H_2O / Methacrylonitrile + 2 H_2O †	-3.4	N/A	3E+03	1E+04	1E+04	
		194.1032	$C_8 H_{12} O N_5$	$C_4 H_5 O$	Het-C(CH_3)=CH-CHO	Methylpropiolaldehyde	-2.5	N/A	3E+03	-	-	

Table S3 Continued:

Pyrimidines	Heterocycle m/z (error ppm) Chemical Formula	Measured Mass	Elemental Composition	Side Chain Composition	Assigned Structure	Candidate Reactant	Mass Error (ppm)	Intensity (A.U.) Red. Spk.	Red. Spk. Rep.	Net. Spk .	Net. Spk. Rep.	# Unique Adducts
**17**	**Cytosine**<br>m/z = 112.0502 (-3.4 ppm)<br>$[C_4 H_6 O N_3]^+$	**276.1199**	**$C_{10} H_{18} O_6 N_3$**	**$C_6 H_{13} O_5$**	**Het-CH_2-$(CHOH)_4$-CH_2OH**	**6-C sugar alcohol condensation**	**3.4**	**-**	**1E+03**	**-**	**N/A**	**1**
**18**	**Isocytosine**<br>m/z = 112.0503 (-2.1 ppm)<br>$[C_4 H_6 O N_3]^+$	**166.0611**	**$C_7 H_8 O_2 N_3$**	**$C_3 H_3 O$**	**Het-CH=CH=CHO**	**Propiolaldehyde**	**0.0**	**9E+05**	**N/A**	**-**	**N/A**	**2**
		177.0771	$C_8 H_9 O N_4$	$C_4 H_4 N$	Het-$C(CH_3)$=CH-CN	Methylcyanoacetylene	-0.2	3E+04	N/A	-	N/A	
**19**	**4-hydroxy pyrimidine**<br>m/z = 97.0394 (-2.6 ppm)<br>$[C_4 H_5 O N_2]^+$	**169.0607**	**$C_7 H_9 O_3 N_2$**	**$C_3 H_5 O_2$**	**Het-CH_2-CH_2-COOH**	**Acrylonitrile + 2 H_2O**	**-0.5**	**8E+05**	**-**	**N/A**	**N/A**	**1**
**20**	**2-hydroxy pyrimidine**<br>m/z = 97.0393 (-3.0 ppm)<br>$[C_4 H_5 O N_2]^+$	**144.0765**	**$C_5 H_{10} O_2 N_3$**	**$C H_5 O N$**	**H-Het-NH-CH_2-OH**	**Hydroxymethylamine***	**-1.8**	**3E+06**	**1E+06**	**-**	**-**	**4**
		169.0604	$C_7 H_9 O_3 N_2$	$C_3 H_5 O_2$	Het-CH_2-CH_2-COOH	Acrylonitrile + 2 H_2O	-2.1	9E+05	3E+04	3E+04	3E+04	
		182.0921	$C_8 H_{12} O_2 N_3$	$C_4 H_8 O N$	Het-$CH(CH_3)$-CH_2-$CONH_2$ /<br>Het-CH_2-$CH(CH_3)$-$CONH_2$	Crotonitrile + H_2O /<br>Methacrylonitrile + H_2O	-1.7	7E+05	-	-	8E+03	
		162.0659	$C_8 H_8 O N_3$	$C_4 H_4 N$	Het-$C(CH_3)$=CH-CN	Methylcyanoacetylene	-1.9	4E+04	1E+04	8E+03	-	
		136.0504	$C_6 H_6 O N_3$	$C_2 H_2 N$	Het-CH_2-CN	Glycolonitrile	0.5	2E+04	-	2E+04	4E+03	
**21**	**Uracil**<br>m/z = 113.0343 (-2.3 ppm)<br>$[C_4 H_5 O_2 N_2]^+$	**185.0556**	**$C_7 H_9 O_4 N_2$**	**$C_3 H_5 O_2$**	**Het-CH_2-CH_2-COOH**	**Acrylonitrile + 2 H_2O**	**0.3**	**3E+05**	**1E+04**	**2E+03**	**-**	**7**
		170.0559	$C_6 H_8 O_3 N_3$	$C_2 H_4 O N$	Het-CH_2-$CONH_2$	5HMU + HCN condensation + H_2O /<br>Glycolonitrile + H_2O †	-0.6	8E+04	-	5E+03	-	
		237.0619	$C_9 H_9 O_4 N_4$	$C_5 H_5 O_2 N_2$	Het-CH_2-Het	Formaldehyde linked dimer	0.3	7E+04	1E+04	-	-	
		167.0450	$C_7 H_7 O_3 N_2$	$C_3 H_3 O$	Het-CH=CH=CHO	Propiolaldehyde	-0.1	3E+04	-	-	-	
		181.0607	$C_8 H_9 O_3 N_2$	$C_4 H_5 O$	Het-$C(CH_3)$=CH-CHO	Methylpropiolaldehyde	-0.2	1E+04	-	-	-	
		187.0714	$C_7 H_{11} O_4 N_2$	$C_3 H_7 O_2$	Het-CH_2-(CHOH)-CH_2OH	Hydroxy acetone	0.3	8E+03	-	-	-	
		185.0668	$C_6 H_9 O_3 N_4$	$C_2 H_5 O N_2$	Het-CH_2-NH-CO-NH_2	Formaldehyde + Urea †	-0.4	3E+03	5E+03	-	-	
**22**	**Barbituric Acid**<br>mass = 129 g/mol (ND)<br>$[C_4 H_5 O_3 N_2]^+$	**217.0569**	**$C_6 H_9 O_5 N_4$**	**$C_2 H_5 O_2 N_2$**	**Het-NH-$CH(NH_2)$-COOH**	**Amino-glycine (nuc. substitution)**	**0.6**	**N/A**	**2E+03**	**-**	**N/A**	**1**
**23**	**Thymine**<br>m/z = 127.0500 (-1.7 ppm)<br>$[C_5 H_7 O_2 N_2]^+$	**199.0714**	**$C_8 H_{11} O_4 N_2$**	**$C_3 H_5 O_2$**	**Het-CH_2-CH_2-COOH**	**Acrylonitrile + 2 H_2O**	**0.2**	**2E+05**	**8E+03**	**3E+03**	**-**	**3**
		184.0716	$C_7 H_{10} O_3 N_3$	$C_2 H_4 O N$	Het-CH_2-$CONH_2$	Glycolonitrile + H_2O †	-0.3	1E+05	-	5E+04	-	
		181.0608	$C_8 H_9 O_3 N_2$	$C_3 H_3 O$	Het-CH=CH=CHO	Propiolaldehyde	0.1	2E+04	-	-	-	

Table S3 Continued:

Pyrimidines	Heterocycle m/z (error ppm) Chemical Formula	Measured Mass	Elemental Composition	Side Chain Composition	Assigned Structure	Candidate Reactant	Mass Error (ppm)	Intensity (A. U.)				# Unique Adducts
								Red. Spk.	Red. Spk. Rep.	Net. Spk .	Net. Spk. Rep.	
**24**	**5-hydroxymethyluracil** m/z = 143.0455 (2.3 ppm) $[C_5 H_7 O_3 N_2]^+$	**170.0559**	**$C_6 H_8 O_3 N_3$**	**$C H_2 O N$**	**U-CH_2-$CONH_2$**	**(-H_2O) HCN + H_2O**	**-0.8**	**5E+06**	**1E+07**	**8E+04**	**1E+07**	**13**
		153.0293	$C_6 H_5 O_3 N_2$	C H O	Het-CHO	Formic Acid	-0.8	2E+06	8E+06	-	8E+06	
		152.0453	$C_6 H_6 O_2 N_3$	C N	U-CH_2-CN	(-H_2O) HCN	-0.7	2E+05	1E+05	-	1E+05	
		193.0722	$C_8 H_9 O_2 N_4$	$C_3 H_3 N_2$	U-CH_2-imidazole	(-H_2O) Imidazole (nucleophile, condensation)	1.3	2E+05	-	5E+03	-	
		200.0666	$C_7 H_{10} O_4 N_3$	$C_2 H_4 O N$	U-CH_2-NH-CH_2-COOH / Het-CH_2-$CONH_2$	(-H_2O) Glycine / Glycolonitrile + H_2O	0.0	7E+04	1E+04	-	1E+04	
		224.0666	$C_9 H_{10} O_4 N_3$	$C_4 H_4 O_2 N$	U(CH_2CN)-CH_2-CH_2-COOH / U(CH_2COOH)-$(CH_2)_2$-CN	(-H_2O) HCN + Acrylonitrile + 2 H_2O	-0.1	5E+04	3E+04	-	3E+04	
		223.0827	$C_9 H_{11} O_3 N_4$	$C_4 H_5 O N$	U(CH_2CN)-CH_2-CH_2-$CONH_2$ / U(CH_2CONH_2)-$(CH_2)_2$-CN	(-H_2O) CN + H_2O + Acrylonitrile	-0.1	5E+04	4E+03	5E+04	4E+03	
		212.0666	$C_8 H_{10} O_4 N_3$	$C_3 H_4 O N$	Het-CH=CH-$CONH_2$	Cyanoacetylene + H_2O	0.1	4E+04	3E+03	-	3E+03	
		221.0668	$C_9 H_9 O_3 N_4$	$C_4 H_3 O N_2$	Het(CH_2CN)-CH=CH-$CONH_2$	(-H_2O) HCN + Cyanoacetylene + H_2O	-0.3	3E+04	5E+03		5E+03	
		236.0665	$C_{10} H_{10} O_4 N_3$	$C_5 H_4 O_2 N$	U-(CH_2CN)-C(CH_3)=CH-COOH / U-(CH_2COOH)-C(CH_3)=CH-CN	(-O) HCN + Methylcyanoacetylene + 2 H_2O	2.4	3E+04	2E+03	-	2E+03	
		235.0826	$C_{10} H_{11} O_3 N_4$	$C_5 H_5 O N_2$	U(CH_2CN)-C(CH_3)=CH-$CONH_2$	(-H_2O) HCN + Methylcyanoacetylene + H_2O	0.0	3E+04	2E+03	-	2E+03	
		227.0772	$C_8 H_{11} O_4 N_4$	$C_3 H_5 O_2 N_2$	U(CH_2CONH_2)-CH_2-$CONH_2$	(-H_2O) HCN + Glycolonitrile + 2 H_2O	-1.1	2E+04	-	-	-	
		238.0821	$C_{10} H_{12} O_4 N_3$	$C_5 H_6 O_2 N$	U(CH_2CN)-C(CH_3)-CH_2-COOH / U(CH_2COOH)-C(CH_3)-CH_2-CN Methacrylonitrile not shown	(-H_2O) HCN + Crotonitrile + 2 H_2O / Methacrylonitrile + 2 H_2O	-0.5	2E+04	-	-	-	
		241.0931	$C_9 H_{13} O_4 N_4$	$C_4 H_7 O_2 N_2$	U(CH_2CONH_2)-CH_2-CH_2-$CONH_2$	(-H_2O) HCN + Acrylonitrile + 2 H_2O	-0.2	2E+04	1E+03	-	1E+03	
		209.0669	$C_8 H_9 O_3 N_4$	$C_3 H_3 O N_2$	Het-(CH_2CN)-CH_2-$CONH_2$	(-H_2O) HCN+ Glycolonitrile + H_2O	-0.2	1E+04	4E+04	6E+04	4E+04	
		226.0823	$C_9 H_{12} O_4 N_3$	$C_4 H_6 O N$	Het-C(CH_3)=CH-$CONH_2$	Methylcyanoacetylene + H_2O	0.5	1E+04	-	-	-	
		206.0560	$C_9 H_8 O_3 N_3$	$C_4 H_2 O N$	U(CH_2CN)-CH=CH=CHO	(-H_2O) HCN + Propiolaldehyde	0.4	1E+04	-	-	-	
		323.1099	$C_{11} H_{19} O_9 N_2$	$C_6 H_{13} O_6$	Het-$(CHOH)_5$-CH_2OH	6-C sugar (electrophile)	4.3	3E+03	-	-	N/A	
		242.0776	$C_9 H_{12} O_5 N_3$	$C_4 H_6 O_3 N$	U(CH_2CONH_2)-$(CH_2)_2$COOH/ U(CH_2COOH)-$(CH_2)_2CONH_2$	(-H_2O) HCN + Acrylonitrile + 3 H_2O	1.8	2E+03	-	-	-	
		185.0669	$C_6 H_9 O_3 N_4$	$C H_3 O N_2$	U-CH_2-NH-CO-NH_2	- H_2O Urea (nucleophile, condensation)	-0.7	2E+03	-	-	-	
		207.0512	$C_7 H_7 O_3 N_4$	$C_2 H N_2$	Het-C(CN)=NH	Cyanogen	-0.1	1E+03	2E+05	-	2E+05	
**25**	**Orotic Acid** m/z = 157 (in pos. mode) (ND in pos. or neg. mode) $[C_5 H_5 N_2 O_4]^+$	No Adducts Detected							N/A	N/A	N/A	**0**

Table S3 Continued:

								Intensity (A.U.)				
**Triazines**	**Heterocycle m/z (error ppm) Chemical Formula**	**Measured Mass**	**Elemental Composition**	**Side Chain Composition**	**Assigned Structure**	**Candidate Reactant**	**Mass Error (ppm)**	**Red. Spk.**	**Red. Spk. Rep.**	**Net. Spk .**	**Net. Spk. Rep.**	**# Unique Adducts**
**26**	**3-amino-1,2,4-triazine** m/z = 97.0506 (-2.4 ppm) $[C_3 H_5 N_4]^+$	191.0787	$C_6 H_7 N_8$	$C_3 H_3 N_4$	Het-Het	CN induced covalent dimerization*	-0.8	1E+07	1E+06	8E+06	-	**19**
		**140.0565**	**$C_4 H_6 O N_5$**	**$C H_2 O N$**	**Het-CONH$_2$**	**HCN + H_2O (nuc. substitution)**	**-1.2**	**7E+06**	**5E+06**	**1E+05**	**3E+04**	
		112.0616	$C_3 H_6 N_5$	$H_2 N$	Het-NH$_2$	Ammonia	-1.4	8E+05	2E+06	1E+05	6E+04	
		136.0617	$C_5 H_6 N_5$	$C_2 H_2 N$	Het-CH$_2$-CN	Glycolonitrile	-1.0	6E+05	2E+04	4E+05	1E+05	
		178.0835	$C_6 H_8 N_7$	$C_3 H_4 N_3$	Het-C(CH(NH$_2$)-CN)=NH	HCN trimer	-0.4	5E+05	2E+05	2E+03	-	
		127.0613	$C_4 H_7 O N_4$	$C H_3 O$	Het-CH$_2$-OH	Formaldehyde	-1.2	3E+05	2E+05	3E+04	7E+03	
		167.0562	$C_6 H_7 O_2 N_4$	$C_3 H_3 O_2$	Het-CH=CH-COOH	Cyanoacetylene + 2 H_2O	-0.9	2E+05	1E+05	-	-	
		205.0945	$C_7 H_9 N_8$	$C_4 H_5 N_4$	Het-CH$_2$-Het	Formaldehyde linked dimer	0.2	2E+05	3E+04	3E+03	-	
		151.0613	$C_6 H_7 O N_4$	$C_3 H_3 O$	Het-CH=CH=CHO	Propiolaldehyde	-2.2	1E+05	6E+04	4E+03	-	
		163.0728	$C_6 H_7 N_6$	$C_3 H_3 N_2$	Het-C(CH$_2$-CN)=NH / Het-imidazole	Cyanoacetonitrile (electrophile) / Imidazole (nuc. substitution)	-0.6	9E+04	2E+05	-	-	
		169.0832	$C_5 H_9 O N_6$	$C_2 H_5 O N_2$	Het-NH-CH$_2$-CONH$_2$ / Het-CH$_2$-NH-CO-NH$_2$	Glycine amide (nuc. substitution) / Formaldehyde + Urea	-0.2	9E+04	3E+04	-	-	
		151.0726	$C_5 H_7 N_6$	$C_2 H_3 N_2$	Het-NH-CH$_2$-CN / Het-CH$_2$-NH-CN	Glycine nitrile (nuc. substitution) / Formaldehyde + Cyanamide	-0.1	6E+04	2E+05	-	-	
		124.0616	$C_4 H_6 N_5$	$C N$ / $C H_2 N$	H-Het-CN / Het-CH=NH	HCN (nuc. addition) / Formic Acid + NH_3	-0.8	6E+04	9E+03	1E+03	-	
		154.0722	$C_5 H_8 O N_5$	$C_2 H_4 O N$	Het-CH$_2$-CONH$_2$	Glycolonitrile + H_2O †	-1.1	5E+04	2E+04	1E+04	6E+03	
		126.0773	$C_4 H_8 N_5$	$C H_4 N$	Het-NH-CH$_3$ / Het-CH$_2$-NH$_2$	Methylamine / Formaldehyde + NH_3 or Degradation of *m/z* 170.0672	-1.4	5E+04	4E+04	-	-	
		170.0672	$C_5 H_8 O_2 N_5$	$C_2 H_4 O_2 N$	Het-NH-CH$_2$-COOH / Het-CH$_2$-NH-CO-OH	Glycine (nuc substitution) / Formaldehyde + Urea + H_2O	-0.5	4E+04	8E+03	-	-	
		122.0461	$C_4 H_4 N_5$	$C N$	Het-CN	HCN (nuc. substitution)	-0.7	3E+04	1E+03	2E+03	-	
		141.0406	$C_4 H_5 O_2 N_4$	$C H O_2$	Het-COOH	HCN + 2 H_2O (nuc. substitution)	-0.8	3E+04	2E+04	8E+03	8E+03	
		155.0675	$C_4 H_7 O N_6$	$C H_3 O N_2$	Het-NH-CONH$_2$	Urea (nuc. substitution)	-0.2	3E+04	5E+05	2E+04	3E+03	
		154.0836	$C_4 H_8 N_7$	$C H_3 O N_2$	Het-NH-CNH-NH$_2$	Guanidine (nuc. substitution)	0.1	2E+04	2E+04	-	-	
		168.0879	$C_6 H_{10} O N_5$	$C_3 H_6 O N$	Het-CH$_2$-CH$_2$-CONH$_2$	Acrylonitrile + H_2O	-0.4	2E+04	1E+03	1E+03	1E+03	
		155.0561	$C_5 H_7 O_2 N_4$	$C_2 H_3 O_2$	Het-CH$_2$-COOH	Glycolonitrile + 2 H_2O †	-1.4	2E+04	2E+03	2E+03	3E+03	
		143.0562	$C_4 H_7 O_2 N_4$	$C H_2 O_2$	H-Het-COOH	HCN + 2 H_2O (nuc. addition)	-0.9	2E+04	-	-	-	
		138.0772	$C_5 H_8 N_5$	$C_2 H_4 N$	Het-C(CH$_3$)=NH	Acetic Acid + NH_3	-1.4	1E+04	7E+03	7E+03	5E+03	
		125.0456	$C_4 H_5 O N_4$	$C H O$	Het-CHO	Formic Acid	-1.1	1E+04	-	-	-	
		277.1154	$C_9 H_{17} O_6 N_4$	$C_6 H_{13} O_6$	Het-(CHOH)$_5$-CH$_2$OH	6-C sugar (electrophile)	4.2	-	1E+04	-	-	
		184.0828	$C_6 H_{10} O_2 N_5$	$C_3 H_6 O_2 N$	Het-NH-CH(CH$_3$)-COOH	Alanine (nuc. substitution)	-0.3	9E+03	-	-	-	
		162.0775	$C_7 H_8 N_5$	$C_4 H_4 N$	Het-C(CH$_3$)=CH-CN	Methylcyanoacetylene	0.6	6E+03	6E+03	6E+03	-	
		185.0665	$C_6 H_9 O_3 N_4$	$C_3 H_5 O_3$	Het-CO-(CHOH)-CH$_2$OH	Formic Acid + 2-Carbon sugar	-2.4	2E+03	-	6E+03	-	
		245.0890	$C_8 H_{13} O_5 N_4$	$C_4 H_7 O_4$	Het-CO-(CHOH)$_2$-CH$_2$OH	Formic Acid + 3-Carbon sugar / 4-carbon γ-lactone	3.9	1E+03	-	-	-	

Table S3 Continued:

Triazines	Heterocycle m/z (error ppm) Chemical Formula	Measured Mass	Elemental Composition	Side Chain Composition	Assigned Structure	Candidate Reactant	Mass Error (ppm)	Intensity (A.U.) Red. Spk.	Red. Spk. Rep.	Net. Spk .	Net. Spk. Rep.	# Unique Adducts
**27**	**Guanazole** m/z = 100.0615 (-3.0 ppm) $[C_2 H_6 N_5]^+$	**157.0831**	**$C_4 H_9 O N_6$**	**$C_2 H_4 O N$**	**Het-CH_2-$CONH_2$**	**Glycolonitrile + H_2O †**	**-0.9**	**3E+06**	**3E+05**	**-**	**-**	**7**
		171.0988	$C_5 H_{11} O N_6$	$C_3 H_6 O N$	Het-CH_2-CH_2-$CONH_2$	Acrylonitrile + H_2O	-0.6	6E+05	9E+04	-	-	
		183.0988	$C_6 H_{11} O N_6$	$C_4 H_6 O N$	Het-C(CH_3)=CH-$CONH_2$	Methylcyanoacetylene + H2O	2.0	4E+05	1E+05	-	-	
		185.1145	$C_6 H_{13} O N_6$	$C_4 H_8 O N$	Het-CH(CH_3)-CH_2-$CONH_2$ / Het-CH_2-CH(CH_3)-$CONH_2$	Crotonitrile + H_2O / Methacrylonitrile + H_2O	-0.3	2E+05	4E+04	-	-	
		158.0671	$C_4 H_8 O_2 N_5$	$C_2 H_3 O_2$	Het-CH_2-COOH	Glycolonitrile + 2 H_2O †	-0.7	2E+05	4E+04	-	-	
		156.0879	$C_5 H_{10} O N_5$	$C_3 H_5 O$	Het-CH_2-CH_2-CHO	Acrolein	-0.7	8E+04	5E+04	-	-	
		168.0879	$C_7 H_{10} O_2 N_5$	$C_4 H_5 O$	Het-C(CH_3)=CH-CHO	Methylpropiolaldehyde	0.6	5E+04	3E+03	-	-	
		165.0885	$C_6 H_9 N_6$	$C_4 H_4 N$	Het-C(CH_3)=CH-CN	Methylcyanoacetylene	-0.6	4E+04	1E+04	-	-	
		169.0831	$C_5 H_9 O N_6$	$C_3 H_4 O N$	Het-CH=CH-$CONH_2$	Cyanoacetylene + H_2O	-0.6	3E+04	8E+03	-	-	
		153.0883	$C_5 H_9 N_6$	$C_3 H_4 N$	Het-CH_2-CH_2-CN	Acrylonitrile	-0.3	2E+04	7E+03	-	-	
		167.1042	$C_6 H_{11} N_6$	$C_4 H_6 N$	Het-CH(CH_3)-CH_2-CN / Het-CH_2-CH(CH_3)-CN	Crotonitrile / Methacrylonitrile	1.6	4E+03	5E+03	-	-	
		170.0673	$C_5 H_8 O_2 N_5$	$C_3 H_3 O_2$	Het-CH=CH-COOH	Cyanoacetylene + 2 H_2O	0.3	1E+03	-	-	-	
**28**	**Melamine** m/z = 127.0724 (-2.0 ppm) $[C_3 H_7 N_6]^+$	**184.0940**	**$C_5 H_{10} O N_7$**	**$C_2 H_4 O N$**	**Het-CH_2-$CONH_2$**	**Glycolonitrile + H_2O †**	**-0.5**	**2E+04**	**N/A**	**N/A**	-	**1**
**29**	**Ammeline** m/z = 128.0565 (-1.8 ppm) $[C_3 H_6 O N_5]^+$	**182.0673**	**$C_6 H_8 O_2 N_5$**	$C_3 H_3 O$	Het-CH=CH=CHO	**Propiolaldehyde**	**0.1**	**4E+04**	**N/A**	**N/A**	**N/A**	**1**
**30**	**Ammelide** m/z = 127.0264 (1.8 ppm) $[C_3 H_3 O_2 N_4]^-$	**169.0365**	**$C_5 H_5 O_3 N_4$**	**$C_2 H_3 O$**	**Het-CO-CH_3**	**Acetic Acid**	**-1.1**	**8E+03**	**N/A**	**N/A**	**N/A**	**8**
		178.0363	$C_6 H_4 O_2 N_5$	$C_3 H_2 N$	Het-CH=CH-CN	Cyanoacetylene	-3.9	2E+03	N/A	N/A	N/A	
		166.0369	$C_5 H_4 O_2 N_5$	$C_2 H_2 N$	Het-CH_2-CN	Glycolonitrile	-0.7	2E+03	N/A	N/A	N/A	
		192.0520	$C_7 H_6 O_2 N_5$	$C_4 H_4 N$	Het-C(CH_3)=CH-CN	Methylcyanoacetylene	-3.8	2E+03	N/A	N/A	N/A	
		195.0520	$C_7 H_7 O_3 N_4$	$C_4 H_5 O$	Het-C(CH_3)=CH-CHO	Methylpropiolaldehyde	-1.6	2E+03	N/A	N/A	N/A	
		168.0524	$C_5 H_6 O_2 N_5$	$C_2 H_4 N$	Het-C(CH_3)=NH	Acetic Acid + NH_3	-1.7	1E+03	N/A	N/A	N/A	
		183.0514	$C_6 H_7 O_3 N_4$	$C_3 H_5 O$	Het-CH_2-CH_2-CHO	Acrolein	-5.3	1E+03	N/A	N/A	N/A	
		227.0787	$C_8 H_{11} O_4 N_4$	$C_5 H_9 O_2$	Het-CH(CH_2CH_3)-CH_2-COOH	Ethyl acrylonitrile + 2 H_2O	0.7	1E+03				
		155.0210	$C_4 H_3 O_3 N_4$	C H O	Het-CHO	Formic Acid	-0.6	1E+03	N/A	N/A	N/A	
**31**	**s-Triazine** m/z = 82 in positive mode (ND in pos. or neg. mode) $[C_3 H_4 N_3]^+$	**109.0508**	**$C_4 H_5 N_4$**	**C N**	**H-Het-CN**	**HCN (nuc. addition) †**	**-0.7**	**2E+04**	**N/A**	**N/A**	**N/A**	**1**

Table S3 Continued:

Triazines	Heterocycle m/z (error ppm) Chemical Formula	Measured Mass	Elemental Composition	Side Chain Composition	Assigned Structure	Candidate Reactant	Mass Error (ppm)	Intensity (A.U.) Red. Spk.	Red. Spk. Rep.	Net. Spk .	Net. Spk. Rep.	# Unique Adducts
**32**	**Urazole**	**184.0363**	**$C_6 H_6 O_4 N_3$**	**$C_4 H_5 O_2$**	**Het-C(CH_3)=CH-COOH**	**Methylcyanoacetylene + 2 H_2O**	**-0.2**	**3E+04**	**N/A**	**N/A**	**N/A**	**11**
	m/z = 100.0155 (2.7 ppm)	156.0415	$C_5 H_6 O_3 N_3$	$C_3 H_5 O$	Het-CH_2-CH_2-CHO	Acrolein	0.2	1E+04	N/A	N/A	N/A	
	$[C_2 H_2 O_2 N_3]^-$	153.0418	$C_5 H_5 O_2 N_4$	$C_3 H_4 N$	Het-CH_2-CH_2-CN	Acrylonitrile	0.1	1E+04	N/A	N/A	N/A	
		171.0522	$C_5 H_7 O_3 N_4$	$C_3 H_6 O N$	Het-CH_2-CH_2-$CONH_2$	Acrylonitrile + H_2O	-1.0	1E+04	N/A	N/A	N/A	
		139.0262	$C_4 H_3 O_2 N_4$	$C_2 H_2 N$	Het-CH_2-CN	Glycolonitrile	0.6	1E+04	N/A	N/A	N/A	
		168.0414	$C_6 H_6 O_3 N_3$	$C_4 H_5 O$	Het-C(CH_3)=CH-CHO	Methylpropiolaldehyde	-0.4	1E+04	N/A	N/A	N/A	
		167.0574	$C_6 H_7 O_2 N_4$	$C_4 H_6 N$	Het-CH(CH_3)-CH_2-CN / Het- CH_2-CH(CH_3)-CN	Crotonitrile / Methacrylonitrile	-0.6	8E+03	N/A	N/A	N/A	
		151.0262	$C_5 H_3 O_2 N_4$	$C_3 H_2 N$	Het-CH=CH-CN	Cyanoacetylene	0.4	7E+03	N/A	N/A	N/A	
		157.0366	$C_4 H_5 O_3 N_4$	$C_2 H_4 O N$	Het-CH_2-$CONH_2$	Glycolonitrile + H_2O †	-0.7	6E+03	N/A	N/A	N/A	
		183.0523	$C_6 H_7 O_3 N_4$	$C_4 H_6 O N$	Het-C(CH_3)=CH-$CONH_2$	Methylcyanoacetylene + H_2O	-0.2	6E+03	N/A	N/A	N/A	
		169.0365	$C_5 H_5 O_3 N_4$	$C_3 H_4 O N$	Het-CH=CH-$CONH_2$	Cyanoacetylene + H_2O	-1.0	4E+03	N/A	N/A	N/A	
		172.0362	$C_5 H_6 O_4 N_3$	$C_3 H_5 O_2$	Het-CH_2-CH_2-COOH	Acrylonitrile + 2 H_2O	-0.9	3E+03	N/A	N/A	N/A	
		186.0517	$C_6 H_8 O_4 N_3$	$C_4 H_7 O_2$	Het-CH(CH_3)-CH_2-COOH / Het-CH_2-CH(CH_3)-COOH	Crotonitrile + 2 H_2O / Methacrylonitrile + 2 H_2O †	-1.8	3E+03	N/A	N/A	N/A	
		185.0677	$C_6 H_9 O_3 N_4$	$C_4 H_8 O N$	Het-CH(CH_3)-CH_2-$CONH_2$ / Het-CH_2-CH(CH_3)-$CONH_2$	Crotonitrile + H_2O / Methacrylonitrile + H_2O	-1.7	3E+03	N/A	N/A	N/A	
		172.0474	$C_4 H_6 O_3 N_5$	$C_2 H_5 O N_2$	Het-CH_2-NH-CO-NH_2	Formaldehyde + Urea †	-1.1	3E+03	N/A	N/A	N/A	
		166.0371	$C_5 H_4 O_2 N_5$	$C_3 H_3 N_2$	Het-C(CH_2-CN)=NH	Cyanoacetonitrile (electrophile) †	0.2	2E+03	N/A	N/A	N/A	
		181.0728	$C_7 H_9 O_2 N_4$	$C_5 H_8 N$	Het-CH(CH_2CH_3)-CH_2-CN	Ethyl acrylonitrile	-1.4	2E+03	N/A	N/A	N/A	
		200.0674	$C_7 H_{10} O_4 N_3$	$C_5 H_9 O_2$	Het-CH(CH_2CH_3)-CH_2-COOH	Ethyl acrylonitrile + 2 H_2O	-1.4	2E+03	N/A	N/A	N/A	
		144.0414	$C_4 H_6 O_3 N_3$	$C_2 H_5 O$	Het-CH(OH)-CH_3	Acetaldehyde	-0.6	1E+03	N/A	N/A	N/A	
**33**	**6-azauracil**	186.0509	$C_6 H_8 O_4 N_3$	$C_3 H_5 O_2$	Het-CH_2-CH_2-COOH	Acrylonitrile + 2 H_2O	-0.1	2E+04	N/A	N/A	N/A	**5**
	m/z = 114.0297 (-1.8 ppm) $[C_3 H_4 O_2 N_3]^+$	168.0403	$C_6 H_6 O_3 N_3$	$C_3 H_3 O$	Het-CH=CH=CHO	Propiolaldehyde	-0.5	3E+04	N/A	N/A	N/A	
	m/z = 112.0155 (2.5 ppm)	**184.0363**	**$C_6 H_6 O_4 N_3$**	**$C_3 H_5 O_2$**	**Het-CH_2-CH_2-COOH**	**Acrylonitrile + 2 H_2O**	**-0.5**	**1E+05**	**N/A**	**N/A**	**N/A**	
	$[C_3 H_2 O_2 N_3]^-$	137.0107	$C_4 H O_2 N_4$	C N	Het-CN	HCN (nuc. substitution)	1.3	8E+04	N/A	N/A	N/A	
		183.0522	$C_6 H_7 O_3 N_4$	$C_3 H_6 O N$	Het-CH_2-CH_2-$CONH_2$	Acrylonitrile + H_2O	-0.8	2E+04	N/A	N/A	N/A	
		169.0367	$C_5 H_5 O_3 N_4$	$C_2 H_4 O N$	Het-CH_2-$CONH_2$	Glycolonitrile + H_2O †	-0.3	5E+03	N/A	N/A	N/A	
		163.0261	$C_6 H_3 O_2 N_4$	$C_3 H_2 N$	Het-CH=CH-CN	Cyanoacetylene	-0.2	4E+03	N/A	N/A	N/A	
**34**	**Cyanuric Acid** m/z = 128.0105 (2.8 ppm) $[C_3 H_2 O_3 N_3]^-$	No Adducts Detected							N/A	N/A	N/A	0

Table S3 Continued:

Purines	Heterocycle m/z (error ppm) Chemical Formula	Measured Mass	Elemental Composition	Side Chain Composition	Assigned Structure	Candidate Reactant	Mass Error (ppm)	Intensity (A.U.) Red. Spk.	Red. Spk. Rep.	Net. Spk .	Net. Spk. Rep.	# Unique Adducts
**35**	**2-aminopurine**	**150.0773**	**$C_6H_8N_5$**	**CH_3**	**Het-CH_3**	**Formaldehyde + Formic Acid***	**-1.4**	**5E+05**	**N/A**	**-**	**-**	**16**
	m/z = 136.0616 (-1.6 ppm)	166.0721	$C_6H_8ON_5$	CH_3O	Het-CH_2-OH	Formaldehyde	-1.5	3E+05	N/A	-	-	
	$[C_5H_6N_5]^+$	208.0827	$C_8H_{10}O_2N_5$	$C_3H_5O_2$	Het-CH_2-CH_2-COOH	Acrylonitrile + 2 H_2O	-0.7	2E+05	N/A	-	-	
		180.0878	$C_7H_{10}ON_5$	C_2H_5O	Het-CH(OH)-CH_3	Acetaldehyde	-1.0	1E+05	N/A	6E+05	1E+05	
		177.0881	$C_7H_9N_6$	C_2H_4N	Het-C(CH_3)=NH	Acetic Acid + NH_3	-1.1	7E+04	N/A	-	-	
		207.0987	$C_8H_{11}ON_6$	C_3H_6ON	Het-CH_2-CH_2-$CONH_2$	Acrylonitrile + H_2O	-0.9	6E+04	N/A	4E+04	2E+03	
		193.0831	$C_7H_9ON_6$	C_2H_4ON	Het-CH_2-$CONH_2$	Glycolonitrile + H_2O †	-0.7	6E+04	N/A	-	-	
		165.0881	$C_6H_9N_6$	CH_4N	Het-CH_2-NH_2	†Formaldehyde + NH_3 † or degradation of *m/z* 209.0771	0.9	6E+04	N/A	1E+03	1E+03	
		222.0984	$C_9H_{12}O_2$ N5	$C_4H_7O_2$	Het-CH(CH_3)-CH_2-COOH / Het-CH_2-CH(CH_3)-COOH	Crotonitrile + 2 H_2O / Methacrylonitrile + 2 H_2O †	-3.4	4E+04	N/A	1E+04	2E+04	
		236.114	$C_{10}H_{14}O_2N_5$	$C_5H_9O_2$	Het-CH(CH_2CH_3)-CH_2-COOH	Ethyl acrylonitrile + 2 H_2O	-1.0	3E+04	N/A	-	-	
		194.1034	$C_8H_{12}ON_5$	C_3H_7O	Het-C$(CH_3)_2$-OH	Acetone	-1.0	2E+04	N/A	-	-	
		189.0899	$C_8H_9N_6$	C_3H_4N	Het-CH_2-CH_2-CN	Acrylonitrile	-0.7	2E+04	N/A	8E+04	-	
		187.0726	$C_8H_7N_6$	C_3H_2N	Het-CH=CH-CN	Cyanoacetylene	-0.3	2E+04	N/A	3E+03	3E+03	
		210.0984	$C_8H_{12}O_2N_5$	$C_3H_7O_2$	Het-CH_2-(CHOH)-CH_2OH	Hydroxy acetone	-3.1	1E+04	N/A	-	-	
		205.0830	$C_8H_9ON_6$	C_3H_4ON	Het-CH=CH-$CONH_2$	Cyanoacetylene + H_2O	-1.1	1E+04	N/A	2E+05	2E+04	
		181.0830	$C_6H_9ON_6$	CH_4ON	Het-CH(OH)-NH_2	Formamide (electrophile) †	-1.5	1E+04	N/A	4E+04	-	
		219.0986	$C_9H_{11}ON_6$	C_4H_6ON	Het-C(CH_3)=CH-$CONH_2$	Methylcyanoacetylene + H_2O	-1.5	1E+04	N/A	-	-	
		226.0932	$C_8H_{12}O_3N_5$	$C_3H_7O_3$	Het-CHOH-CHOH-CH_2OH	3-C sugar (electrophile)	-1.4	5E+03	N/A	-	-	
		209.0771	$C_7H_9O_2N_6$	$C_2H_4O_2N$	Het-CH_2-NH-CO-OH	Formaldehyde + Urea + H_2O †	-5.1	3E+03	N/A	-	-	
		268.1050	$C_{10}H_{14}O_4N_5$	$C_5H_9O_4$	Het-5-C sugar	5-C sugar condensation	3.5	1E+03	N/A	1E+03	-	
**36**	**Adenine**	**193.0832**	**$C_7H_9ON_6$**	**C_2H_4ON**	**Het-CH_2-$CONH_2$**	**Glycolonitrile + H_2O †**	**-0.3**	**7E+04**	**7E+03**	**-**	**-**	**4**
	m/z = 136.0614 (-2.7 ppm)	164.0571	$C_6H_6ON_5$	CHO	Het-CHO	Formic Acid	2.6	1E+04	-	2E+04	2E+04	
	$[C_5H_6N_5]^+$	208.0828	$C_8H_{10}O_2N_5$	$C_3H_5O_2$	Het-CH_2-CH_2-COOH	Acrylonitrile + 2 H_2O	-0.4	1E+04	2E+04	-	-	
		219.0986	$C_9H_{11}ON_6$	C_4H_6ON	Het-C(CH_3)=CH-$CONH_2$	Methylcyanoacetylene + H_2O	-1.2	9E+03	1E+04	-	-	
**37**	**2,6-diaminopurine**	**193.0832**	**$C_7H_9ON_6$**	**C_2H_3O**	**Het-CO-CH_3**	**Acetic Acid**	**-0.1**	**2E+05**	**N/A**	**N/A**	**N/A**	**6**
	m/z = 151.0724 (-2.0 ppm)	207.0988	$C_8H_{11}ON_6$	C_3H_5O	Het-CH_2-CH_2-CHO	Acrolein	-0.5	5E+04	N/A	N/A	N/A	
	$[C_5H_7N_6]^+$	190.0835	$C_7H_8N_7$	C_2H_2N	Het-CH_2-CN	Glycolonitrile	-0.2	4E+04	N/A	N/A	N/A	
		205.0832	$C_8H_9ON_6$	C_3H_3O	Het-CH=CH=CHO	Propiolaldehyde	-0.2	3E+04	N/A	N/A	N/A	
		208.0939	$C_7H_{10}ON_7$	C_2H_4ON	Het-CH_2-$CONH_2$	Glycolonitrile + H_2O †	-1.2	2E+04	N/A	N/A	N/A	
		202.0834	$C_8H_8N_7$	C_3H_2N	Het-CH=CH-CN	Cyanoacetylene	-0.6	1E+04	N/A	N/A	N/A	
		219.0986	$C_9H_{11}ON_6$	C_4H_5O	Het-C(CH_3)=CH-CHO	Methylpropiolaldehyde	-1.4	2E+03	N/A	N/A	N/A	

Table S3 Continued:

Purines	Heterocycle<br>m/z (error ppm)<br>Chemical Formula	Measured Mass	Elemental Composition	Side Chain Composition	Assigned Structure	Candidate Reactant	Mass Error (ppm)	Intensity (A.U.)				# Unique Adducts
								Red. Spk.	Red. Spk. Rep.	Net. Spk .	Net. Spk. Rep.	
**38**	**Isoguanine**<br>m/z = 152.0565 (-1.0 ppm)<br>$[C_5 H_6 O N_5]^+$	**206.0672**	**$C_8 H_8 O_2 N_5$**	**$C_3 H_3 O$**	**Het-CH=CH=CHO**	**Propiolaldehyde**	**0.0**	**2E+04**	**N/A**	**-**	**N/A**	**4**
		**205.0821**	**$C_8 H_9 O N_6$**	**$C_3 H_4 N$**	**Het-CH_2-CH_2-CN**	**Acrylonitrile**	**-5.6**	**2E+04**	**N/A**	**6E+03**	**N/A**	
		194.0672	$C_7 H_8 O_2 N_5$	$C_2 H_3 O$	Het-CO-CH_3	Acetic Acid	-0.4	9E+03	N/A	2E+04	N/A	
		220.0832	$C_9 H_{10} O_2 N_5$	$C_4 H_5 O$	Het-C(CH_3)=CH-CHO	Methylpropiolaldehyde	1.2	2E+03	N/A	-	N/A	
**39**	**Guanine**<br>m/z = 152.0573 (4.2 ppm)<br>$[C_5 H_6 O N_5]^+$	**236.0779**	**$C_9 H_{10} O_3 N_5$**	**$C_4 H_5 O_2$**	**Het-C(CH_3)=CH-COOH**	**Methylcyanoacetylene + 2 H_2O**	**0.3**	**N/A**	**1E+04**	**4E+03**	**-**	**3**
		208.0837	$C_8 H_{10} O_2 N_5$	$C_3 H_5 O$	Het-CH_2-CH_2-CHO	Acrolein	4.0	N/A	2E+03	1E+03	-	
		196.0830	$C_7 H_{10} O_2 N_5$	$C_2 H_5 O$	Het-CH(OH)-CH_3	Acetaldehyde	0.4	N/A	1E+03	-	-	
**40**	**Hypoxanthine**<br>m/z = 137.0456 (-1.6 ppm)<br>$[C_5 H_5 N_4 O]^+$	**194.0672**	**$C_7 H_8 O_2 N_5$**	**$C_2 H_4 O N$**	**Het-CH_2-$CONH_2$**	**Glycolonitrile + H_2O †**	**-0.2**	**9E+04**	**N/A**	**N/A**	**N/A**	**2**
		287.0999	$C_{10} H_{15} O_6 N_4$	$C_5 H_{11} O_5$	Het-$(CHOH)_4$-CH_2OH	5-C sugar (electrophile)	4.6	1E+04	N/A	N/A	N/A	
**41**	**3,7-dimethylxanthine**<br>m/z = 181.0718 (-1.2 ppm)<br>$[C_7 H_9 O_2 N_4]^+$	**238.0936**	**$C_9 H_{12} O_3 N_5$**	**$C_2 H_4 O N$**	**Het-CH_2-$CONH_2$**	**Glycolonitrile + H_2O †**	**0.7**	**2E+04**	**N/A**	**N/A**	**N/A**	**3**
		343.1261	$C_{13} H_{19} O_7 N_4$	$C_6 H_{11} O_5$	Het-6-C sugar	6-C sugar condensation	3.6	1E+03	N/A	N/A	N/A	
		299.1357	$C_{12} H_{19} O_5 N_4$	$C_5 H_{11} O_3$	Het-CH_2-C($CH_2OH)_2$-CH_2OH	Acrolein + 2 CH_2O	2.5	1E+03	N/A	N/A	N/A	
**42**	**Xanthine**<br>m/z = 153.0405 (-1.5 ppm)<br>$[C_5 H_5 O_2 N_4]^+$	**210.0623**	**$C_7 H_8 O_3 N_5$**	**$C_2 H_4 O N$**	**Het-CH_2-$CONH_2$**	**Glycolonitrile + H_2O †**	**-0.6**	**2E+04**	**N/A**	**N/A**	**N/A**	**3**
		207.0514	$C_8 H_7 O_3 N_4$	$C_3 H_3 O$	Het-CH=CH=CHO	Propiolaldehyde	0.7	4E+03	N/A	N/A	N/A	
		224.0778	$C_8 H_{10} O_3 N_5$	$C_3 H_6 O N$	Het-CH_2-CH_2-$CONH_2$	Acrylonitrile + H_2O	-0.3	3E+03	N/A	N/A	N/A	
**43**	**1,3-dimethylxanthine**<br>m/z = 181.0718 (-1.1 ppm)<br>$[C_7 H_9 O_2 N_4]^+$	**238.0935**	**$C_9 H_{12} O_3 N_5$**	**$C_2 H_4 O N$**	**Het-CH_2-$CONH_2$**	**Glycolonitrile + H_2O †**	**0.2**	**1E+05**	**N/A**	**N/A**	**N/A**	**5**
		253.0931	$C_{10} H_{13} O_4 N_4$	$C_3 H_5 O_2$	Het-CH_2-CH_2-COOH	Acrylonitrile + 2 H_2O	-0.2	8E+04	N/A	N/A	N/A	
		252.1091	$C_{10} H_{14} O_3 N_5$	$C_3 H_6 O N$	Het-CH_2-CH_2-$CONH_2$	Acrylonitrile + H_2O	0.3	3E+04	N/A	N/A	N/A	
		210.0986	$C_8 H_{12} O_2 N_5$	$C H_4 N$	Het-CH_2-NH_3	Formaldehyde + NH_3 †	0.0	3E+04	N/A	N/A	N/A	
		253.1044	$C_9 H_{13} O_3 N_6$	$C_2 H_5 O N_2$	Het-CH_2-NH-CO-NH_2	Formaldehyde + Urea †	0.1	5E+03	N/A	N/A	N/A	
		247.0938	$C_{10} H_{11} O_2 N_6$	$C_3 H_3 N_2$	Het-C(CH_2-CN)=NH	Cyanoacetonitrile (electrophile) †	0.1	2E+03	N/A	N/A	N/A	
**Pteridines**												
**44**	**6,7-dimethylpterin**<br>m/z = 192.0878 (-0.8 ppm)<br>$[C_8 H_{10} O N_5]^+$	**260.1156**	**$C_{12} H_{14} O_2 N_5$**	**$C_4 H_5 O$**	**Het-C(CH_3)=CH-CHO**	**Methylpropiolaldehyde**	**5.2**	**1E+03**	**N/A**	**N/A**	**N/A**	**1**
**45**	**6-hydroxymethylpterin**<br>m/z = 194.0672 (-0.2 ppm)<br>$[C_7 H_8 O_2 N_5]^+$	No Adducts Detected							N/A	N/A	N/A	**0**

Table S3 Continued:

								Intensity (A.U.)				
**Pteridines**	**Heterocycle m/z (error ppm) Chemical Formula**	**Measured Mass**	**Elemental Composition**	**Side Chain Composition**	**Assigned Structure**	**Candidate Reactant**	**Mass Error (ppm)**	**Red. Spk.**	**Red. Spk. Rep.**	**Net. Spk .**	**Net. Spk. Rep.**	**# Unique Adducts**
**46**	**2,4-diamino-6-hydroxymethylpteridine**	**220.0941**	**$C_8 H_{10} O N_7$**	**C N / C H_2 N**	**H-Het-CN / Het-CH=NH**	**HCN (nuc. addition) / Formic Acid + NH_3**	**0.1**	**6E+04**	**N/A**	**N/A**	**N/A**	**6**
	m/z = 193.0831 (-1.0 ppm)	208.0942	$C_7 H_{10} O N_7$	H_2 N	Het-NH_2	Ammonia	0.1	3E+04	N/A	N/A	N/A	
	$[C_7 H_9 O N_6]^+$	235.0938	$C_9 H_{11} O_2 N_6$	$C_2 H_3$ O	Het-CO-CH_3	Acetic Acid	0.0	3E+04	N/A	N/A	N/A	
		218.0784	$C_8 H_8 O N_7$	C N	Het-CN	HCN (nuc. substitution)	-0.2	2E+04	N/A	N/A	N/A	
		249.1093	$C_{10} H_{13} O_2 N_6$	$C_3 H_5$ O	Het-CH_2-CH_2-CHO	Acrolein	-0.3	7E+03	N/A	N/A	N/A	
		236.0889	$C_8 H_{10} O_2 N_8$	C H_2 O N	Het-$CONH_2$	HCN + H_2O (nuc. substitution)	-0.5	6E+03	N/A	N/A	N/A	
		232.0940	$C_9 H_{10} O N_7$	$C_2 H_2$ N	Het-CH_2-CN	Glycolonitrile	0.8	3E+03	N/A	N/A	N/A	
**47**	**Xanthopterin**	**205.0476**	**$C_7 H_5 O_2 N_6$**	**C N / C H_2 N**	**H-Het-CN / Het-CH=NH**	**HCN (nuc. addition) / Formic Acid + NH_3**	**-1.7**	**7E+04**	**N/A**	**N/A**	**N/A**	**16**
	m/z = 178.0372 (1.0 ppm)	219.0631	$C_8 H_7 O_2 N_6$	$C_2 H_4$ N	Het-C(CH_3)=NH	Acetic Acid + NH_3	-2.1	6E+04	N/A	N/A	N/A	
	$[C_6 H_4 O_2 N_5]^-$	231.0629	$C_9 H_7 O_2 N_6$	$C_3 H_4$ N	Het-CH_2-CH_2-CN	Acrylonitrile	-2.8	2E+04	N/A	N/A	N/A	
		232.0471	$C_9 H_6 O_3 N_5$	$C_3 H_3$ O	Het-CH=CH=CHO	Propiolaldehyde	-2.4	2E+04	N/A	N/A	N/A	
		234.0627	$C_9 H_8 O_3 N_5$	$C_3 H_5$ O	Het-CH_2-CH_2-CHO	Acrolein	-2.6	1E+04	N/A	N/A	N/A	
		249.0734	$C_9 H_9 O_3 N_6$	$C_3 H_6$ O N	Het-CH_2-CH_2-$CONH_2$	Acrylonitrile + H_2O	-3.1	1E+04	N/A	N/A	N/A	
		245.0785	$C_{10} H_9 O_2 N_6$	$C_4 H_6$ N	Het-CH(CH_3)-CH_2-CN / Het-CH_2-CH(CH_3)-CN	Crotonitrile / Methacrylonitrile	-3.0	1E+04	N/A	N/A	N/A	
		263.0890	$C_{10} H_{11} O_3 N_6$	$C_4 H_8$ O N	Het-CH(CH_3)-CH_2-$CONH_2$ / Het-CH_2-CH(CH_3)- $CONH_2$	Crotonitrile + H_2O / Methacrylonitrile + H_2O	-3.1	1E+04	N/A	N/A	N/A	
		235.0579	$C_8 H_7 O_3 N_6$	$C_2 H_4$ O N	Het-CH_2-$CONH_2$	Glycolonitrile + H_2O †	-2.6	1E+04	N/A	N/A	N/A	
		246.0626	$C_{10} H_8 O_3 N_5$	$C_4 H_5$ O	Het-C(CH_3)=CH-CHO	Methylpropiolaldehyde	-2.5	9E+03	N/A	N/A	N/A	
		223.0579	$C_7 H_7 O_3 N_6$	C H_2 O N	H-Het-$CONH_2$	HCN + H_2O (nuc. addition) †	-2.9	9E+03	N/A	N/A	N/A	
		207.0627	$C_7 H_7 O_2 N_6$	C H_4 N	Het-NH-CH_3 / Het-CH_2-NH_2	Methylamine / Formaldehyde + NH_3 †	-4.4	7E+03	N/A	N/A	N/A	
		248.0784	$C_{10} H_{10} O_3 N_5$	$C_4 H_7$ O	Het-CH(CH_3)-CH_2-CHO / Het-CH_2-CH(CH_3)-CHO	Crotonaldehyde / Methacrolein	-2.1	5E+03	N/A	N/A	N/A	
		248.0419	$C_9 H_6 O_4 N_5$	$C_3 H_3 O_2$	Het-CH=CH-COOH	Cyanoacetylene + 2 H_2O	-2.4	5E+03	N/A	N/A	N/A	
		238.0578	$C_8 H_8 O_4 N_5$	$C_2 H_5 O_2$	Het-CH(OH)-CH_2-OH	Glycolaldehyde	-1.6	5E+03	N/A	N/A	N/A	
		243.0629	$C_{10} H_7 O_2 N_6$	$C_4 H_4$ N	Het-C(CH_3)=CH-CN	Methylcyanoacetylene	1.6	5E+03	N/A	N/A	N/A	
		229.0470	$C_9 H_5 O_2 N_6$	$C_3 H_2$ N	Het-CH=CH-CN	Cyanoacetylene	-3.9	4E+03	N/A	N/A	N/A	
		247.0579	$C_9 H_7 O_3 N_6$	$C_3 H_4$ O N	Het-CH=CH-$CONH_2$	Cyanoacetylene + H_2O	-2.4	3E+03	N/A	N/A	N/A	
		262.0579	$C_{10} H_8 O_4 N_5$	$C_4 H_5 O_2$	Het-C(CH_3)=CH-COOH	Methylcyanoacetylene + 2 H_2O	-1.0	3E+03	N/A	N/A	N/A	
		252.0733	$C_9 H_{10} O_4 N_5$	$C_3 H_7 O_2$	Het-CH_2-(CHOH)-CH_2OH	Hydroxy acetone	-2.1	2E+03	N/A	N/A	N/A	
		250.0576	$C_9 H_8 O_4 N_5$	$C_3 H_5 O_2$	Het-CH_2-CH_2-COOH	Acrylonitrile + 2 H_2O	-2.3	2E+03	N/A	N/A	N/A	
		265.0678	$C_9 H_9 O_4 N_6$	$C_3 H_6 O_2$ N	Het-NH-CH(CH_3)-COOH	Alanine (nuc. substitution)	-4.7	2E+03	N/A	N/A	N/A	
		259.0938	$C_{11} H_{11} O_2 N_6$	$C_5 H_8$ N	Het-CH(CH_2CH_3)-CH_2-CN	Ethyl acrylonitrile	-4.3	2E+03	N/A	N/A	N/A	
		221.0427	$C_7 H_5 O_3 N_6$	C H_2 O N	Het-$CONH_2$	HCN + H_2O (nuc. substitution)	-4.1	1E+03	N/A	N/A	N/A	
		264.0731	$C_{10} H_{10} O_4 N_5$	$C_4 H_7 O_2$	Het-CH(CH_3)-CH_2-COOH / Het-CH_2-CH(CH_3)-COOH	Crotonitrile + 2 H_2O / Methacrylonitrile + 2 H_2O †	-2.8	1E+03	N/A	N/A	N/A	

Table S3 Continued:

Pteridines	Heterocycle<br>m/z (error ppm)<br>Chemical Formula	Measured Mass	Elemental Composition	Side Chain Composition	Assigned Structure	Candidate Reactant	Mass Error (ppm)	Intensity (A. U.)				# Unique Adducts
								Red. Spk.	Red. Spk. Rep.	Net. Spk .	Net. Spk. Rep.	
**48**	**Isoxanthopterin**<br>m/z = 178.0370 (-0.3 ppm)<br>$[C_6 H_4 O_2 N_5]^-$	**203.0318**	**$C_7 H_3 O_2 N_6$**	**C N**	**Het-CN**	**HCN (nuc. substitution)**	**-2.6**	**3E+03**	**N/A**	**N/A**	**N/A**	**4**
		246.0644	$C_{10} H_8 O_3 N_5$	$C_4 H_5 O$	Het-C(CH_3)=CH-CHO	Methylpropiolaldehyde	4.6	2E+03	N/A	N/A	N/A	
		234.0639	$C_9 H_8 O_3 N_5$	$C_3 H_5 O$	Het-CH_2-CH_2-CHO	Acrolein	2.7	2E+03	N/A	N/A	N/A	
		231.0622	$C_9 H_7 O_2 N_6$	$C_3 H_4 N$	Het-CH_2-CH_2-CN	Acrylonitrile	-5.1	1E+03	N/A	N/A	N/A	
**49**	**Pterine**<br>m/z = 164.0565 (-1.4 ppm)<br>$[C_6 H_6 O N_5]^+$	**218.0672**	**$C_9 H_8 O_2 N_5$**	**$C_3 H_3 O$**	**Het-CH=CH=CHO**	**Propiolaldehyde**	**-0.2**	**4E+04**	**N/A**	**N/A**	**N/A**	**3**
		**207.0625**	**$C_7 H_7 O_2 N_6$**	**$C H_2 O N$**	**Het-$CONH_2$**	**HCN + H_2O (nuc. substitution)**	**-0.1**	**4E+04**	**N/A**	**N/A**	**N/A**	
		189.0518	$C_7 H_5 O N_6$	C N	Het-CN	HCN (nuc. substitution)	-0.7	9E+03	N/A	N/A	N/A	
		232.0829	$C_{10} H_{10} O_2 N_5$	$C_4 H_5 O$	Het-C(CH_3)=CH-CHO	Methylpropiolaldehyde	-0.1	4E+03	N/A	N/A	N/A	
**50**	**6-Biopterin**<br>m/z = 238.0935 (-0.0 ppm)<br>$[C_9 H_{12} O_3 N_5]^+$	**277.1046**	**$C_{11} H_{13} O_3 N_6$**	**$C_2 H_2 N$**	**Het-CH_2-CN**	**Glycolonitrile**	**0.7**	**4E+03**	**N/A**	**N/A**	**N/A**	**1**
**51**	**Pterine-6-carboxylic acid**<br>m/z = 208 (pos. mode)<br>(ND in pos. or neg. mode)<br>$[C_7 H_6 N_5 O_3]^+$	No Adducts Detected							N/A	N/A	N/A	**0**
**52**	**Lumazine**<br>m/z = 165.0404 (-1.6 ppm)<br>$[C_6 H_5 O_2 N_4]^+$	**208.0465**	**$C_7 H_6 O_3 N_5$**	**$C H_2 O N$**	**Het-$CONH_2$**	**HCN + H_2O (nuc. substitution)**	**0.0**	**2E+05**	**N/A**	**N/A**	**N/A**	**3**
		222.0623	$C_8 H_8 O_3 N_5$	$C_2 H_4 O N$	Het-CH_2-$CONH_2$	Glycolonitrile + H_2O †	0.8	1E+04	N/A	N/A	N/A	
		221.0670	$C_9 H_9 O_3 N_5$	$C_3 H_5 O$	Het-CH_2-CH_2-CHO	Acrolein	0.3	2E+03	N/A	N/A	N/A	
**53**	**Alloxazine**<br>m/z = 215.0562 (-0.7 ppm)<br>$[C_{10} H_7 O_2 N_4]^+$	No Adducts Detected							N/A	N/A	N/A	**0**

Table S4. Target adducts identified from N-heterocycles incubated with a spark discharge mixture generated under a neutral atmosphere. Reaction mixtures were either frozen immediately (-80 ℃) or incubated at 80 ℃ for 3 or 7 days. Target adducts with the highest intensities in their respective spectrum are bolded. The intensity of the same adduct identified in replicate mixtures and those generated under a reducing atmosphere are provided. **Acronyms**: A.U. (arbitrary unites); ND (no adducts detected); N/A (not analyzed); Net. Spk. (spark mixture generated under a neutral atmosphere of N_2 and CO_2 gases); Red. Spk. (spark mixture generated under a reducing atmosphere of N_2, CO_2, H_2, and CH_4 gases); Rep. (spark replicate); nuc. (nucleophilic). Dashes indicate adduct was not detected above thresholds. * indicates an adduct that has the highest intensity in the reaction spectrum but was not targeted for analysis. The total number of target adducts generated from different reactants is listed. Thus, adducts generated from the same reactant, but varying degrees of hydrolysis counted only once toward the total listed (e.g., adducts from glycolonitrile, glycolonitrile + H_2O, and glycolonitrile + 2 H_2O would count as one unique adduct); similarly, adducts generated from nucleophilic addition and substitution from the same reactant only counted once (e.g., H-Het-CN and Het-CN). Lastly, products in equilibrium with one another were also counted as a single unique adduct (e.g., adducts from formic acid (Het-CHO) and formic acid + NH_3 (Het-CHNH)—being able to readily convert to one another in the Miller-Urey mixture—count as a single unique adduct).

								Intensity (A.U.)				
**Pyridines**	**Heterocycle m/z (error ppm) Chemical Formula**	**Measured Mass**	**Elemental Composition**	**Side Chain Composition**	**Assigned Structure**	**Candidate Reactant**	**Mass Error (ppm)**	**Net. Spk.**	**Net. Spk. Rep.**	**Red. Spk.**	**Red. Spk. Rep.**	**# Unique Adducts**
1	**2-aminopyridine**	**176.119**	**$C_9 H_{14} O_3 N_3$**	**$C_5 H_8 N$**	**Het-$CH(CH_2CH_3)$-CH_2-CN**	**Ethyl-acrylonitrile**	**4.7**	**4E+05**	**-**	**3E+04**	**-**	**19**
	m/z = 95.0604 (0.1 ppm)	163.0866	$C_9 H_{11} O N_2$	$C_4 H_5 O$	Het-$C(CH_3)$=CH-CHO	Methylpropiolaldehyde	0.2	2E+05	1E+03	-	-	
	$[C_5 H_7 N_2]^+$	134.0713	$C_7 H_8 N_3$	$C_2 H_2 N$	Het-CH_2-CN	Glycolonitrile	0.4	-	4E+04	6E+05	1E+04	
		162.1034	$C_9 H_{12} N_3$	$C_4 H_6 N$	Het-$CH(CH_3)$-CH_2-CN / Het-CH_2-$CH(CH_3)$- CN	Crotonitrile / Methacrylonitrile	4.9	2E+04	-	5E+05	1E+03	
		164.0811	$C_8 H_{10} O N_3$	$C_3 H_4 O N$	Het-CH=CH-$CONH_2$	Cyanoacetylene + H_2O	-4.5	2E+04	2E+03	-	-	
		137.0711	$C_7 H_9 O N_2$	$C_2 H_3 O$	Het-CO-CH_3	Acetic acid	1.5	-	1E+04	-	-	
		123.0561	$C_6 H_7 O N_2$	C O	Het-CHO	Formic Acid	6.4	-	1E+04	-	-	
		149.0821	$C_7 H_9 N_4$	$C_2 H_5 N_2$	Het-$C(CH_2$-$NH_2)$=NH	Glycine nitrile (electrophile)	-0.3	-	9E+03	-	-	
		125.0712	$C_6 H_9 O N_2$	C H_3 O	Het-CH_2-OH	Formaldehyde	2.3	-	8E+03	-	-	
		179.0815	$C_9 H_{11} O_2 N_2$	$C_4 H_5 O_2$	Het-$C(CH_3)$=CH-COOH	Methylcyanoacetylene + 2 H_2O	0.0	-	8E+03	7E+05	2E+05	
		141.0660	$C_6 H_9 O_2 N_2$	C H_2 O_2	H-Het-COOH	HCN + 2 H_2O (nuc. addition)	1.3	-	7E+03	-	-	
		152.0813	$C_7 H_{10} O N_3$	$C_2 H_4$ O N	Het-CH_2-$CONH_2$	Glycolonitrile + H_2O †	-3.5	-	6E+03	-	-	
		151.0865	$C_8 H_{11} O N_2$	$C_3 H_5 O$	Het-CH_2-CH_2-CHO	Acrolein	-1.9	-	5E+03	-	-	
		155.0814	$C_7 H_{11} O_2 N_2$	$C_2 H_5 O_2$	Het-CH(OH)-CH_2-OH	Glycolaldehyde	-0.5	-	5E+03	-	-	
		146.0715	$C_8 H_8 N_3$	$C_3 H_2 N$	Het-CH=CH-CN	Cyanoacetylene	1.7	5E+03	1E+03	2E+05	2E+03	
		139.0865	$C_7 H_{11} O N_2$	$C_2 H_5 O$	Het-CH(OH)-CH_3	Acetaldehyde	-0.4	-	4E+03	-	-	
		148.0862	$C_8 H_{10} N_3$	$C_3 H_4 N$	Het-CH_2-CH_2-CN	Acrylonitrile	5.2	1E+03	4E+03	1E+06	6E+03	
		167.0813	$C_8 H_{11} O_2 N_2$	$C_3 H_5 O_2$	Het-CH_2-CH_2-COOH	Acrylonitrile + 2 H_2O	-1.3	-	4E+03	-	-	
		152.0942	$C_6 H_{10} N_5$	C H_4 N_3	Het-NH-CNH-NH_2	Guanidine (nuc. substitution)	7.7	4E+03	-	-	-	
		167.0934	$C_7 H_{11} O N_4$	$C_2 H_5 O N_2$	Het-CH_2-NH-CO-NH_2	Formaldehyde + Urea †	4.2	3E+03	-	-	-	
		166.0977	$C_8 H_{12} O N_3$	$C_3 H_6$ O N	Het-CH_2-CH_2-$CONH_2$	Acrylonitrile + H_2O	1.3	-	3E+03	-	-	
		120.0564	$C_6 H_6 N_3$	C N	Het-NH-CN	Nitrosation + Cyanamide †	6.9	-	3E+03	-	-	
		165.1020	$C_9 H_{13} O N_2$	$C_4 H_7 O$	Het-$CH(CH_3)$-CH_2-CHO / Het-CH_2-$CH(CH_3)$-CHO	Crotonaldehyde / Methacrolein	-1.7	-	2E+03	-	-	

Table S4 Continued:

Pyridines	Heterocycle m/z (error ppm) Chemical Formula	Measured Mass	Elemental Composition	Side Chain Composition	Assigned Structure	Candidate Reactant	Mass Error (ppm)	Intensity (A.U.) Net. Spk.	Net. Spk. Rep.	Red. Spk.	Red. Spk. Rep.	# Unique Adducts
1	**2-aminopyridine** m/z = 95.0604 (0.1 ppm) $[C_5 H_7 N_2]^+$	165.0656	$C_8 H_9 O_2 N_2$	$C_3 H_3 O_2$	Het-CH=CH-COOH	Cyanoacetylene +2 H_2O	-1.4	-	2E+03	-	-	
		124.0869	$C_6 H_{10} N_3$	$C H_4 N$	Het-NH-CH_3	Methyl amine	0.0	-	2E+03	-	-	
		122.0717	$C_6 H_8 N_3$	H C N	Het-CH=NH	Formic Acid + NH_3 †	3.3	-	2E+03	-	-	
		136.0874	$C_7 H_{10} N_3$	$C_2 H_4 N$	Het-C(CH_3)=NH	Acetic Acid + NH_3	3.6	-	1E+03	-	-	
		180.1134	$C_9 H_{14} O N_3$	$C_4 H_8 O N$	Het-CH(CH_3)-CH_2-$CONH_2$ / Het-CH_2-CH(CH_3)- $CONH_2$	Crotonitrile + H_2O / Methacrylonitrile + H_2O	1.7	-	1E+03	-	-	
		168.0769	$C_7 H_{10} O_2 N_3$	$C_2 H_4 O_2 N$	Het-CH_2-NH-CO-OH	Formaldehyde + Urea + H_2O †	0.8	-	1E+03	-	-	
		153.0660	$C_7 H_9 O_2 N_2$	$C_2 H_3 O_2$	Het-CH_2-COOH	Glycolonitrile + 2 H_2O †	1.1	-	1E+03	-	-	
3	**3-aminopyridine** m/z = 95.0608 (4.8 ppm) $[C_5 H_7 N_2]^+$	**123.0553**	**$C_6 H_7 O N_2$**	**C O**	**Het-CHO**	**Formic Acid**	**-0.2**	**-**	**4E+04**	**-**	**-**	**8**
		139.0871	$C_7 H_{11} O N_2$	$C_2 H_5 O$	Het-CH(OH)-CH_3	Acetaldehyde	3.5	-	2E+04	-	-	
		164.0820	$C_8 H_{10} O N_3$	$C_3 H_4 O N$	Het-CH=CH-$CONH_2$	Cyanoacetylene + H_2O	0.7	-	2E+04	-	-	
		120.0556	$C_6 H_6 N_3$	C N	Het-NH-CN	Nitrosation + Cyanamide †	0.1	-	1E+04	-	-	
		137.0714	$C_7 H_9 O N_2$	$C_2 H_3 O$	Het-CO-CH_3	Acetic acid	3.5	-	6E+03	-	-	
		178.0982	$C_9 H_{12} O N_3$	$C_4 H_6 O N$	Het-C(CH_3)=CH-$CONH_2$	Methylcyanoacetylene + H_2O	3.8	-	3E+03	-	-	
		152.0806	$C_7 H_{10} O N_3$	$C_2 H_4 O N$	Het-CH_2-$CONH_2$	Glycolonitrile + H_2O †	-8.0	2E+03	1E+03	1E+07	2E+06	
		134.0706	$C_7 H_8 N_3$	$C_2 H_2 N$	Het-CH_2-CN	Glycolonitrile	-4.7	1E+03	-	9E+05	4E+04	
		243.0979	$C_{10} H_{15} O_5 N_2$	$C_5 H_9 O_5$	Het-CO-$(CHOH)_3$-CH_2OH	Formic Acid + 4-Carbon sugar/ 5-carbon γ-lactone	1.4	1E+03	-	-	-	
7	**2-pyridinecarboxylic acid** m/z = 124.0390 (-2.5 ppm) $[C_6 H_6 O_2 N]^+$	**198.0761**	**$C_9 H_{12} O_4 N$**	**$C_3 H_7 O_2$**	**Het-CH_2-(CHOH)-CH_2OH**	**Hydroxy acetone †**	**0.0**	**N/A**	**3E+03**	**-**	**N/A**	**1**
8	**3-pyridinecarboxylic acid** m/z = 124.0391 (-1.9 ppm) $[C_6 H_6 O_2 N]^+$	**168.0659**	**$C_8 H_{10} O_3 N$**	**$C_2 H_5 O$**	**Het-CH(OH)-CH_3**	**Acetaldehyde**	**2.4**	**N/A**	**4E+04**	**-**	**N/A**	**7**
		195.0761	$C_9 H_{11} O_3 N_2$	$C_3 H_6 O N$	Het-CH_2-CH_2-$CONH_2$	Acrylonitrile + H_2O	-1.6	N/A	1E+04	-	N/A	
		196.0714	$C_8 H_{10} O_3 N_3$	$C_2 H_5 O N_2$	Het-NH-CH_2-$CONH_2$ / Het-CH_2-NH-CO-NH_2	Glycine amide (nuc. substitution) / Formaldehyde + Urea	-1.2	N/A	5E+03	-	-	
		192.0660	$C_{10} H_{10} O_3 N$	$C_4 H_5 O$	Het-C(CH_3)=CH-CHO	Methylpropiolaldehyde	2.7	N/A	2E+03	-	N/A	
		189.0658	$C_{10} H_9 O_2 N_2$	$C_4 H_4 N$	Het-C(CH_3)=CH-CN	Methylcyanoacetylene	-0.1	N/A	2E+03	-	N/A	
		181.0732	$C_7 H_9 O_2 N_4$	$C H_4 N_3$	Het-NH-CNH-NH_2	Guanidine (nuc. substitution)	6.4	N/A	1E+03	-	-	
		228.0872	$C_{10} H_{14} O_5 N$	$C_4 H_9 O_3$	Het-CH_2-$(CHOH)_2$-CH_2OH	4-C sugar alcohol condensation	2.7	N/A	1E+03	-	N/A	
9	**4-pyridinecarboxylic acid** m/z = 124.0390 (-2.7 ppm) $[C_6 H_6 O_2 N]^+$	**168.0661**	**$C_8 H_{10} O_3 N$**	**$C_2 H_5 O$**	**Het-CH(OH)-CH_3**	**Acetaldehyde**	**3.5**	**N/A**	**1E+05**	**-**	**N/A**	**5**
		196.0711	$C_8 H_{10} O_3 N_3$	$C_2 H_5 O N_2$	Het-NH-CH_2-$CONH_2$ / Het-CH_2-NH-CO-NH_2	Glycine amide (nuc. substitution) / Formaldehyde + Urea	-2.6	N/A	2E+04	-	-	
		195.0763	$C_9 H_{11} O_3 N_2$	$C_3 H_6 O N$	Het-CH_2-CH_2-$CONH_2$	Acrylonitrile + H_2O	-0.8	N/A	1E+04	-	N/A	
		197.0552	$C_8 H_9 O_4 N_2$	$C_2 H_4 O_2 N$	Het-NH-CH_2-COOH / Het-CH_2-NH-CO-OH	Glycine (nuc. substitution) / Formaldehyde + Urea + H_2O	-2.6	N/A	4E+03	-	N/A	
		210.0753	$C_{10} H_{12} O_4 N$	$C_4 H_7 O_2$	Het-CH(CH_3)-CH_2-COOH / Het-CH_2-CH(CH_3)-COOH	Crotonitrile + 2 H_2O / Methacrylonitrile + 2 H_2O †	-3.7	N/A	3E+03	-	N/A	
		166.0494	$C_8 H_8 O_3 N$	$C_2 H_3 O$	Het-CO-CH_3	Acetic Acid	-2.8	N/A	1E+03	-	N/A	

Table S4 Continued:

Pyrimidines	Heterocycle m/z (error ppm) Chemical Formula	Measured Mass	Elemental Composition	Side Chain Composition	Assigned Structure	Candidate Reactant	Mass Error (ppm)	Intensity (A.U.) Net. Spk.	Net. Spk. Rep.	Red. Spk.	Red. Spk. Rep.	# Unique Adducts
**15**	**2,4-diaminopyrimidine**	136.0625	$C_5 H_6 N_5$	C N	Het-NH-CN	Nitrosation + Cyanamide †	5.1	5E+05	-	N/A	-	**13**
	m/z = 111.0671 (5.3 ppm)	162.0780	$C_7 H_8 N_5$	$C_3 H_2 N$	Het-CH=CH-CN	Cyanoacetylene	3.6	2E+05	1E+04	N/A	-	
	$[C_4 H_7 N_4]^+$	155.0932	$C_6 H_{11} O N_4$	$C_2 H_5 O$	Het-CH(OH)-CH_3	Acetaldehyde	2.7	1E+05	-	N/A	-	
		180.0890	$C_7 H_{10} O N_5$	$C_3 H_4 O N$	Het-CH=CH-$CONH_2$	Cyanoacetylene + H_2O	5.7	9E+04	4E+04	N/A	-	
		139.0618	$C_5 H_7 O N_4$	C H O	Het-CHO	Formic Acid	2.5	-	7E+04	N/A	-	
		192.1242	$C_9 H_{14} N_5$	$C_5 H_8 N$	Het-CH(CH_2CH_3)-CH_2-CN	Ethyl-acrylonitrile	-1.0	2E+04	-	-	-	
		164.0941	$C_7 H_{10} N_5$	$C_3 H_4 N$	Het-CH_2-CH_2-CN	Acrylonitrile	6.3	-	1E+04	N/A	-	
		231.1094	$C_8 H_{15} O_4 N_4$	$C_4 H_9 O_4$	Het-$(CHOH)_3$-CH_2OH	4-C sugar (electrophile)	2.9	-	1E+04	N/A	-	
		168.0891	$C_6 H_{10} O N_5$	$C_2 H_4 O N$	Het-CH_2-$CONH_2$	Glycolonitrile + H_2O †	6.7	1E+04	-	N/A	8E+03	
		185.1028	$C_7 H_{13} O_2 N_4$	$C_3 H_7 O_2$	Het-CH_2-(CHOH)-CH_2OH	Hydroxy acetone †	-2.9	7E+03	-	N/A	-	
		196.1181	$C_8 H_{14} O N_5$	$C_4 H_8 O N$	Het-CH(CH_3)-CH_2-$CONH_2$/ Het-CH_2-CH(CH_3)-$CONH_2$	Crotonitrile + H_2O / Methacrylonitrile + H_2O	-6.1	-	4E+03	N/A	-	
		229.0934	$C_8 H_{13} O_4 N_4$	$C_4 H_7 O_4$	Het-CO-$(CHOH)_2$-CH_2OH	Formic Acid + 3-Carbon sugar/ 4-carbon γ-lactone	6.5	2E+03	1E+03	N/A	-	
		182.1037	$C_7 H_{12} O N_5$	$C_3 H_6 O N$	Het-CH_2-CH_2-$CONH_2$	Acrylonitrile + H_2O	0.6	-	1E+03	N/A	-	
		243.1093	$C_9 H_{15} O_4 N_4$	$C_5 H_9 O_4$	Het-5-C sugar	5-C sugar condensation	2.1	-	1E+03	N/A	7E+03	
		259.1037	$C_9 H_{15} O_5 N_4$	$C_5 H_9 O_5$	Het-CO$(CHOH)_4$-CH_2OH	Formic Acid + 4-Carbon sugar/ 5-carbon γ-lactone	0.0	-	1E+03	N/A	-	
**16**	**2,4,6-triaminopyrimidine**	**170.1038**	**$C_6 H_{12} O N_5$**	**$C_2 H_5 O$**	**Het-CH(OH)-CH_3**	**Acetaldehyde**	**0.7**	**1E+05**	**9E+04**	**N/A**	**-**	**10**
	m/z = 126.0784 (8.1 ppm)	191.1055	$C_8 H_{11} N_6$	$C_4 H_4 N$	Het-C(CH_3)=CH-CN	Methylcyanoacetylene	8.0	1E+05	-	N/A	1E+06	
	$[C_4 H_8 N_5]^+$	165.0883	$C_6 H_9 N_6$	$C_2 H_2 N$	Het-CH_2-CN	Glycolonitrile	0.1	9E+04	4E+04	N/A	4E+06	
		154.0719	$C_5 H_8 O N_5$	C H O	Het-CHO	Formic Acid	4.7	2E+04	5E+04	N/A	3E+04	
		168.0882	$C_6 H_{10} O N_5$	$C_2 H_3 O$	Het-CO-CH_3	Acetic Acid	1.1	-	1E+04	N/A	-	
		212.1162	$C_8 H_{14} O_2 N_5$	$C_4 H_7 O_2$	Het-CH(CH_3)-CH_2-COOH / Het-CH_2-CH(CH_3)-COOH	Crotonitrile + 2 H_2O / Methacrylonitrile + 2 H_2O †	9.2	-	1E+04	N/A	3E+03	
		195.0984	$C_7 H_{11} O N_6$	$C_3 H_4 O N$	Het-CH=CH-$CONH_2$	Cyanoacetylene + H_2O	-2.6	-	2E+04	N/A	-	
		151.0732	$C_5 H_7 N_6$	C N	Het-NH-CN	Nitrosation + Cyanamide †	3.5	-	4E+04	N/A	-	
		186.0984	$C_6 H_{12} O_2 N_5$	$C_2 H_5 O_2$	Het-CH(OH)-CH_2-OH	Glycolaldehyde	-0.8	5E+03	-	N/A	-	
		174.0984	$C_5 H_{12} O_2 N_5$	$C_3 H_7 O_2$	Het-CH_2-(CHOH)-CH_2OH	Hydroxy acetone †	-0.8	2E+03	-	N/A	-	

Table S4 Continued:

								Intensity (A.U.)				
**Pyrimidines**	**Heterocycle m/z (error ppm) Chemical Formula**	**Measured Mass**	**Elemental Composition**	**Side Chain Composition**	**Assigned Structure**	**Candidate Reactant**	**Mass Error (ppm)**	**Net. Spk.**	**Net. Spk. Rep.**	**Red. Spk.**	**Red. Spk. Rep.**	**# Unique Adducts**
**17**	**Cytosine** m/z = 112.0512 (7.1 ppm) $[C_4H_6ON_3]^+$	**163.0622**	**$C_7H_7ON_4$**	**C_3H_2N**	**Het-CH=CH-CN**	**Cyanoacetylene**	**4.5**	**4E+05**	**N/A**	**-**	**-**	**4**
		179.0931	$C_8H_{11}ON_4$	C_4H_6N	Het-CH(CH_3)-CH_2-CN / Het-CH_2-CH(CH_3)-CN	Crotonitrile / Methacrylonitrile	1.9	5E+03	N/A	-	-	
		154.0606	$C_6H_8O_2N_3$	C_2H_3O	Het-CO-CH_3	Acetic Acid	-3.1	1E+03	N/A	-	-	
		184.0725	$C_7H_{10}O_3N_3$	$C_3H_5O_2$	Het-CH_2-CH_2-COOH	Acrylonitrile + 2 H_2O	4.5	1E+03	N/A	-	-	
**18**	**Isocytosine** m/z = 112.0513 (8.1 ppm) $[C_4H_6ON_3]^+$	**156.0768**	**$C_6H_{10}O_2N_3$**	**C_2H_5O**	**Het-CH(OH)-CH_3**	**Acetaldehyde**	**0.6**	**8E+05**	**N/A**	**-**	**N/A**	**9**
		198.0863	$C_8H_{12}O_3N_3$	$C_4H_7O_2$	Het-CH(CH_3)-CH_2-COOH / Het-CH_2-CH(CH_3)-COOH	Crotonitrile + 2 H_2O / Methacrylonitrile + 2 H_2O †	-5.1	9E+04	N/A	-	N/A	
		181.0716	$C_7H_9O_2N_4$	C_3H_4ON	Het-CH=CH-$CONH_2$	Cyanoacetylene + H_2O	-1.9	9E+04	N/A	-	N/A	
		153.0767	$C_6H_9ON_4$	C_2H_4N	Het-C(CH_3)=NH	Acetic Acid + NH_3	-2.8	7E+04	N/A	-	N/A	
		183.0868	$C_7H_{11}O_2N_4$	C_3H_6ON	Het-CH_2-CH_2-$CONH_2$	Acrylonitrile + H_2O	-4.7	6E+04	N/A	-	N/A	
		164.0562	$C_6H_6ON_5$	C_2HN_2	Het-C(CN)=NH	Cyanogen	-2.8	5E+04	N/A	-	N/A	
		179.0920	$C_8H_{11}ON_4$	C_4H_6N	Het-CH(CH_3)-CH_2-CN / Het-CH_2-CH(CH_3)-CN	Crotonitrile / Methacrylonitrile	-4.2	3E+04	N/A	-	N/A	
		197.1041	$C_8H_{13}O_2N_4$	C_4H_8ON	Het-CH(CH_3)-CH_2-$CONH_2$/ Het-CH_2-CH(CH_3)-$CONH_2$	Crotonitrile + H_2O / Methacrylonitrile + H_2O	4.2	3E+04	N/A	-	N/A	
		165.0764	$C_7H_9ON_4$	C_3H_4N	Het-CH_2-CH_2-CN	Acrylonitrile	-4.2	2E+04	N/A	-	N/A	
		163.0620	$C_7H_7ON_4$	C_3H_2N	Het-CH=CH-CN	Cyanoacetylene	3.5	2E+04	N/A	-	N/A	
		169.0731	$C_6H_9O_2N_4$	C_2H_4ON	Het-CH_2-$CONH_2$	Glycolonitrile + H_2O †	6.4	5E+03	N/A	-	N/A	
		211.1186	$C_9H_{15}O_2N_4$	$C_5H_{10}ON$	Het-CH(CH_2CH_3)-CH_2CONH_2	Ethyl acrylonitrile + H_2O	-1.8	5E+03	-	-	-	
		168.0884	$C_6H_{10}ON_5$	$C_2H_5N_2$	Het-C(CH_2-NH_2)=NH	Glycine nitrile (electrophile)	2.7	2E+03	N/A	-	N/A	

Table S4 Continued:

Pyrimidines	Heterocycle m/z (error ppm) Chemical Formula	Measured Mass	Elemental Composition	Side Chain Composition	Assigned Structure	Candidate Reactant	Mass Error (ppm)	Intensity (A.U.) Net. Spk.	Net. Spk. Rep.	Red. Spk.	Red. Spk. Rep.	# Unique Adducts
**20**	**2-hydroxy pyrimidine**	**141.0662**	**$C_6 H_9 O_2 N_2$**	**$C_2 H_5 O$**	**Het-CH(OH)-CH_3**	**Acetaldehyde**	**2.9**	**4E+06**	**2E+06**	**-**	**-**	**17**
	m/z = 97.0398 (1.4 ppm)	219.0892	$C_{10} H_{11} O_2 N_4$	$C_6 H_7 O N_2$	Het-CH(CH_3)-Het	Acetaldehyde linked dimer*	6.8	-	7E+05	-	-	
	$[C_4 H_5 O N_2]^+$	148.0505	$C_7 H_6 O N_3$	$C_3 H_2 N$	Het-CH=CH-CN	Cyanoacetylene	-0.4	2E+05	3E+03	-	-	
		166.0616	$C_7 H_8 O_2 N_3$	$C_3 H_4 O N$	Het-CH=CH-$CONH_2$	Cyanoacetylene + H_2O	1.8	2E+05	5E+03	-	-	
		138.0666	$C_6 H_8 O N_3$	$C_2 H_4 N$	Het-C(CH_3)=NH	Acetic Acid + NH_3	3.1	1E+05	2E+04	-	-	
		163.0613	$C_7 H_7 O N_4$	$C_3 H_3 N_2$	Het-C(CH_2-CN)=NH / Het-imidazole	Cyanoacetonitrile (electrophile) / Imidazole (nuc. substitution)	-0.7	6E+04	2E+03	-	-	
		183.0771	$C_8 H_{11} O_3 N_2$	$C_4 H_7 O_2$	Het-CH(CH_3)-CH_2-COOH / Het-CH_2-CH(CH_3)-COOH	Crotonitrile + 2 H_2O / Methacrylonitrile + 2 H_2O †	3.8	-	6E+04	-	-	
		124.0509	$C_5 H_6 O N_3$	C H N	Het-CH=NH	Formic Acid + NH_3 †	3.2	-	5E+04	-	-	
		125.0347	$C_5 H_5 O_2 N_2$	C H O	Het-CHO	Formic Acid	0.8	4E+04	1E+03	-	-	
		153.0769	$C_6 H_9 O N_4$	$C_2 H_5 N_2$	Het-C(CH_2-NH_2)=NH	Glycine nitrile (electrophile)	-1.4	4E+04	-	-	-	
		139.0507	$C_6 H_7 O_2 N_2$	$C_2 H_3 O$	Het-CO-CH_3	Acetic Acid	3.3	-	3E+04	-	-	
		168.0771	$C_7 H_{10} O_2 N_3$	$C_3 H_6 O N$	Het-CH_2-CH_2-$CONH_2$	Acrylonitrile + H_2O	2.1	-	3E+04	-	-	
		169.0605	$C_7 H_9 O_3 N_2$	$C_3 H_5 O_2$	Het-CH_2-CH_2-COOH	Acrylonitrile + 2 H_2O	-1.6	-	3E+04	9E+05	3E+04	
		164.0820	$C_8 H_{10} O N_3$	$C_4 H_6 N$	Het-CH(CH_3)-CH_2-CN / Het-CH_2-CH(CH_3)-CN	Crotonitrile / Methacrylonitrile	0.8	2E+04	-	-	-	
		182.0915	$C_8 H_{12} O_2 N_3$	$C_4 H_8 O N$	Het-CH(CH_3)-CH_2-$CONH_2$/ Het-CH_2-CH(CH_3)-$CONH_2$	Crotonitrile + H_2O / Methacrylonitrile + H_2O	-4.8	2E+04	8E+03	7E+05	-	
		136.0504	$C_6 H_6 O N_3$	$C_2 H_2 N$	Het-CH_2-CN	Glycolonitrile	0.5	2E+04	4E+03	2E+04	-	
		169.0717	$C_6 H_9 O_2 N_4$	$C_2 H_5 O N_2$	Het-CH_2-NH-CO-NH_2	Formaldehyde + Urea †	-1.6	2E+04	-	-	-	
		178.0979	$C_9 H_{12} O N_3$	$C_5 H_8 N$	Het-CH(CH_2CH_3)-CH_2-CN	Ethyl-acrylonitrile	3.3	2E+04	-	-	-	
		180.0772	$C_8 H_{10} O_2 N_3$	$C_4 H_6 O N$	Het-C(CH_3)=CH-$CONH_2$	Methylcyanoacetylene + H_2O	2.3	1E+04	-	-	-	
		162.0659	$C_8 H_8 O N_3$	$C_4 H_4 N$	Het-C(CH_3)=CH-CN	Methylcyanoacetylene	-2.2	8E+03	-	4E+04	1E+04	
		229.0813	$C_9 H_{13} O_5 N_2$	$C_5 H_9 O_4$	Het-5-C sugar	5-C sugar condensation	-2.6	5E+03	-	-	-	
		151.0610	$C_6 H_7 O N_4$	$C_2 H_3 N_2$	Het-CH_2-NH-CN	Formaldehyde + Cyanamide †	-3.1	4E+03	-	-	-	
		142.0612	$C_5 H_8 O_2 N_3$	C H_4 O N	Het-CH(OH)-NH_2	Formamide (electrophile) †	0.8	3E+03	-	-	-	
		157.0600	$C_6 H_9 O_3 N_2$	$C_2 H_5 O_2$	Het-CH(OH)-CH_2-OH	Glycolaldehyde	-5.1	2E+03	-	-	-	
		154.0726	$C_5 H_8 O N_5$	C H_4 N_3	Het-NH-CNH-NH_2	Guanidine (nuc. substitution)	2.0	2E+03	-	-	-	
		259.0947	$C_{10} H_{15} O_6 N_2$	$C_6 H_{11} O_5$	Het-6-C sugar	6-C sugar condensation	8.7	1E+03	-	-	-	

Table S4 Continued:

Pyrimidines	Heterocycle m/z (error ppm) Chemical Formula	Measured Mass	Elemental Composition	Side Chain Composition	Assigned Structure	Candidate Reactant	Mass Error (ppm)	Intensity (A.U.)				# Unique Adducts
								Net. Spk.	Net. Spk. Rep.	Red. Spk.	Red. Spk. Rep.	
21	**Uracil** m/z = 113.0353 (7.0 ppm) $[C_4 H_5 O_2 N_2]^+$	**157.0608**	**$C_6 H_9 O_3 N_2$**	**$C H_5 N_2$**	**Het-CH(NH_2)-NH_2**	**Formamidine**	**0.1**	**-**	**7E+04**	**-**	**-**	**11**
		140.0461	$C_5 H_6 O_2 N_3$	C H N	Het-CH=NH	Formic Acid + NH_3 †	2.8	3E+04	1E+04	-	-	
		199.0718	$C_8 H_{11} O_4 N_2$	$C_4 H_7 O_2$	Het-CH(CH_3)-CH_2-COOH / Het-CH_2-CH(CH_3)-COOH	Crotonitrile + 2 H_2O / Methacrylonitrile + 2 H_2O †	2.2	2E+04	-	-	-	
		141.0295	$C_5 H_5 O_3 N_2$	C H O	Het-CHO	Formic Acid	0.5	2E+04	-	-	-	
		157.0244	$C_5 H_5 O_4 N_2$	C H O_2	Het-COOH	HCN + 2 H_2O (nuc. substitution)	0.1	1E+04	-	-	-	
		156.0407	$C_5 H_6 O_3 N_3$	C H_2 O N	Het-$CONH_2$	HCN + H_2O (nuc. substitution)	2.4	1E+04	6E+03	-	-	
		170.0559	$C_6 H_8 O_3 N_3$	$C_2 H_4$ O N	Het-CH_2-$CONH_2$	Glycolonitrile + H_2O †	1.1	5E+03	-	8E+04	-	
		164.0456	$C_7 H_6 O_2 N_3$	$C_3 H_2$ N	Het-CH=CH-CN	Cyanoacetylene	0.8	4E+03	1E+03	-	-	
		154.0618	$C_6 H_8 O_2 N_3$	$C_2 H_4$ N	Het-C(CH_3)=NH	Acetic Acid + NH_3	4.7	4E+03	-	-	-	
		201.0505	$C_7 H_9 O_5 N_2$	$C_3 H_5 O_3$	Het-CO-(CHOH)-CH_2OH	Formic Acid + 2-Carbon sugar	-0.3	4E+03	-	-	-	
		165.0420	$C_6 H_5 O_2 N_4$	C_2 H N_2	Het-C(CN)=NH	Cyanogen	7.8	3E+03	2E+03	-	-	
		185.0545	$C_7 H_9 O_4 N_2$	$C_3 H_5 O_2$	Het-CH_2-CH_2-COOH	Acrylonitrile + 2 H_2O	-6.5	2E+03	-	3E+05	1E+04	
		182.0576	$C_7 H_8 O_3 N_3$	$C_3 H_4$ O N	Het-CH=CH-$CONH_2$	Cyanoacetylene + H_2O	8.6	2E+03	-	-	-	
		180.0770	$C_8 H_{10} O_2 N_3$	$C_4 H_6$ N	Het-CH(CH_3)-CH_2-CN / Het-CH_2-CH(CH_3)-CN	Crotonitrile / Methacrylonitrile	1.4	-	1E+03	-	-	
		152.0458	$C_6 H_6 O_2 N_3$	$C_2 H_2$ N	Het-CH_2-CN	Glycolonitrile	2.2	-	1E+03	-	-	
		194.0865	$C_7 H_8 O_2 N_5$	$C_3 H_4 N_3$	Het-C(CH(NH_2)-CN)=NH	HCN Trimer	6.2	1E+03	-	-	-	
22	**Barbituric Acid** mass = 129 (ND in pos. mode) $[C_4 H_5 O_3 N_2]^+$	**200.0659**	**$C_7 H_{10} O_4 N_3$**	**$C_3 H_6$ O N**	**Het-CH_2-CH_2-$CONH_2$**	**Acrylonitrile + H_2O**	**-3.4**	**1E+03**	**N/A**	**N/A**	**-**	**2**
		183.0526	$C_6 H_7 O_3 N_4$	$C_2 H_3 N_2$	Het-NH-CH_2-CN	Glycine nitrile (nuc. substitution)	7.4	1E+03	N/A	N/A	-	

Table S4 Continued:

Pyrimidines	Heterocycle m/z (error ppm) Chemical Formula	Measured Mass	Elemental Composition	Side Chain Composition	Assigned Structure	Candidate Reactant	Mass Error (ppm)	Intensity (A.U.) Net. Spk.	Net. Spk. Rep.	Red. Spk.	Red. Spk. Rep.	# Unique Adducts
**23**	**Thymine** m/z = 127.0508 (5.2 ppm) $[C_5 H_7 O_2 N_2]^+$	**178.0622**	$\mathbf{C_8 H_8 O_2 N_3}$	$\mathbf{C_3 H_2 N}$	**Het-CH=CH-CN**	**Cyanoacetylene**	**6.4**	**2E+05**	**5E+03**	**-**	**-**	**12**
		154.0620	$C_6 H_8 O_2 N_3$	$C H N$	Het-CH=NH	Formic Acid + NH_3 †	5.8	1E+05	1E+04	-	-	
		184.0722	$C_7 H_{10} O_3 N_3$	$C_2 H_4 O N$	Het-CH_2-$CONH_2$	Glycolonitrile + H_2O †	2.9	5E+04	-	1E+05	-	
		213.0874	$C_9 H_{13} O_4 N_2$	$C_4 H_7 O_2$	Het-CH(CH_3)-CH_2-COOH / Het-CH_2-CH(CH_3)-COOH	Crotonitrile + 2 H_2O / Methacrylonitrile + 2 H_2O †	2.0	4E+04	-	-	-	
		168.0772	$C_7 H_{10} O_2 N_3$	$C_2 H_4 N$	Het-C(CH_3)=NH	Acetic Acid + NH_3	2.8	3E+04	1E+03	-	8E+03	
		192.0781	$C_9 H_{10} O_2 N_3$	$C_4 H_4 N$	Het-C(CH_3)=CH-CN	Methylcyanoacetylene	7.2	2E+04	-	-	-	
		180.0770	$C_8 H_{10} O_2 N_3$	$C_3 H_4 N$	Het-CH_2-CH_2-CN	Acrylonitrile	5.4	3E+03	-	-	-	
		199.0721	$C_8 H_{11} O_4 N_2$	$C_3 H_5 O_2$	Het-CH_2-CH_2-COOH	Acrylonitrile + 2 H_2O	4.0	3E+03	-	2E+05	-	
		195.0766	$C_9 H_{11} O_3 N_2$	$C_4 H_5 O$	Het-C(CH_3)=CH-CHO	Methylpropiolaldehyde	1.0	3E+03	-	-	-	
		215.0774	$C_7 H_{11} O_4 N_4$	$C_2 H_5 O_2 N_2$	Het-NH-CH(NH_2)-COOH	Amino-glycine	1.3	2E+03	-	-	-	
		166.0622	$C_7 H_8 O_2 N_3$	$C_2 H_2 N$	Het-CH_2-CN	Glycolonitrile	6.6	2E+03	-	-	-	
		201.0855	$C_8 H_{13} O_4 N_2$	$C_3 H_7 O_2$	Het-CH_2-(CHOH)-CH_2OH	Hydroxy acetone †	-7.3	2E+03	-	-	-	
		231.0959	$C_9 H_{15} O_5 N_2$	$C_4 H_9 O_3$	Het-CH_2-$(CHOH)_2$-CH_2OH	4-C sugar alcohol condensation	-7.3	1E+03	-	-	-	
		193.0715	$C_8 H_9 O_2 N_4$	$C_3 H_3 N_2$	Het-C(CH_2-CN)=NH	Cyanoacetonitrile †	-2.4	1E+03	-	-	-	
		185.0547	$C_7 H_9 O_4 N_2$	$C_2 H_3 O_2$	Het-CH_2-COOH	Glycolonitrile + 2 H_2O †	-5.3	1E+03	-	-	-	
**24**	**5-hydroxymethyluracil** m/z = 143.0451 (-0.3 ppm) $[C_5 H_7 O_3 N_2]^+$	**156.0776**	$\mathbf{C_6 H_{10} O_2 N_3}$	$\mathbf{C H_4 N}$	**U-CH_2-NH-CH_3**	**(-O) Methylamine**	**5.4**	**3E+05**	**N/A**	**-**	**-**	**10**
		184.0723	$C_7 H_{10} O_3 N_3$	$C_2 H_4 N$	Het-C(CH_3)=NH	Acetic Acid + NH_3	3.2	1E+05	N/A	-	-	
		170.0562	$C_6 H_8 O_3 N_3$	$C H_2 O N$	U-CH_2-$CONH_2$	(-O) HCN + H_2O	1.1	8E+04	N/A	5E+06	1E+07	
		209.0669	$C_8 H_9 O_3 N_4$	$C_3 H_3 O N_2$	U-(CH_2CN)-CH_2-$CONH_2$ / U-(CH_2COHN_2)-CH_2-CN	(-O) HCN + Glycolonitrile + H_2O	-2.5	6E+04	N/A	1E+04	4E+04	
		190.0607	$C_9 H_8 O_2 N_3$	$C_4 H_2 N$	$^+$U(CH_2)-C(CH_3)=CH-CN	(-O) + Methylcyanoacetylene	-2.2	3E+04	N/A	-	-	
		193.0727	$C_8 H_9 O_2 N_4$	$C_3 H_3 N_2$	U-CH_2-imidazole	(-O) Imidazole	3.5	5E+03	N/A	2E+05	-	
		187.0832	$C_6 H_{11} O_3 N_4$	$C H_5 N_2$	Het-CH(NH_2)-NH_2	Formamidine	3.3	2E+03	N/A	-	-	
		223.0823	$C_9 H_{11} O_3 N_4$	$C_4 H_5 O N_2$	U-(CH_2CN)-$(CH_2)_2$$CONH_2$/ U-($CH_2COHN_2$)-$(CH_2)_2$-CN	(-O) HCN + Acrylonitrile + H_2O	-1.0	2E+03	N/A	5E+04	4E+03	
		219.0883	$C_{10} H_{11} O_2 N_4$	$C_5 H_5 N_2$	U(CH_2CN)CH(CH_3)CH_2CN/ U(CH_2CN)CH_2CH(CH_3)-CN	(-O) HCN + Crotonitrile / (-O) HCN + Methacrylonitrile	2.7	1E+03	N/A	-	-	
		203.0568	$C_9 H_7 O_2 N_4$	$C_4 H N_2$	U-(CH_2CN)-CH_2-CH_2-CN	(-O) HCN + Cyanoacetylene	2.2	1E+03	N/A	-	-	
		228.0614	$C_8 H_{10} O_5 N_3$	$C_3 H_4 O_3 N$	U-($CH_2$$CONH_2$)$CH_2$COOH / U-($CH_2$COOH)($CH_2$)$CONH_2$	(-O) HCN + Glycolonitrile + 3 H_2O	-0.5	1E+03	N/A	-	-	

Table S4 Continued:

Triazines	Heterocycle m/z (error ppm) Chemical Formula	Measured Mass	Elemental Composition	Side Chain Composition	Assigned Structure	Candidate Reactant	Mass Error (ppm)	Intensity (A.U.) Net. Spk.	Net. Spk. Rep.	Red. Spk.	Red. Spk. Rep.	# Unique Adducts
**26**	**3-amino-1,2,4-triazine**	**136.0625**	**$C_5H_6N_5$**	**C_2H_2N**	**Het-CH_2-CN**	**Glycolonitrile**	**5.2**	**4E+05**	**1E+05**	**6E+05**	**2E+04**	**24**
	m/z = 97.0513 (4.6 ppm)	139.0617	$C_5H_7ON_4$	C_2H_3O	Het-CO-CH_3	Acetic Acid	1.9	1E+05	4E+05	-	-	
	$[C_3H_5N_4]^+$	112.0623	$C_3H_6N_5$	H_2N	Het-NH_2	Ammonia (nuc. substitution)	4.9	1E+05	6E+04	8E+05	2E+06	
		140.0572	$C_4H_6ON_5$	CH_2ON	Het-$CONH_2$ / Het-NH-CO-NH_2	HCN + H_2O (nuc. substitution) / Nitrosation + Urea	3.5	1E+05	3E+04	7E+06	5E+06	
		201.0976	$C_7H_{13}O_3N_4$	$C_4H_9O_3$	Het-CH_2-$(CHOH)_2$-CH_2OH	4-C sugar alcohol condensation	-3.2	-	6E+04	-	-	
		157.0719	$C_5H_9O_2N_4$	$C_2H_5O_2$	Het-CH(OH)-CH_2-OH	Glycolaldehyde	-0.5	3E+04	1E+05	-	-	
		127.0618	$C_4H_7ON_4$	CH_3O	Het-CH_2-OH	Formaldehyde	2.5	3E+04	7E+03	3E+05	2E+05	
		199.0841	$C_7H_{11}O_3N_4$	$C_4H_7O_3$	Het-4-C sugar	4-C sugar condensation	7.9	2E+04	1E+04	-	-	
		181.0720	$C_7H_9O_2N_4$	$C_4H_5O_2$	Het-$C(CH_3)$=CH-COOH	Methylcyanoacetylene + 2 H_2O	0.0	2E+04	2E+03	-	-	
		155.0680	$C_4H_7ON_6$	CH_3ON_2	Het-NH-$CONH_2$	Urea (nuc. substitution)	2.5	2E+04	3E+03	3E+04	5E+05	
		171.0887	$C_6H_{11}O_2N_4$	$C_3H_7O_2$	Het-CH_2-(CHOH)-CH_2OH	Hydroxy acetone †	5.9	1E+04	-	-	-	
		165.0774	$C_7H_9ON_4$	C_4H_5O	Het-$C(CH_3)$=CH-CHO	Methylpropiolaldehyde	2.0	1E+04	2E+03	-	-	
		153.0771	$C_6H_9ON_4$	C_3H_5O	Het-CH_2-CH_2-CHO	Acrolein	0.3	1E+04	-	-	-	
		164.0931	$C_7H_{10}N_5$	C_4H_6N	Het-$CH(CH_3)$-CH_2-CN / Het-CH_2-$CH(CH_3)$-CN	Crotonitrile / Methacrylonitrile	0.3	1E+04	-	-	-	
		154.0722	$C_5H_8ON_5$	C_2H_4ON	Het-CH_2-$CONH_2$	Glycolonitrile + H_2O †	-0.6	1E+04	6E+03	5E+04	2E+04	
		180.0883	$C_7H_{10}ON_5$	C_4H_6ON	Het-$C(CH_3)$=CH-$CONH_2$	Methylcyanoacetylene + H_2O	1.5	1E+04	-	2E+04	-	
		166.0716	$C_6H_8ON_5$	C_3H_4ON	Het-CH=CH-$CONH_2$	Cyanoacetylene + H_2O	-4.7	-	1E+04	-	-	
		215.0777	$C_7H_{11}O_4N_4$	$C_4H_7O_4$	Het-CO-$(CHOH)_2$-CH_2OH	Formic Acid + 3-Carbon sugar / 4-carbon γ-lactone	1.1	-	1E+04	-	-	
		169.0732	$C_6H_9O_2N_4$	$C_3H_5O_2$	Het-CH_2-CH_2-COOH	Acrylonitrile + 2 H_2O	7.3	8E+03	-	-	-	
		141.0400	$C_4H_5O_2N_4$	CHO_2	Het-COOH / Het-NH-CO-OH	HCN + 2 H_2O (nuc. substitution) / Nitrosation + Urea + H_2O	-5.2	-	8E+03	3E+04	2E+04	
		141.0767	$C_5H_9ON_4$	C_2H_5O	Het-CH(OH)-CH_3	Acetaldehyde	-3.0	-	8E+03	-	-	
		138.0779	$C_5H_8N_5$	C_2H_4N	Het-$C(CH_3)$=NH	Acetic Acid + NH_3	3.5	7E+03	5E+03	1E+04	1E+03	
		185.0673	$C_6H_9O_3N_4$	$C_3H_5O_3$	Het-CO-(CHOH)-CH_2OH	Formic Acid + 2-Carbon sugar	2.0	6E+03	-	2E+03	-	
		151.0616	$C_6H_7ON_4$	C_3H_3O	Het-CH=CH=CHO	Propiolaldehyde	0.8	4E+03	-	1E+05	6E+04	
		167.0925	$C_7H_{11}ON_4$	C_4H_7O	Het-$CH(CH_3)$-CH_2-CHO / Het-CH_2-$CH(CH_3)$-CHO	Crotonaldehyde / Methacrolein	-1.5	-	4E+03	-	-	
		205.0942	$C_7H_9N_8$	$C_4H_5N_4$	Het-CH_2-Het	Formaldehyde linked dimer	-1.1	3E+03	-	2E+05	3E+04	
		155.0564	$C_5H_7O_2N_4$	$C_2H_3O_2$	Het-CH_2-COOH	Glycolonitrile + 2 H_2O †	0.1	2E+03	3E+03	2E+04	-	
		122.0465	$C_4H_4N_5$	CN	Het-CN / Het-NH-CN	HCN (nuc. substitution) / Nitrosation + Cyanamide	2.7	2E+03	-	3E+04	1E+03	
		178.0846	$C_6H_8N_7$	$C_3H_4N_3$	Het-$C(CH(NH_2)$-CN)=NH	HCN trimer	6.1	2E+03	-	5E+05	2E+05	
		162.0785	$C_7H_8N_5$	C_4H_4N	Het-$C(CH_3)$=CH-CN	Methylcyanoacetylene	6.5	2E+03	-	6E+03	-	
		124.0624	$C_4H_6N_5$	CHN	Het-CH=NH / H-Het-CN	Formic Acid + NH_3 / HCN (nuc. addition)	4.2	1E+03	-	6E+04	9E+03	
		215.1127	$C_8H_{15}O_3N_4$	$C_5H_{11}O_3$	Het-$CH_2C(CHOH)_2CH_2OH$	Acrolein + 2 CH_2O	-5.4	-	2E+03	-	-	
		150.0778	$C_6H_8N_5$	C_3H_4N	Het-CH_2-CH_2-CN	Acrylonitrile	2.3	-	1E+03	-	-	
		168.0887	$C_6H_{10}ON_5$	C_3H_6ON	Het-CH_2-CH_2-$CONH_2$	Acrylonitrile + H_2O	4.4	-	1E+03	2E+04	2E+04	
		182.1026	$C_7H_{12}ON_5$	C_4H_8ON	Het-$CH(CH_3)$-CH_2-$CONH_2$/ Het-CH_2-$CH(CH_3)$-$CONH_2$	Crotonitrile + H_2O / Methacrylonitrile + H_2O	-5.7	-	1E+03	-	-	

Table S4 Continued:

Triazines	Heterocycle m/z (error ppm) Chemical Formula	Measured Mass	Elemental Composition	Side Chain Composition	Assigned Structure	Candidate Reactant	Mass Error (ppm)	Intensity (A.U.) Net. Spk.	Net. Spk. Rep.	Red. Spk.	Red. Spk. Rep.	# Unique Adducts
**27**	**Guanazole**	**152.0688**	**$C_4H_6N_7$**	**C_2HN_2**	**Het-C(CN)=NH**	**Cyanogen**	**6.0**	**-**	**1E+07**	**-**	**-**	**7**
	m/z = 100.0622 (3.9 ppm)	128.0572	$C_3H_6ON_5$	CHO	Het-CHO	Formic Acid	3.8	2E+06	3E+05	-	-	
	$[C_2H_6N_5]^+$	127.0736	$C_3H_7N_6$	CHN	Het-CH=NH	Formic Acid + NH_3 †	7.3	-	2E+06	-	-	
		156.1001	$C_4H_{10}N_7$	$C_2H_5N_2$	Het-C(CH_2-NH_2)=NH	Glycine nitrile (electrophile)	5.7	-	2E+05	-	-	
		125.0578	$C_3H_5N_6$	CN	Het-CN	HCN (nuc. substitution) †	6.6	-	1E+05	-	-	
		181.0946	$C_5H_9N_8$	$C_3H_4N_3$	Het-C(CH(NH_2)-CN)=NH	HCN trimer	0.7	2E+03	-	-	-	
		232.1043	$C_7H_{14}O_4N_5$	$C_5H_9O_4$	Het-5-C sugar	5-C sugar condensation	1.1	1E+03	-	-	-	
		262.1129	$C_8H_{16}O_5N_5$	$C_6H_{11}O_5$	Het-6-C sugar	6-C sugar condensation	-6.4	1E+03	-	-	-	
**28**	**Melamine**	**170.0789**	**$C_4H_8ON_7$**	**CH_2ON**	**Het-NH-CO-NH_2**	**Nitrosation + Urea**	**2.3**	**N/A**	**5E+04**	**-**	**N/A**	**1**
	m/z = 127.0724 (-2.0 ppm)	171.0625	$C_4H_7O_2N_6$	CHO_2	Het-NH-CO-OH	Nitrosation + Urea + H_2O	0.0	N/A	1E+04	-	N/A	
	$[C_3H_7N_6]^+$	152.0690	$C_4H_6N_7$	CN	Het-NH-CN	Nitrosation + Cyanamide	6.8	N/A	2E+03	-	N/A	
**Purines**												
**35**	**2-aminopurine**	**180.0889**	**$C_7H_{10}ON_5$**	**C_2H_5O**	**Het-CH(OH)-CH_3**	**Acetaldehyde**	**4.5**	**6E+05**	**1E+05**	**1E+05**	**N/A**	**15**
	m/z = 136.0625 (5.4 ppm)	164.0573	$C_6H_6ON_5$	CHO	Het-CHO	Formic Acid	3.3	3E+05	3E+05	-	**N/A**	
	$[C_5H_6N_5]^+$	205.0842	$C_8H_9ON_6$	C_3H_4ON	Het-CH=CH-$CONH_2$	Cyanoacetylene + H_2O	4.5	2E+05	2E+04	1E+04	N/A	
		180.0994	$C_6H_{10}N_7$	CH_5N_2	Het-CH(NH_2)-NH_2	Formamidine	0.9	1E+05	-	-	N/A	
		189.0894	$C_8H_9N_6$	C_3H_4N	Het-CH_2-CH_2-CN	Acrylonitrile	5.4	8E+04	-	2E+04	N/A	
		254.0884	$C_9H_{12}O_4N_5$	$C_4H_7O_4$	Het-CO-$(CHOH)_2$-CH_2OH	Formic Acid + 3-Carbon sugar / 4-carbon γ-lactone	0.2	7E+04	5E+04	-	N/A	
		207.0995	$C_8H_{11}ON_6$	C_3H_6ON	Het-CH_2-CH_2-$CONH_2$	Acrylonitrile + H_2O	2.7	4E+04	2E+03	6E+04	N/A	
		181.0834	$C_6H_9ON_6$	CH_4ON	Het-CH(OH)-NH_2	Formamide (electrophile) †	0.9	4E+04	-	1E+04	N/A	
		222.0976	$C_9H_{12}O_2N_5$	$C_4H_7O_2$	Het-CH(CH_3)-CH_2-COOH / Het-CH_2-CH(CH_3)- COOH	Crotonitrile + 2 H_2O / Methacrylonitrile + 2 H_2O †	-4.5	1E+04	2E+04	4E+04	N/A	
		204.0869	$C_9H_{10}ON_5$	C_4H_5O	Het-C(CH_3)=CH-CHO	Methylpropiolaldehyde	-5.5	2E+04	-	-	N/A	
		187.0729	$C_8H_7N_6$	C_3H_2N	Het-CH=CH-CN	Cyanoacetylene	1.3	1E+04	3E+03	2E+04	N/A	
		175.0723	$C_7H_7N_6$	C_2H_2N	Het-CH_2-CN	Glycolonitrile	-2.1	1E+04	-	9E+03	N/A	
		196.0831	$C_7H_{10}O_2N_5$	$C_2H_5O_2$	Het-CH(OH)-CH_2-OH	Glycolaldehyde	0.9	6E+03	-	-	N/A	
		208.0941	$C_7H_{10}ON_7$	$C_2H_5ON_2$	Het-CH_2-NH-CO-NH_2	Formaldehyde + Urea †	-0.2	5E+03	-	-	N/A	
		268.1049	$C_{10}H_{14}O_4N_5$	$C_5H_9O_4$	Het-5-C sugar	5-C sugar condensation	3.2	2E+03	-	1E+03	N/A	
		165.0883	$C_6H_9N_6$	CH_4N	Het-NH-CH_3	Methyl amine	0.0	1E+03	1E+03	6E+04	N/A	
		201.0887	$C_9H_9N_6$	C_4H_4N	Het-C(CH_3)=CH-CN	Methylcyanoacetylene	1.9	1E+03	-	-	N/A	

Table S4 Continued:

Purines	Heterocycle<br>m/z (error ppm)<br>Chemical Formula	Measured Mass	Elemental Composition	Side Chain Composition	Assigned Structure	Candidate Reactant	Mass Error (ppm)	Intensity (A.U.)<br>Net. Spk.	Net. Spk. Rep.	Red. Spk.	Red. Spk. Rep.	# Unique Adducts
**36**	**Adenine**<br>m/z = 136.0625 (5.8 ppm)<br>$[C_5H_6N_5]^+$	**254.0900**	$\mathbf{C_9H_{12}O_4N_5}$	$\mathbf{C_4H_7O_4}$	**Het-CO-(CHOH)$_3$-CH$_2$OH**	**Formic Acid + 3-Carbon sugar / 4-carbon γ-lactone**	**6.5**	**1E+06**	**3E+05**	**-**	**-**	**9**
		222.0983	$C_9H_{12}O_2N_5$	$C_4H_7O_2$	Het-CH(CH$_3$)-CH$_2$-COOH / Het-CH$_2$-CH(CH$_3$)-COOH	Crotonitrile + 2 H_2O / Methacrylonitrile + 2 H_2O †	-1.3	7E+04	-	-	-	
		206.1032	$C_9H_{12}ON_5$	C_4H_7O	Het-CH(CH$_3$)-CH$_2$-CHO / Het-CH$_2$-CH(CH$_3$)-CHO	Crotonaldehyde / Methacrolein	-2.1	4E+04	-	-	-	
		164.0572	$C_6H_6ON_5$	CHO	Het-CHO	Formic Acid	3.0	2E+04	2E+04	-	2E+04	
		204.0868	$C_9H_{10}ON_5$	C_4H_5O	Het-C(CH$_3$)=CH-CHO / Het-CH=C(CH$_3$)-CHO	Methylpropiolaldehyde	-5.7	2E+04	-	-	-	
		192.0875	$C_8H_{10}ON_5$	C_3H_5O	Het-CH$_2$-CH$_2$-CHO	Acrolein	-2.8	1E+04	-	-	-	
		268.1030	$C_{10}H_{14}O_4N_5$	$C_5H_9O_4$	Het-5-C sugar	5-C sugar condensation	-4.0	5E+03	-	-	-	
		188.0685	$C_7H_6N_7$	C_2HN_2	Het-C(CN)=NH	Cyanogen	3.2	1E+03	-	-	-	
		206.0673	$C_8H_8O_2N_5$	$C_3H_3O_2$	Het-CH=CH-COOH	Cyanoacetylene + 2 H_2O	0.0	-	1E+03	-	-	
**38**	**Isoguanine**<br>m/z = 152.0577 (6.5 ppm)<br>$[C_5H_6ON_5]^+$	194.0671	$C_7H_8O_2N_5$	C_2H_3O	Het-CO-CH$_3$	Acetic Acid	-0.8	2E+04	N/A	9E+03	N/A	**13**
		180.0521	$C_6H_6O_2N_5$	CHO	Het-CHO	Formic Acid	2.8	2E+04	N/A	-	N/A	
		193.0830	$C_7H_9ON_6$	C_2H_4N	Het-C(CH$_3$)=NH	Acetic Acid + NH_3	-1.5	1E+04	N/A	-	N/A	
		221.0797	$C_8H_9O_2N_6$	C_3H_4ON	Het-CH=CH-CONH$_2$	Cyanoacetylene + H_2O	6.9	1E+04	N/A	-	N/A	
		196.0824	$C_7H_{10}O_2N_5$	C_2H_5O	Het-CH(OH)-CH$_3$	Acetaldehyde	-2.7	9E+03	N/A	-	N/A	
		315.1068	$C_{11}H_{11}O_2N_{10}$	$C_6H_6ON_5$	Het-CH$_2$-Het	Formaldehyde linked dimer	2.2	7E+03	N/A	-	N/A	
		205.0840	$C_8H_9ON_6$	C_3H_4N	Het-CH$_2$-CH$_2$-CN	Acrylonitrile	3.7	6E+03	N/A	2E+04	N/A	
		240.0833	$C_7H_{10}O_3N_7$	$C_2H_5O_2N_2$	Het-NH-CH(NH$_2$)-COOH	Amino-glycine (nuc. substitution)	-3.0	5E+03	N/A	-	N/A	
		204.0640	$C_7H_6ON_7$	C_2HN_2	Het-C(CN)=NH	Cyanogen	5.8	5E+03	N/A	-	N/A	
		210.0989	$C_8H_{12}O_2N_5$	C_3H_7O	Het-C(CH$_3$)$_2$-OH	Acetone	1.9	3E+03	N/A	-	N/A	
		238.0933	$C_9H_{12}O_3N_5$	$C_4H_7O_2$	Het-CH(CH$_3$)-CH$_2$-COOH / Het-CH$_2$-CH(CH$_3$)-COOH	Crotonitrile + 2 H_2O / Methacrylonitrile + 2 H_2O †	-0.5	3E+03	N/A	-	N/A	
		286.1129	$C_{10}H_{16}O_5N_5$	$C_5H_{11}O_4$	Het-CH$_2$-(CHOH)$_3$-CH$_2$OH	5-C sugar alcohol condensation	-5.9	2E+03	N/A	-	N/A	
		182.0667	$C_6H_8O_2N_5$	CH_3O	Het-CH$_2$-OH	Formaldehyde	-2.9	1E+03	N/A	-	N/A	
		225.0721	$C_7H_9O_3N_6$	$C_2H_4O_2N$	Het-CH$_2$-NH-CO-OH	Formaldehyde + Urea + H_2O	-4.1	1E+03	N/A	-	N/A	
		195.0624	$C_6H_7O_2N_6$	CH_2ON	Het-CONH$_2$	HCN + H_2O (nuc. substitution) †	-0.5	1E+03	N/A	-	N/A	

Table S4 Continued:

Purines	Heterocycle m/z (error ppm) Chemical Formula	Measured Mass	Elemental Composition	Side Chain Composition	Assigned Structure	Candidate Reactant	Mass Error (ppm)	Intensity (A.U.) Net. Spk.	Net. Spk. Rep.	Red. Spk.	Red. Spk. Rep.	# Unique Adducts
**39**	**Guanine** m/z = 152.0572 (3.6 ppm) $[C_5H_6ON_5]^+$	**196.0935**	**$C_6H_{10}ON_7$**	**CH_5N_2**	**Het-CH(NH_2)-NH_2**	**Formamidine**	**-3.1**	**4E+03**	**3E+04**	**N/A**	**-**	**7**
		179.0677	$C_6H_7ON_6$	HCN	Het-CH=NH	Formic Acid + NH_3 †	0.9	-	2E+04	N/A	-	
		191.0692	$C_7H_7ON_6$	C_2H_2N	Het-CH_2-CN	Glycolonitrile	8.2	7E+03	-	N/A	-	
		236.0780	$C_9H_{10}O_3N_5$	$C_4H_5O_2$	Het-C(CH_3)=CH-COOH	Methylcyanoacetylene + 2 H_2O	0.8	4E+03	-	N/A	-	
		204.0620	$C_7H_6ON_7$	C_2HN_2	Het-C(CN)=NH	Cyanogen	-4.0	4E+03	-	N/A	-	
		220.0816	$C_9H_{10}O_2N_5$	C_4H_5O	Het-C(CH_3)=CH-CHO	Methylpropiolaldehyde	-5.9	2E+03	-	N/A	-	
		208.0835	$C_8H_{10}O_2N_5$	C_3H_5O	Het-CH_2-CH_2-CHO	Acrolein	2.9	1E+03	-	N/A	1E+03	
		180.0520	$C_6H_6O_2N_5$	CHO	Het-CHO	Formic Acid	2.2	1E+03	-	N/A	-	

Table S5. Adducts formed from select N-heterocycles incubated with candidate spark organics in isolated reactions. N-heterocycles (1 mM) were either incubated with various organics (1 mM) for 72 hours at 5, 30, or 80 °C or were immediately frozen and stored at -80 °C until analysis. Intensities of each ion are given in arbitrary units (A.U.).

Heterocycle	Reactant:	Temperature	Measured Mass	Theoretical Mass	Mass Error (ppm)	Intensity (A.U.)	Elemental Composition	Assigned Structure (Het = Heterocycle)
**(1) 2-aminopyridine**	Glycolonitrile	Frozen	*Target Adduct Not Detected Above Thresholds*					
		30 °C	*Target Adduct Not Detected Above Thresholds*					
		80 °C	134.0717	134.0713	3.0	2E+04	$C_7 H_8 N_3$	Het-CH_2-CN + H^+
			152.0817	152.0818	-0.9	1E+04	$C_7 H_{10} O N_3$	Het-CH_2-$CONH_2$ + H^+
	Acrylonitrile	Frozen	148.0870	148.0869	0.6	1E+04	$C_8 H_{10} N_3$	Het-CH_2-CH_2-CN + H^+
		80 °C	167.0825	167.0815	5.8	8E+07	$C_8 H_{11} O_2 N_2$	Het-CH_2-CH_2-COOH + H^+
			148.0878	148.0869	5.6	5E+05	$C_8 H_{10} N_3$	Het-CH_2-CH_2-CN + H^+
	Acrylic Acid	Frozen	*Target Adduct Not Detected Above Thresholds*					
		80 °C	167.0818	167.0815	1.5	7E+04	$C_8 H_{11} O_2 N_2$	Het-CH_2-CH_2-COOH + H^+
	Acrylamide	Frozen	*Target Adduct Not Detected Above Thresholds*					
		80 °C	167.0819	167.0815	2.6	6E+05	$C_8 H_{11} O_2 N_2$	Het-CH_2-CH_2-COOH + H^+
	Crotonitrile	80 °C	162.1026	162.1026	-1.8	2E+03	$C_9 H_{12} N_3$	Het-CH(CH_3)-CH_2-CN + H^+
	Propiolamide	Frozen	*Target Adduct Not Detected Above Thresholds*					
		80 °C	*Target Adduct Not Detected Above Thresholds*					
	Propiolic Acid	Frozen	*Target Adduct Not Detected Above Thresholds*					
		80 °C	165.0662	165.0659	2.4	2E+04	$C_8 H_9 O_2 N_2$	Het-CH=CH-COOH + H^+
	2-butynoic acid	Frozen	*Target Adduct Not Detected Above Thresholds*					
		80 °C	179.0810	179.0815	-2.9	4E+03	$C_9 H_{11} O_2 N_2$	Het-C(CH_3)=CH-COOH + H^+
**(3) 3-aminopyridine**	Glycolonitrile	Frozen	134.0713	134.0713	0.2	1E+04	$C_7 H_8 N_3$	Het-CH_2-CN + H^+
		80 °C	152.0827	152.0818	5.6	1E+07	$C_7 H_{10} O N_3$	Het-CH_2-$CONH_2$ + H^+
			153.0666	153.0659	5.1	3E+05	$C_7 H_9 O_2 N_2$	Het-CH_2-COOH + H^+
			134.0717	134.0713	3.6	5E+03	$C_7 H_8 N_3$	Het-CH_2-CN + H^+
	Acrylonitrile	Frozen	*Target Adduct Not Detected Above Thresholds*					
		80 °C	148.0867	148.0869	-1.4	2E+04	$C_8 H_{10} N_3$	Het-CH_2-CH_2-CN + H^+
	Propiolic Acid	Frozen	*Target Adduct Not Detected Above Thresholds*					
		80 °C	165.0668	165.0659	5.9	5E+05	$C_8 H_9 O_2 N_2$	Het-CH=CH-COOH + H^+
**(15) 2,4-diaminopyrimidine**	Propiolamide	30 °C	180.0890	180.0880	5.5	2E+05	$C_7 H_{10} O N_5$	Het-CH=CH-$CONH_2$ + H^+
			181.0718	181.0720	-1.0	1E+04	$C_7 H_9 O_2 N_4$	Het-CH=CH-COOH + H^+
		80 °C	180.0884	180.0880	2.2	7E+04	$C_7 H_{10} O N_5$	Het-CH=CH-$CONH_2$ + H^+
			181.0712	181.0720	-4.5	6E+03	$C_7 H_9 O_2 N_4$	Het-CH=CH-COOH + H^+

Table S5 Continued:

Heterocycle	Reactant:	Temperature	Measured Mass	Theoretical Mass	Mass Error (ppm)	Intensity (A.U.)	Elemental Composition	Assigned Structure (Het = Heterocycle)
**(16) 2,4,6-triaminopyrimidine**	Formic Acid	Frozen	154.0726	154.0724	1.7	1E+05	$C_5 H_8 O N_5$	Het-CHO + H^+
		80 °C	154.0722	154.0724	-0.6	1E+05	$C_5 H_8 O N_5$	Het-CHO + H^+
	Glycolonitrile	80 °C	183.0999	183.0989	5.6	2E+06	$C_6 H_{11} O N_6$	Het-CH_2-$CONH_2$ + H^+
			165.0890	165.0883	4.2	1E+06	$C_6 H_9 N_6$	Het-CH_2-CN + H^+
			184.0827	184.0829	-1.1	6E+03	$C_6 H_{10} O_2 N_5$	Het-CH_2-COOH + H^+
	2-butynoic acid	30 °C	*Target Adduct Not Detected Above Thresholds*					
		80 °C	210.0984	210.0986	-0.7	1E+03	$C_8 H_{12} O_2 N_5$	Het-C(CH_3)=CH-COOH + H^+
**(17) Cytosine**	Glycolonitrile	Frozen	*Target Adduct Not Detected Above Thresholds (Note: adduct not detected in either reducing or neutral spark mixture)*					
		80 °C	*Target Adduct Not Detected Above Thresholds (Note: adduct not detected in either reducing or neutral spark mixture)*					
	Acrylic Acid	Frozen	184.0730	184.0717	7.4	2E+05	$C_7 H_{10} O_3 N_3$	Het-CH_2-CH_2-COOH + H^+
		80 °C	184.0725	184.0717	4.3	3E+03	$C_7 H_{10} O_3 N_3$	Het-CH_2-CH_2-COOH + H^+
	Propiolic Acid	Frozen	182.0569	182.0560	4.9	4E+04	$C_7 H_8 O_3 N_3$	Het-CH=CH-COOH + H^+
		80 °C	*Target Adduct Not Detected Above Thresholds*					
**(18) Isocytosine**	Acrylic Acid	Frozen	*Target Adduct Not Detected Above Thresholds*					
		80 °C	184.0714	184.0717	-1.2	1E+05	$C_7 H_{10} O_3 N_3$	Het-CH_2-CH_2-COOH + H^+
	Propiolamide	30 °C	*Target Adduct Not Detected Above Thresholds*					
		80 °C	181.0725	181.0720	2.5	3E+04	$C_7 H_9 O_2 N_4$	Het-CH=CH-$CONH_2$ + H^+
			182.0554	182.0560	-3.6	2E+03	$C_7 H_8 O_3 N_3$	Het-CH=CH-COOH + H^+
**(20) 2-hydroxypyrimidine**	Crotonitrile	80 °C	164.0819	164.0818	0.6	1E+05	$C_8 H_{10} O N_3$	Het-CH(CH_3)-CH_2-CN + H^+
			182.0930	182.0924	3.3	3E+03	$C_8 H_{12} O_2 N_3$	Het-CH(CH_3)-CH_2-$CONH_2$ + H^+
**(21) Uracil**	Formic Acid	Frozen	141.0301	141.0295	4.5	1E+03	$C_5 H_5 O_3 N_2$	Het-CHO + H^+
		80 °C	*Target Adduct Not Detected Above Thresholds*					
	Glycolonitrile:	Frozen	*Target Adduct Not Detected Above Thresholds*					
		30 °C	170.0570	170.0560	5.7	2E+03	$C_6 H_8 O_3 N_3$	Het-CH_2-$CONH_2$ + H^+
		80 °C	170.0566	170.0560	3.2	3E+04	$C_6 H_8 O_3 N_3$	Het-CH_2-$CONH_2$ + H^+
			152.0453	152.0455	-1.1	1E+03	$C_6 H_6 O_2 N_3$	Het-CH_2-CN + H^+
	Propiolaldehyde:	5 °C	167.0459	167.0451	4.4	5E+04	$C_7 H_7 O_3 N_2$	Het-CH=CH-CHO + H^+
		80 °C	*Target Adduct Not Detected Above Thresholds*					
	Acrylonitrile:	Frozen	185.0552	185.0557	-2.4	6E+03	$C_7 H_9 O_4 N_2$	Het-CH_2-CH_2-COOH + H^+
			184.0708	184.0717	-4.9	6E+03	$C_7 H_{10} O_3 N_3$	Het-CH_2-CH_2-$CONH_2$ + H^+
		30 °C	166.0622	166.0611	6.5	1E+06	$C_7 H_8 O_2 N_3$	Het-CH_2-CH_2-CN + H^+
			184.0704	184.0717	-6.7	1E+04	$C_7 H_{10} O_3 N_3$	Het-CH_2-CH_2-$CONH_2$ + H^+
		80 °C	184.0725	184.0717	4.7	4E+07	$C_7 H_{10} O_3 N_3$	Het-CH_2-CH_2-$CONH_2$ + H^+
			166.0619	166.0611	4.9	7E+06	$C_7 H_8 O_2 N_3$	Het-CH_2-CH_2-CN + H^+
			185.0564	185.0557	3.9	3E+06	$C_7 H_9 O_4 N_2$	Het-CH_2-CH_2-COOH + H^+
	Acrylic Acid:	Frozen	185.0567	185.0557	5.5	8E+06	$C_7 H_9 O_4 N_2$	Het-CH_2-CH_2-COOH + H^+
		30 °C	185.0572	185.0557	8.3	3E+06	$C_7 H_9 O_4 N_2$	Het-CH_2-CH_2-COOH + H^+
		80 °C	185.0572	185.0557	8.3	2E+06	$C_7 H_9 O_4 N_2$	Het-CH_2-CH_2-COOH + H^+
	Crotonitrile	80 °C	180.0772	180.0768	2.4	2E+05	$C_8 H_{10} O_2 N_3$	Het-CH(CH_3)-CH_2-CN + H^+
			198.0869	198.0873	-2.1	1E+04	$C_8 H_{12} O_3 N_3$	Het-CH(CH_3)-CH_2-$CONH_2$ + H^+
	Propiolic Acid	Frozen	183.0400	183.0401	-0.4	5E+04	$C_7 H_7 O_4 N_2$	Het-CH=CH-COOH + H^+
		30 °C	183.0403	183.0401	1.7	3E+04	$C_7 H_7 O_4 N_2$	Het-CH=CH-COOH + H^+
		80 °C	*Target Adduct Not Detected Above Thresholds*					

Table S5 Continued:

Heterocycle	Reactant:	Temperature	Measured Mass	Theoretical Mass	Mass Error (ppm)	Intensity (A.U.)	Elemental Composition	Assigned Structure (Het = Heterocycle)
**(23) Thymine**	Glycolonitrile:	Frozen	*Target Adduct Not Detected Above Thresholds (Note: Adduct detected in reducing and neutral spark mixtures)*					
		30 °C	*Target Adduct Not Detected Above Thresholds*					
		80 °C	*Target Adduct Not Detected Above Thresholds*					
	Acrylic Acid	Frozen	199.0721	199.0713	4.1	3E+05	$C_8 H_{11} O_4 N_2$	Het-CH_2-CH_2-COOH + H^+
		80 °C	199.0732	199.0713	9.6	2E+06	$C_8 H_{11} O_4 N_2$	Het-CH_2-CH_2-COOH + H^+
	Propiolic Acid	Frozen	197.0567	197.0557	5.1	1E+05	$C_8 H_9 O_4 N_2$	Het-CH=CH-COOH + H^+
		80 °C	*Target Adduct Not Detected Above Thresholds*					
**1,3-dimethyl uracil**	Glycolonitrile	80 °C	*Target Adduct Not Detected Above Thresholds*					
	Acrylic Acid	80 °C	*Target Adduct Not Detected Above Thresholds*					
	Propiolic Acid	Frozen	*Target Adduct Not Detected Above Thresholds*					
		30 °C	*Target Adduct Not Detected Above Thresholds*					
**3,5-dimethyl uracil**	Glycolonitrile	80 °C	*Target Adduct Not Detected Above Thresholds*					
	Acrylic Acid	80 °C	213.0879	213.0870	4.3	8E+06	$C_9 H_{13} O_4 N_2$	Het-CH_2-CH_2-COOH + H^+
	Propiolic Acid	Frozen	211.0726	211.0713	5.8	7E+06	$C_9 H_{11} O_4 N_2$	Het-CH=CH-COOH + H^+
		30 °C	211.0723	211.0713	4.8	7E+06	$C_9 H_{11} O_4 N_2$	Het-CH=CH-COOH + H^+
**(24) 5-hydroxymethyluracil (5-HMU)**	Glycolonitrile	Frozen	*Target Adduct Not Detected Above Thresholds*					
		80 °C	200.0658	200.0666	-4.1	2E+04	$C_7 H_{10} O_4 N_3$	5HMU-CH_2-$CONH_2$ + H^+
	Acrylonitrile	Frozen	*Target Adduct Not Detected Above Thresholds*					
		80 °C	196.0710	196.0717	-3.5	4E+04	$C_8 H_{10} O_3 N_3$	5HMU-CH_2CH_2CN + H^+
	NaCN	80 °C	170.0569	170.0560	5.2	8E+05	$C_6 H_8 O_3 N_3$	$CONH_2$-CH_2-U + H^+
			152.0456	152.0454	1.3	4E+04	$C_6 H_6 O_2 N_3$	CN-CH_2-U + H^+
	NaCN + Acrylonitrile	80 °C	196.0726	196.0717	4.5	1E+06	$C_8 H_{10} O_3 N_3$	5HMU-CH_2CH_2CN + H^+
			223.0827	223.0826	0.8	4E+04	$C_9 H_{11} O_3 N_4$	CN-CH_2-U-CH_2-CH_2-$CONH_2$ + H^+
			224.0663	224.0666	-1.5	2E+04	$C_9 H_{10} O_4 N_3$	CN-CH_2-U-CH_2-CH_2-COOH + H^+
	Propiolic Acid	Frozen	195.0414	195.0401	6.8	5E+03	$C_8 H_7 O_4N_2$	CH_2=U^+-CH=CH-COOH
		80 °C	*Target Adduct Not Detected Above Thresholds*					
**(26) 3-amino-1,2,4-triazine**	NaCN	Frozen	191.0792	191.0788	2.0	3E+07	$C_6 H_7 N_8$	Het-Het + H^+
			140.0572	140.0567	3.7	2E+06	$C_4 H_6 O N_5$	Het-$CONH_2$ + H^+
			122.0459	122.0461	-1.7	1E+04	$C_4 H_4 N_5$	Het-CN + H^+
			124.0625	124.0618	5.5	6E+03	$C_4 H_6 N_5$	H-Het-CN + H^+
			178.0829	178.0836	-3.7	1E+03	$C_6 H_8 N_7$	Het-C(CH(NH_2)-CN)=NH + H^+ (from HCN trimer)
		30 °C	191.0797	191.0788	4.7	2E+07	$C_6 H_7 N_8$	Het-Het + H^+
			140.0574	140.0567	5.5	5E+06	$C_4 H_6 O N_5$	Het-$CONH_2$ + H^+
			141.0404	141.0407	-2.4	6E+03	$C_4 H_5 O_2 N_4$	Het-COOH + H^+
			178.0829	178.0836	-3.5	3E+03	$C_6 H_8 N_7$	Het-C(CH(NH_2)-CN)=NH + H^+ (from HCN trimer)
		80 °C	191.0788	191.0788	0.1	7E+04	$C_6 H_7 N_8$	Het-Het + H^+
			141.0414	141.0407	5.2	8E+03	$C_4 H_5 O_2 N_4$	Het-COOH + H^+
	Glycolonitrile	80 °C	136.0623	136.0618	3.6	2E+04	$C_5 H_6 N_5$	Het-CH_2-CN + H^+
			155.0571	155.0564	4.6	4E+03	$C_5 H_7 O_2 N_4$	Het-CH_2-COOH + H^+
			154.0729	154.0723	3.4	2E+03	$C_5 H_8 O N_5$	Het-CH_2-$CONH_2$ + H^+
	2-butynoic Acid	30 °C	*Target Adduct Not Detected Above Thresholds*					
		80 °C	*Target Adduct Not Detected Above Thresholds*					

Table S5 Continued:

Heterocycle	Reactant:	Temperature	Measured Mass	Theoretical Mass	Mass Error (ppm)	Intensity (A.U.)	Elemental Composition	Assigned Structure (Het = Heterocycle)
**(27) Guanazole**	Formic Acid	Frozen	128.0569	128.0567	1.5	3E+05	$C_3 H_6 O N_5$	Het-CHO + H^+
		80 °C	128.0571	128.0567	3.5	7E+05	$C_3 H_6 O N_5$	Het-CHO + H^+
	Glycolonitrile	30 °C	158.0672	158.0673	-0.4	4E+04	$C_4 H_8 O_2 N_5$	Het-CH_2-COOH + H^+
			157.0824	157.0832	-5.6	3E+04	$C_4 H_9 O N_6$	Het-CH_2-$CONH_2$ + H^+
		80 °C	157.0831	157.0832	3.3	3E+05	$C_4 H_9 O N_6$	Het-CH_2-$CONH_2$ + H^+
			158.0676	158.0673	2.1	6E+04	$C_4 H_8 O_2 N_5$	Het-CH_2-COOH + H^+
	Crotonitrile	80 °C	167.1045	167.1040	3.4	1E+06	$C_6 H_{11} N_6$	Het-C(CH_3)-CN + H^+
			185.1144	185.1146	-0.7	9E+03	$C_6 H_{13} O N_6$	Het-C(CH_3)-$CONH_2$ + H^+
			186.0986	186.0986	-2.6	3E+03	$C_6 H_{12} O_2 N_5$	Het-C(CH_3)-COOH + H^+
**(35) 2-aminopurine**	Propiolamide	30 °C	205.0844	205.0832	5.6	5E+05	$C_8 H_9 O N_6$	Het-CH=CH-$CONH_2$ + H^+
			206.0662	206.0673	-5.3	4E+03	$C_8 H_8 O_2 N_5$	Het-CH=CH-COOH + H^+
		80 °C	205.0836	205.0832	1.9	1E+04	$C_8 H_9 O N_6$	Het-CH=CH-$CONH_2$ + H^+
			206.0683	206.0673	5.0	2E+03	$C_8 H_8 O_2 N_5$	Het-CH=CH-COOH + H^+
**(36) Adenine**	1 mM Formic Acid	Frozen	*Target Adduct Not Detected Above Thresholds*					
		80 °C	164.0558	164.0567	-5.5	7E+03	$C_6 H_6 O N_5$	Het-CHO + H^+
	10 mM Formic Acid	Frozen	164.0573	164.0567	3.8	2E+05	$C_6 H_6 O N_5$	Het-CHO + H^+
		80 °C	164.0576	164.0567	5.3	2E+05	$C_6 H_6 O N_5$	Het-CHO + H^+
	Glycolonitrile	Frozen	*Target Adduct Not Detected Above Thresholds*					
		30 °C	*Target Adduct Not Detected Above Thresholds*					
		80 °C	193.0830	193.0832	-1.3	1E+04	$C_7 H_9 O N_6$	Het-CH_2-$CONH_2$ + H^+
			194.0684	194.0673	6.0	2E+03	$C_7 H_8 O_2 N_5$	Het-CH_2-COOH + H^+
	Propiolaldehyde	5 °C	190.0736	190.0723	6.7	1E+06	$C_8 H_8 O N_5$	Het-CH=CH-CHO + H^+
		80 °C	190.0731	190.0723	4.1	1E+05	$C_8 H_8 O N_5$	Het-CH=CH-CHO + H^+
	Acrylonitrile	Frozen	189.0880	189.0883	-2.0	2E+04	$C_8 H_9 N_6$	Het-CH_2-CH_2-CN + H^+
		30 °C	189.0897	189.0883	7.5	1E+06	$C_8 H_9 N_6$	Het-CH_2-CH_2-CN + H^+
			207.1001	207.0989	5.8	3E+04	$C_8 H_{11} O N_6$	Het-CH_2-CH_2-$CONH_2$ + H^+
		80 °C	207.1001	207.0989	5.8	3E+07	$C_8 H_{11}O N_6$	Het-CH_2-CH_2-$CONH_2$ + H^+
			208.0841	208.0829	5.9	2E+07	$C_8 H_{10} O_2 N_5$	Het-CH_2-CH_2-COOH + H^+
			189.0894	189.0883	5.8	9E+05	$C_8 H_9 N_6$	Het-CH_2-CH_2-CN + H^+
	Acrylic Acid	Frozen	208.0841	208.0829	5.8	2E+06	$C_8 H_{10} O_2 N_5$	Het-CH_2-CH_2-COOH + H^+
		30 °C	208.0841	208.0829	5.7	1E+06	$C_8 H_{10} O_2 N_5$	Het-CH_2-CH_2-COOH + H^+
		80 °C	208.0831	208.0829	0.9	2E+07	$C_8 H_{10} O_2 N_5$	Het-CH_2-CH_2-COOH + H^+
	Crotonitrile	80 °C	203.1039	203.1040	-0.3	7E+05	$C_9 H_{11} N_6$	Het-CH(CH_3)-CH_2-CN + H^+
			221.1156	221.1146	4.6	7E+05	$C_9 H_{13} O N_6$	Het-CH(CH_3)-CH_2-$CONH_2$ + H^+
			222.0995	222.0986	4.5	1E+03	$C_9 H_{12} O_2 N_5$	Het-CH(CH_3)-CH_2-COOH + H^+
	Propiolic Acid	Frozen	206.0680	206.0673	3.8	9E+05	$C_8 H_8O_2N_5$	Het-CH=CH-COOH + H^+
		30 °C	206.0678	206.0673	2.7	6E+05	$C_8 H_8O_2N_5$	Het-CH=CH-COOH + H^+
		80 °C	*Target Adduct Not Detected Above Thresholds*					

Table S6. A detailed summary of the adducts made by N-heterocycles incubated with Miller-Urey spark discharge mixtures generated under reducing and neutral atmospheres. The adducts are grouped (colored background) by the chemistry of the side chain (i.e., electrophilic carbonyl, alcohol, sugar, nucleophile, or nucleophile/electrophilic carbonyl). In general, in each group the adducts are ordered starting with those produced by the most N-heterocycles. Note that hydrolysis derivatives are grouped. Major adducts (i.e., those formed by 10 or more N-heterocycles) are bolded and correspond to reactions shown in Fig. 3 (main text).

			Reducing Atmosphere		Neutral Atmosphere	
**Group**	**Predicted Reactant<br>nuc. (nucleophilic)**	**Assigned Structure<br>Het = heterocycle<br>X = CN, $CONH_2$, or COOH**	**# Heterocycles that formed adduct**	**N-heterocycles that formed the adduct**	**# Heterocycles that formed adduct**	**N-heterocycles that formed the adduct**
**Electrophilic Carbonyl**	**Glycolonitrile /<br>Glycolonitrile + H_2O /<br>Glycolonitrile + 2 H_2O**	**Het-CH_2-X**	**27**	**# 1, 2, 3, 9, 15, 16, 20, 21, 23, 24, 26, 27, 28, 30, 32, 33, 35, 36, 37, 40, 41, 42, 43, 46, 47, 50, 52**	**12**	**# 1, 3, 15, 16, 18, 20, 21, 23, 24, 26, 35, 39**
**Electrophilic Carbonyl**	**Acrylonitrile/amide/acid**	**Het-CH_2-CH_2-X**	**19**	**# 1, 3, 15, 19, 20, 21, 23, 24, 26, 27, 32, 33, 35, 36, 38, 42, 43, 47, 48**	**14**	**# 1, 8, 9, 15, 17, 18, 20, 21, 22, 23, 24, 26, 35, 38**
**Electrophilic Carbonyl**	**Methylcyanoacetylene /<br>Methylcyanoacetylene + H_2O /<br>Methylcyanoacetylene + 2 H_2O**	**Het-C(CH_3)=CH-X**	**16**	**# 1, 2, 3, 15, 16, 18, 20, 24, 26, 27, 30, 32, 35, 36, 39, 47**	**10**	**# 1, 3, 8, 16, 20, 23, 24, 26, 35, 39**
**Electrophilic Carbonyl**	**Propiolaldehyde**	**Het-CH=CH=CHO**	**14**	**# 15, 16, 18, 21, 23, 24, 26, 29, 33, 37, 38, 42, 47, 49**	**1**	**# 26**
**Electrophilic Carbonyl**	**Cyanoacetylene /<br>Cyanoacetylene + H_2O /<br>Cyanoacetylene + 2 H_2O**	**Het-CH=CH-X**	**13**	**# 1, 2, 3, 16, 24, 26, 27, 30, 32, 33, 35, 37, 47**	**14**	**# 1, 3, 15, 16, 17, 18, 20, 21, 23, 24, 26, 35, 36, 38**
**Electrophilic Carbonyl**	**Methylpropiolaldehyde**	**Het-C(CH_3)=CH-CHO**	**12**	**# 15, 16, 21, 27, 30, 32, 37, 38, 44, 47, 48, 49**	**7**	**# 1, 8, 23, 26, 35, 36, 39**
**Electrophilic Carbonyl**	**HCN (nuc. substitiution) or<br>HCN (nuc. addition)**	**Het-X or<br>H-Het-X**	**10**	**# 4, 24, 26, 31, 33, 46, 47, 48, 49, 52**	**10**<br>(3 of which likely from HCN)	**# 21, 24, 26**<br>(Hets that formed same Δ *m/z*, but via nitrosation are listed under Nucleophiles)
**Electrophilic Carbonyl**	**Formic Acid /<br>Formic Acid + NH_3 (schiff base)**	**Het-CHO /<br>Het-CH=NH**	**5**	**# 16, 24, 26, 30, 36**	**13**	**# 1, 3, 15, 16, 20, 21, 26, 23, 27, 35, 36, 38, 39**
**Electrophilic Carbonyl**	**Crotonitrile/amide/acid or<br>Methacrylonitrile/amide/acid**	**Het-CH(CH_3)-CH_2-X /<br>Het-CH_2-CH(CH_3)-X**	**10**	**# 1, 2, 3, 16, 20, 24, 27, 32, 35, 47**	**14**	**# 1, 9, 15, 16, 17, 18, 20, 21, 23, 24, 26, 35, 36, 38**
**Electrophilic Carbonyl**	**Acrolein**	**Het-CH_2-CH_2-CHO**	**10**	**# 16, 27, 30, 32, 37, 39, 46, 47, 48, 52**	**4**	**# 1, 26, 36, 39**
**Electrophilic Carbonyl**	**Acetic Acid /<br>Acetic Acid + NH_2 (schiff base)**	**Het-CO-CH_3 /<br>Het-CNH-CH_3**	**7**	**# 26, 30, 35, 37, 38, 46, 47**	**12**	**# 1, 3, 9, 16, 17, 18, 20, 21, 23, 24, 26, 38**
Electrophilic Carbonyl	Crotonaldehyde /<br>Methacrylonitrile	Het-CH(CH_3)-CH_2-CHO /<br>Het-CH_2-CH(CH_3)-CHO	1	# 47	3	# 1, 26, 36
Electrophilic Carbonyl	Ethyl-acrylonitrile/amide/acid	Het-CH(CH_2CH_3)-CH_2-CN	5	# 1, 30, 32, 35, 47	4	# 1, 15, 17, 20
Electrophilic Carbonyl	Alanine	Het-NH-CH(CH_3)-COOH	2	# 26, 47	0	ND

Table S6 Continued:

Group	Predicted Reactant	Assigned Structure<br>Het = heterocycle<br>X = CN, $CONH_2$, or COOH	Reducing Atmosphere: # Heterocycles that formed adduct	Reducing Atmosphere: N-heterocycles that formed the adduct	Neutral Atmosphere: # Heterocycles that formed adduct	Neutral Atmosphere: N-heterocycles that formed the adduct
**Alcohol**	**Acetaldehyde**	**Het-CH(OH)-CH_3**	**3**	**# 32, 35, 39**	**11**	**# 1, 3, 8, 9, 15, 16, 18, 20, 26, 35, 38**
Alcohol	Formaldehyde /<br>Formaldehyde linked dimer	Het-CH_2-OH	7	# 1, 2, 4, 15, 21, 26, 35	3	# 1, 26, 38
Alcohol	Acetone	Het-C$(CH_3)_2$-OH	1	# 35	1	# 38
Alcohol	Hydroxy acetone	Het-COH(CH_3)-CH_2OH	3	# 21, 35, 47	5	# 7, 15, 16, 23, 26
Alcohol	Glycolaldehyde<br>(electrophile)	Het-CH(OH)-CH_2OH	1	# 47	5	# 1, 16, 20, 26, 35
Alcohol	3-C sugar<br>(electrophile)	Het-$(CHOH)_2$-CH_2OH	1	# 35	0	ND
Alcohol	4-C sugar<br>(electrophile)	Het-$(CHOH)_3$-CH_2OH	0	ND	1	# 15
Alcohol	5-C sugar<br>(electrophile)	Het-$(CHOH)_4$-CH_2OH	1	# 40	0	ND
Alcohol	6-C sugar<br>(electrophile)	Het-$(CHOH)_5$-CH_2OH	2	# 24, 26	0	ND
Alcohol	4-C sugar alcohol condensation	Het-CH_2-$(CHOH)_2$-CH_2OH	0	ND	3	# 8, 23, 26
Alcohol	5-C sugar alcohol condensation	Het-CH_2-$(CHOH)_3$-CH_2OH	0	ND	1	# 38
Alcohol	6-C sugar alcohol condensation	Het-CH_2-$(CHOH)_4$-CH_2OH	1	# 17	0	ND
Sugar	5-C sugar condensation	Het-5-C sugar	2	# 15, 35	5	# 15, 20, 27, 35, 36
Sugar	6-C sugar condensation	Het-6-C sugar	1	# 41	2	# 20, 27
Sugar	4-C sugar condensation	Het-4-C sugar	0	ND	1	# 26
Sugar	Formic Acid + 3-Carbon sugar /<br>4-carbon γ-lactone	Het-CO-$(CHOH)_2$-CH_2OH	2	# 16, 26	4	# 15, 26, 35, 36
Sugar	Formic Acid + 4-Carbon sugar /<br>5-carbon γ-lactone	Het-CO-$(CHOH)_3$-CH_2OH	0	ND	2	# 3, 15
Sugar	Formic Acid + 2-Carbon sugar	Het-CO-(CHOH)-CH_2OH	1	# 26	2	# 21, 26
Sugar	Acrolein + 2 Formaldehyde	Het-CH_2-C$(CH_2OH)_2$-CHO	1	# 41	1	# 26

Table S6 Continued:

Group	Predicted Reactant nuc. (nucleophilic)	Assigned Structure Het = heterocycle X = CN, $CONH_2$, or COOH	Reducing Atmosphere: # Heterocycles that formed adduct	Reducing Atmosphere: N-heterocycles that formed the adduct	Neutral Atmosphere: # Heterocycles that formed adduct	Neutral Atmosphere: N-heterocycles that formed the adduct
**Nucleophile**	**Nitrosation + Cyanamide/Urea/Urea+H_2O**	***Het-NH-X** (*Note that exocyclic amine is lost and replaced with cyanamide derivative, so the adduct has the same chemical formula as Het-X)	**10** (None of which are likely from nitrosation)	**adducts with same mass likely from HCN nuc. substitution**	**10** (8 of which believed to be from nitrosation)	**# 1, 3, 15, 16, 26, 27, 28, 38** (see Carbonyl (HCN) for list of hets with same Δ m/z but believed to be formed via a reaction with HCN)
Nucleophile	Formaldehyde + Cyanamide / Formaldehyde + Urea / Formaldehyde + Urea + H_2O	Het-CH_2-NH-X	6	# 21, 24, 26, 32, 35, 43	7	# 1, 8, 9, 20, 22, 35, 38
Nucleophile	Methylamine / Formaldehyde + NH_3 or Degradation of adduct from Formaldehyde + Urea + H_2O	Het-NH-CH_3 / Het-CH_2-NH_2	4	# 26, 35, 43, 47	3	# 1, 24, 35
Nucleophile	Guanidine (nuc. substitution) / Urea (substitution)	Het-NH-CHN-NH_2/ Het-NH-CO-NH_2	2	# 24, 26	4	# 1, 8, 20, 26
Nucleophile	Formamidine	Het-CH(NH_2)-NH_2	0	ND	4	# 21, 24, 35, 39
Nucleophile	Glycine nitrile (electrophile)	Het-C(CH_2-NH_2)=NH	0	ND	4	# 1, 18, 20, 27
Nucleophile	Formamide (electrophile)	Het-(CHOH)-NH_2	1	# 35	2	# 20, 35
Nucleophile	Ammonia	Het-NH_2	2	# 26, 46	1	# 26
Nucleophile/Carbonyl	Cyanoacetonitrile (electrophile) / Imidazole (nuc. substitution)	Het-C(CH_2-CN)=NH / Het-imidazole	7	# 1, 2, 3, 24, 26, 32, 43	3	# 20, 23, 24
Nucleophile/Carbonyl	Cyanogen	Het-C(CN)=NH	1	# 24	6	# 18, 21, 27, 36, 38, 39
Nucleophile/Carbonyl	HCN trimer	Het-C(CH(NH_2)-CN)=NH	1	# 26	3	# 21, 26, 27
Nucleophile/Carbonyl	Amino glycine	Het-NH-CH(NH_2)-COOH	1	# 22	2	#23, 38

Table S7. Summary of yields of carbonylated nucleobases (adenine (A), guanine (G), cytosine (C), and uracil (U)) generated under abiotic conditions as reported by previous studies.

Nucleobase Adduct Het = N-heterocycle	Reaction	Conditions	A	G	C (and derivatives)	U (and derivatives)	Reference
Het-CHO	0.33 M N-heterocycle + 0.33 NaCOO + 30 mL formic acid	Reflux, 2 h	N/A	N/A	5,6-diamon-iso-C-N5-adduct (85%)<br>5,6-diamino-DAP-N5-adduct (77%)	N/A	67
	neat formamide (polymerization)	TiO_2 (2% w/w), 160 °C, 48h, under sunlight	A-N6,N9-bisadduct (0.04%)<br>Adenine (0.01 %)<br>Purine-N9-adduct (0.33%)<br>Purine (0.56%)	N/A	Cytosine (0.032%)	Thymine (0.007%)	22
	neat formamide (polymerization)	Montmorillonites (2% w/w), 160 °C, 48h	Adenine (2.7-22%)<br>Purine (3-34%)<br>Purine-N9-adduct (14-21%)	N/A	Cytosine (11-16%)	Uracil (0.5-2.0%)	23
Het-CH_2-COOH	1 mM nucleobase + 30 or 70 mM NaCN + 30 or 70 mM H_2CO*	pH 9.6, 25 °C, 1 yr	0.04 to 0.15% N9-adducts for purines & N1-adducts for pyrimidines				10
	Nucleobase + 0.1 M CH_2O<br>Het-CH_2OH + 0.01 M HCN	pH 7, 25 °C, $t_{1/2}$ 36 d<br>pH 7, 100 °C, 25 h	N/A	N/A	N/A	5-HMU (>99%)<br>U-C5-CH_2CN (99%)	1
	2.5 M glycine + 7.5 M NH_4OH + 10 M HCN*	pH 9.8, 80 °C, 18 h	A-N9-adduct (0.0062%)<br>A-N6-adduct (0.0013%)<br>Adenine (0.0051%)	G-N9-adduct (0.011%)<br>Guanine (0.0005%)	N/A	N/A	10
	1 mM cyanoacetaldehyde + 2 M hydantoic acid†	pH 7 (drifted to 9), 100 °C, 30 d	N/A	N/A	C-N1-adduct (18%)<br>C-N3-adduct (14%)	U-N1-adduct (1.8%)	10
	1 mM cyanoacetaldehyde + 2 M urea + 2 M hydantoic acid†	pH 7 (drifted to 9), 100 °C, 30 d	N/A	N/A	Cytosine: C-N1-adduct (8:1)	N/A	10
Het-CH_2-CH_2-CN	22 mM nucleobase + 1.1 M acrylonitrile†	deoxyguanosine: pH 7, 50 °C, 336 h<br>A: pH 8, 50 °C, 120 h<br>U/T: pH 8, 50 °C, 24 h‡	A-N9-adduct (18%)<br>A-N7-adduct (5%)	N9-deoxyguanosine-N7-adduct (31%)	N/A	U-N1-adduct (10%)<br>T-N1-adduct (11%)	9
Het-CH_2CH_2-$COCH_2CH_3$	22 mM nucleobase + 1.1 M ethylacrylate†	N9-deoxyguanosine: pH 7, 50 °C, 336 h<br>A: pH 8, 25 °C, 120 h<br>U/T: pH 8, 25 °C, 168 h	A-N9- adduct (12%)<br>A-N7- adduct (2%)	N9-deoxyguanosine-N7- adduct (34%)	N/A	U-N1- adduct (16%)<br>T-N1-adduct (20%)	9
CH_3<br>Het-CH-CH_2-$COCH_2CH_3$	22 mM nucleobase + 1.1 M ethylcrotonate†	pH 8, 25 °C, 168 h	N/A	N/A	N/A	U-N1-adduct (12%)<br>T-N1-adduct (7 %)	9

Table S7 Continued

Nucleobase Adduct Het = N-heterocycle	Reaction	Conditions	A	G	C (and derivatives)	U (and derivatives)	Reference
Het-CH=CH-CN	0.1 M nucleotide + 0.6 M cyanoacetylene + 0.1 M n-Bu_3-N + 3.5 or 3.9 mM $HgCl_2$	in 50% tert-butanol 0 ℃, 5 d	AMP-N1,N6-cyclic adduct (55%)	GMP-adduct formed, not characterized or quantified	CMP-N3,N4-cyclic adduct (45%)	UMP-adduct formed, not characterized or quantified	15
	0.53 M nucleobase + 1 mM cyanoacetylene	in BSTFA, 25 ℃, 1 h (after added drops H_2O)	N/A	N/A	N/A	T-N1-adduct (62%)	14
Het-CH_2-CH_2-CHO	1 mM nucleobase + 10 mM acrolein[†]	pH 9, 15 ℃, 40-60 min	A-N9-adduct (26%)<br>A-N1-adduct (14%)<br>A-N7-adduct (5%)	1 adduct (unable to determine yields)	C-N1-adduct (13%)<br>C-N3,N4-cyclic adduct (~1.3%)<br>C-N1,N3-cyclic adduct (~1.3%)	U-N1-adduct (70%)[‡]<br>U-N3-adduct (~7%)<br>T-N1-adduct (99% T consumed)[§]<br>Orotic acid-N1-adduct (12%)<br>5-HMU-N1-adduct (64%)<br>5-HMU-N3-adduct (~6.4%)	8
Het-CH=CH-CHO	0.32 M adenine + 0.32 M propiolaldehyde	in DMF + Na in ethanol -40 ℃, 1 h	A-N9-adduct (52.9%)	N/A	N/A	N/A	14
	0.26 M cytosine + 0.52 M propiolaldehyde	in BSTFA, 25 ℃, 4 h (recrystallized in H_2O)	N/A	N/A	C-N1-adduct (36.4%)	N/A	14
	0.53 M nucleobase + 1.06 M propiolaldehyde	in BSTFA, 25 ℃, 1 h (recrystallized in H_2O)	N/A	N/A	N/A	U-N1-adduct (82%)<br>T-N1-adduct (76.7%)<br>5-HMU-N1-adduct (59%)	14
Het-CH=CH-$COCH_3$	0.1 M adenosine + 0.12M 3-butyn-2-one	in ACN: CH_2Cl_2 (1:1) 20 ℃, 24 h	N9-Adenosine-1-adduct (20%)	N/A	N/A	N/A	68
	0.53 M nucleobase + 1.06 M 3-butyn-2-one	in BSTFA, 25 ℃, 1 h (recrystallized in H_2O)	N/A	N/A	N/A	T-N1 adduct (72%)	14

N/A = data not available

[*]Hydolyzed in 6 M HCl at 80 ℃ or 100 ℃ for 20-25 h to convert CN and $CONH_2$ groups to COOH

[†] Buffered with phosphate or carbonate

[‡] Longer reaction times resulted in two additions (i.e. bis adducts).

[§] Thymine is almost completely consumed, yet the N1-adduct decreases over time. This suggests that the reaction is pulled to completion due to the N1-adduct forming another product

4.0 SI Figures

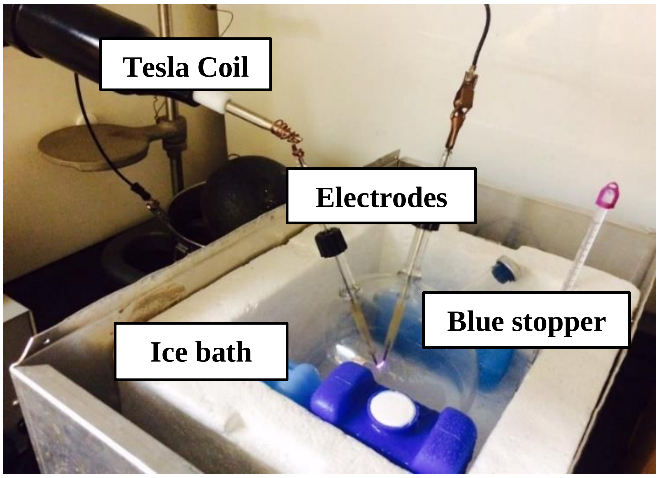

Figure S1. Spark discharge apparatus with tesla coil hooked to two tungsten electrodes. A blue stopper was used to prevent gas exchange with the atmosphere. Gasses were introduced into the flask using syringes in an anaerobic chamber. Ice packs were used to maintain the water bath at ~5 ℃ for the duration of the experiment.

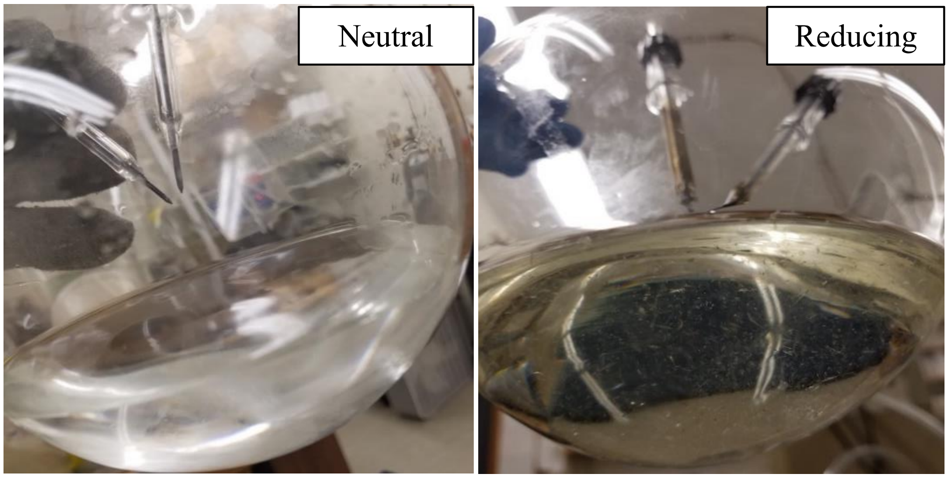

Figure S2: Photographs of Miller-Urey mixtures produced after sparking (40 kV) a neutral (N_2, CO_2) or reducing (N_2, CO_2, CH_4, H_2) gas mixture for 72 hours.

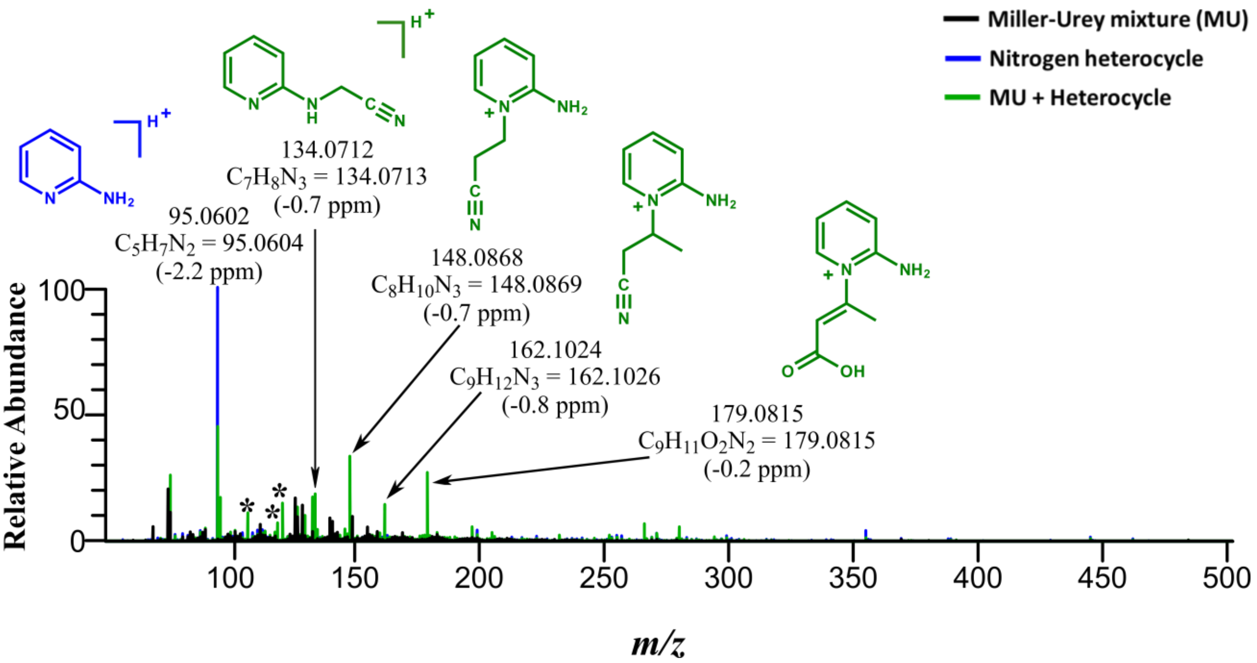

Figure S3. An example of the various carbonylated adducts that form in a single reaction mixture containing Miller-Urey organics and a N-heterocycle. Shown is the spectra from incubation (80° C, 72 h) of 1 mM 2-aminopyridine (*m/z* 95.0602) with a Miller-Urey spark mixture generated under a reducing atmosphere (1 bar of 0.4 N_2, 0.1 CO_2, 0.25 CH_4, and 0.25 H_2); a variety of nitrile side chains were identified. Isolated reactions with 2-aminopyridine and various Michael acceptors and glycolonitrile generate the above adducts. Notably, except for the glycolonitrile adduct (*m/z* 134.0712), the other products highlighted are all the result of Michael additions and as such have similar structures (i.e. 3-carbons long with a terminal carbonyl group). Over time the products are expected to hydrolyze, with the nitriles forming amides and carboxylic acids (the corresponding hydrolysis products were identified, see Table S2-3 for additional details). * Indicates a fragment from the products repeatedly identified in mass fragmentation of 2-aminopyridine adducts. Note that the structures drawn are used to illustrate the side chains and the location that they formed on the heterocycle has not been confirmed; other structural isomers may exist.

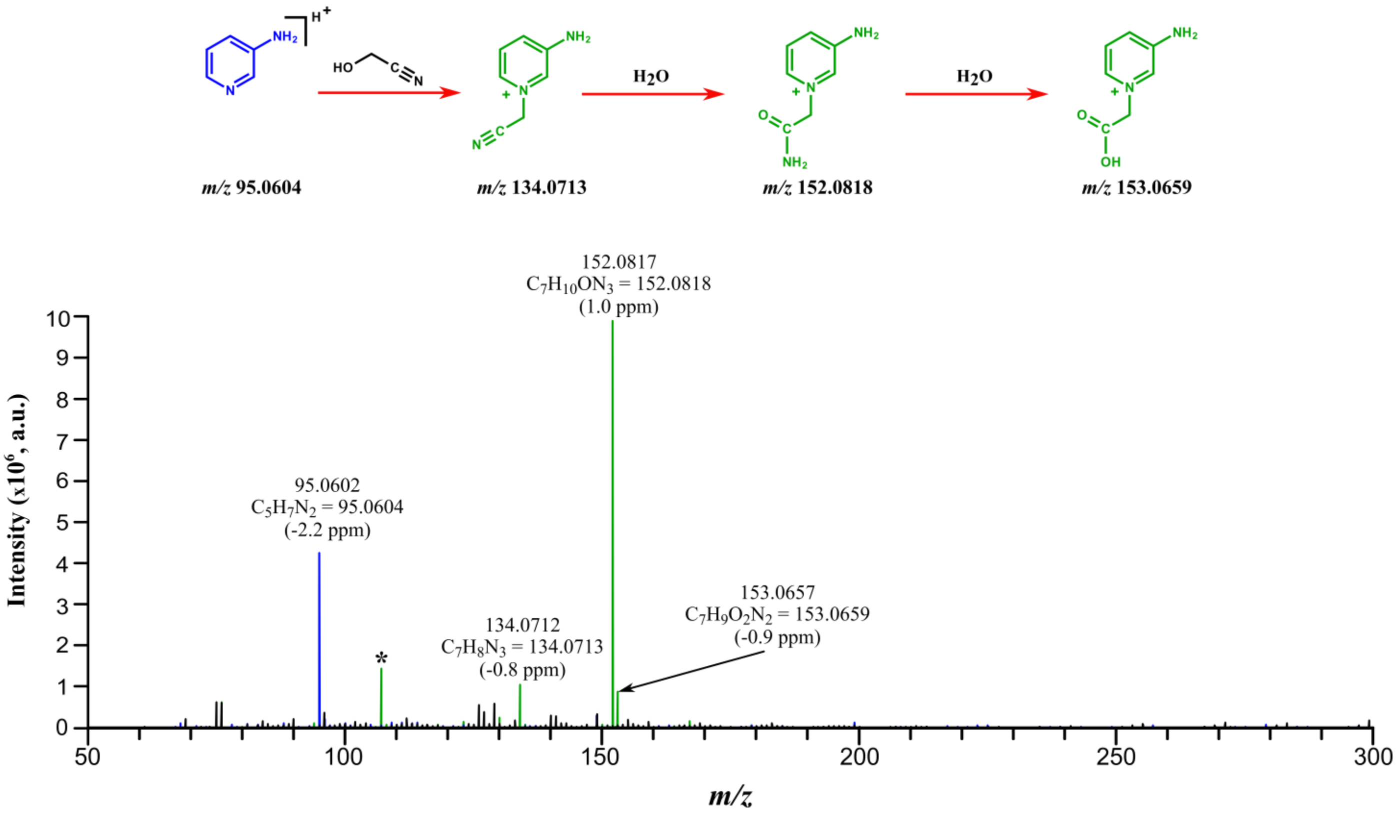

Figure S4: Example spectra that demonstrate the formation of the hydrolysis series of carbonylated adducts. Shown is the spectra from incubation (80 °C, 72 h) of 1 mM 3-aminopyridine (*m/z* 95.0602, protonated) with a Miller-Urey spark mixture generated under a reducing atmosphere (1 bar of 0.4 N_2, 0.1 CO_2, 0.25 CH_4, and 0.25 H_2); three high intensity products (*m/z* 134.0713, *m/z* 152.0818, and *m/z* 153.0657) were identified. Shown is the DART mass spectrum of the reaction mixture (green) overlaid by the spectra of the heterocycle control (blue) and Miller-Urey mixture (black); note that the N-heterocycle ionizes much more efficiently than the spark organics. * Indicates a fragment from the products identified in the mass fragmentation of each of the products (*m/z* 107.0602 = $C_6H_7N_2$). We confirmed that all three products are the result of glycolonitrile reacting with the heterocycle in a Strecker-like synthesis (reaction depicted above the mass spectrum); glycolamide does not react with heterocycles (not shown). Notice that the three carbonylated heterocycles are the result of the heterocycle reacting with **a single organic** out of the complex mixture. Over time the nitrile and amide products hydrolyze, eventually forming a carbonylated heterocycle with a carboxylic acid side chain. Structures have not been confirmed, the side chain may have formed at either the ring N as shown or on the exocyclic amine.

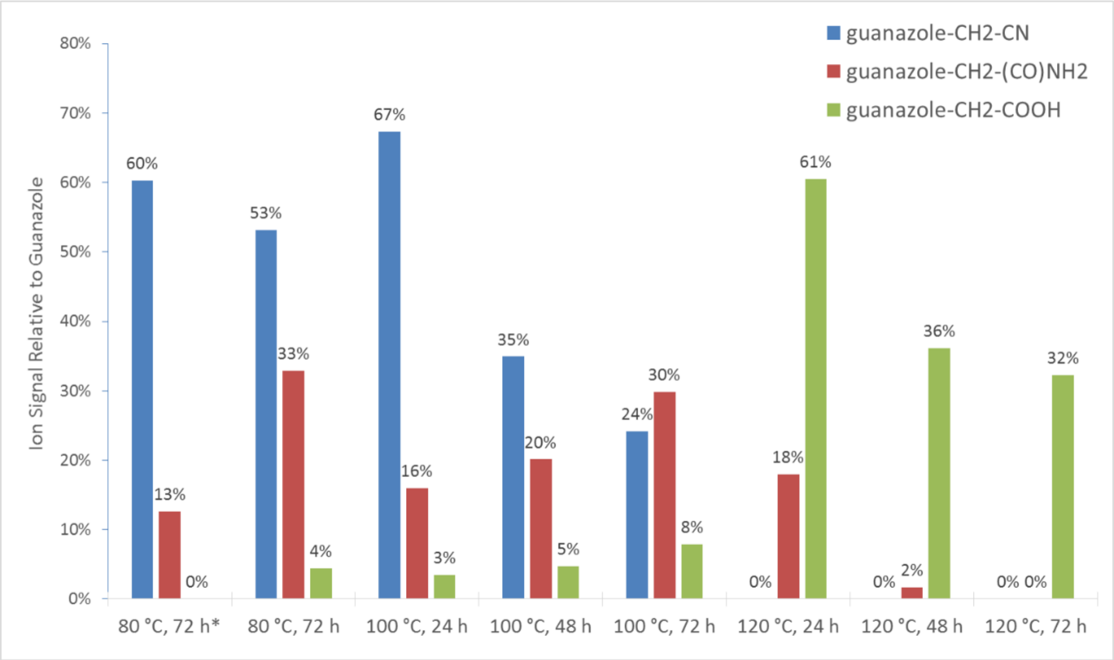

Figure S5. Glycolonitrile + guanazole reactions under various conditions. *indicates that heating occurred in an open system until solvent was completely evaporated, which took about 3 days.

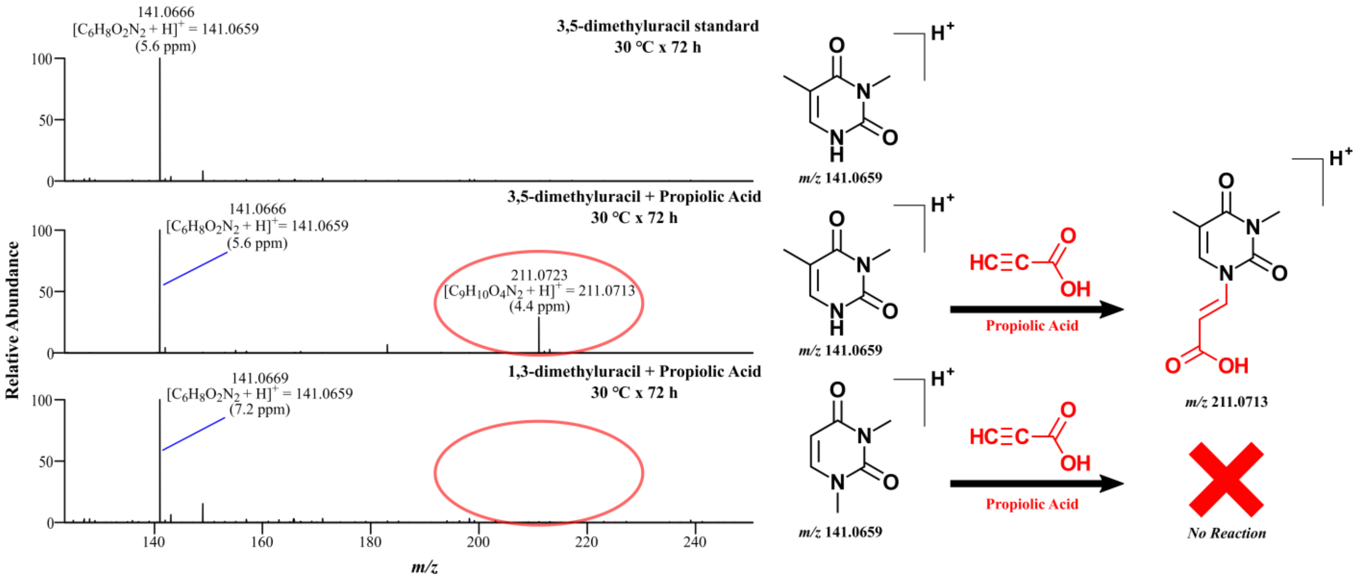

Figure S6. DART mass spectra of reactions between 1,3-dimethyluracil and 3,5-dimethyluracil with propiolic acid at 30 °C. There is only a single nucleophilic center available for each reaction: the N1 position for 3,5-dimethyluracil and the C5 for 1,3-dimethyluracil. Note that 3,5-dimethyluracil reacts with propiolic acid (middle panel) to produce the expected carboxylic acid adduct (*m/z* 211.0713, protonated), but 1,3-dimethyluracil does not (bottom panel); the top panel demonstrates that the carboxylic acid adduct is not from contamination of the 3,5-dimethyluracil solution. These results suggest that propiolic acid preferentially adds to the N1 position of uracil. The reaction scheme and product structure are shown to the right of the corresponding mass spectrum. The same results were observed when propiolic acid was added to a solution containing 3,5-dimethyluracil or 1,3-dimethyluracil and immediately frozen (see Table S6 for details).

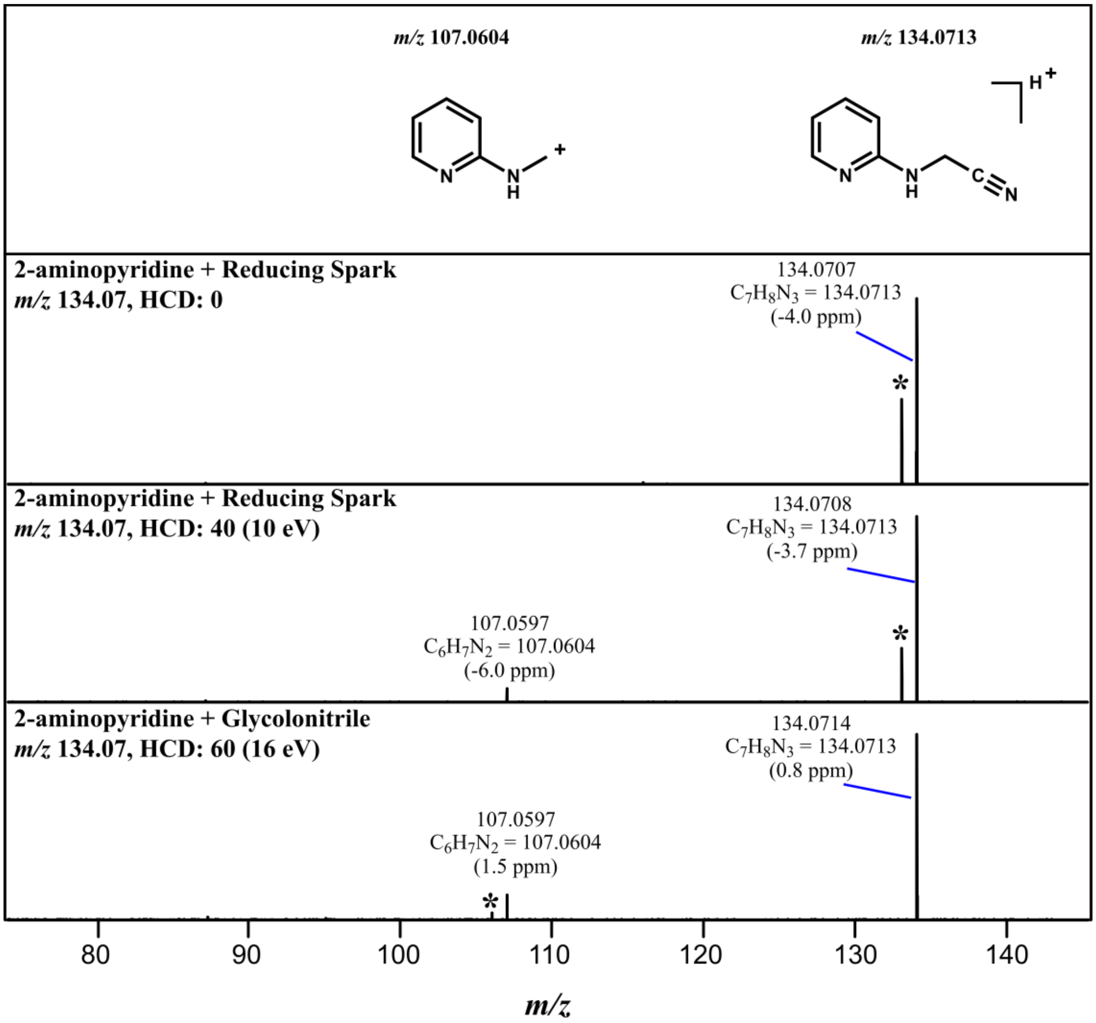

Figure S7: Product ion spectra of 2-aminopyridine adduct (*m/z* 134.07) produced via incubation of 2-aminopyridine with a spark mixture generated under a reducing atmosphere. Fragmentation of the precursor ion produces fragments consistent with a two-carbon nitrile side chain (i.e., HCN loss (*m/z* 107.0605)). The * indicates interferences in the fragmentation spectra (i.e., *m/z* 133.0754 and 106.0738) that have an assigned formula of $C_8H_9N_2$ and $C_3H_{10}O_2N_2$, respectively; as the isolated product ion only has 7 carbons, neither of these peaks can be fragments of the isolated adduct ($C_7H_8N_3$).

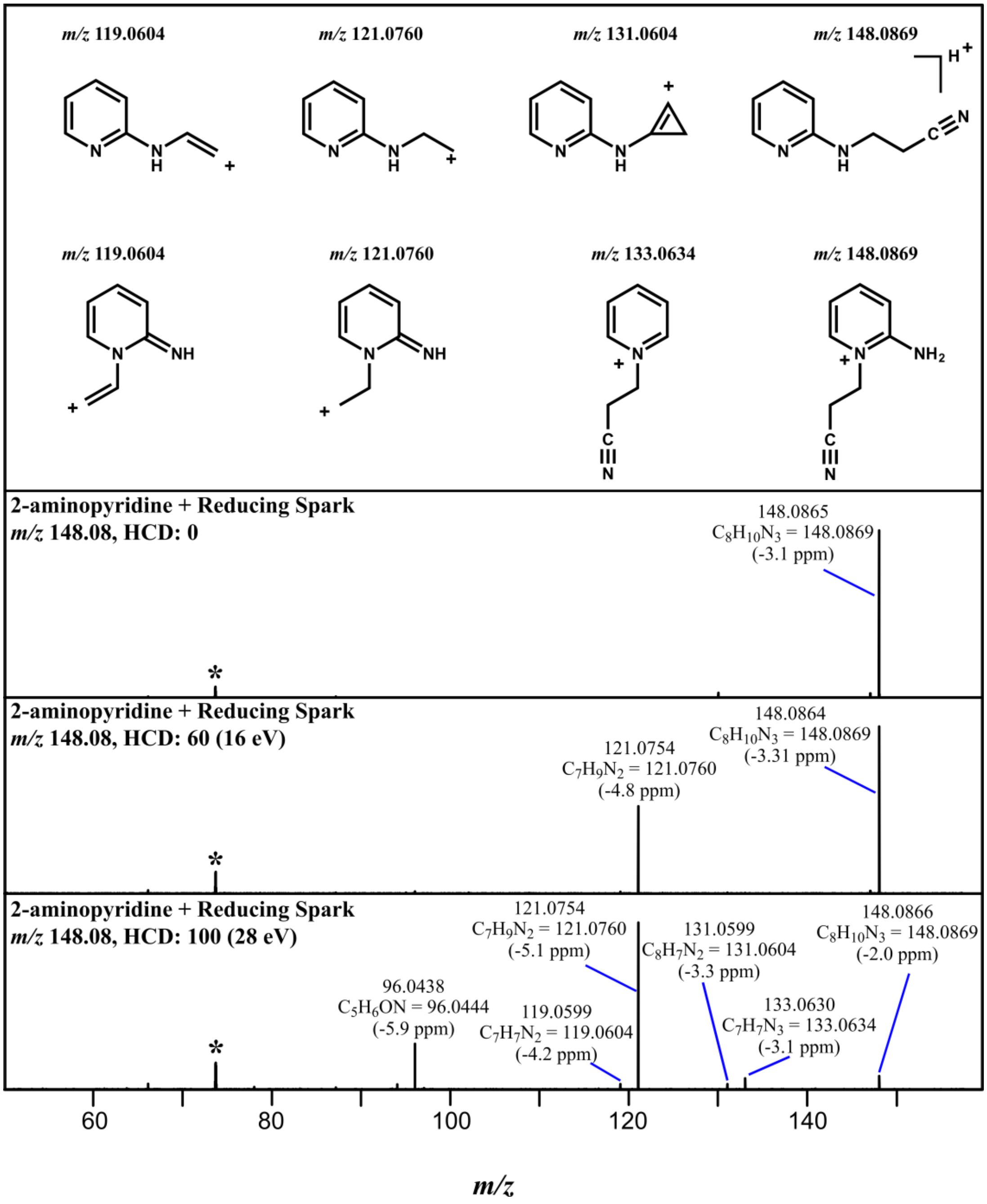

Figure S8: Product ion spectra of 2-aminopyridine adduct (*m/z* 148.08) produced via incubation of 2-aminopyridine with a spark mixture generated under a reducing atmosphere. Fragmentation of the precursor ion produces fragments consistent with a three-carbon nitrile side chain (i.e., HCN loss (*m/z* 161.0709)) attached to the ring nitrogen of 2-aminopyridine: the fragment, *m/z* 96.0444 is generated from 2-aminopyridine. In addition, the *m/z* 133.0599 fragment is consistent with NH_2 loss, suggesting that the N1 adduct formed (although this does not preclude the possibility of the adduct forming on the exocyclic amine group as well). In fact, the fragment *m/z* 131.0599 is predicted to be from the adduct with the side chain at the exocyclic amine. The * indicates a common instrument artifact (centered at *m/z* 73.7).

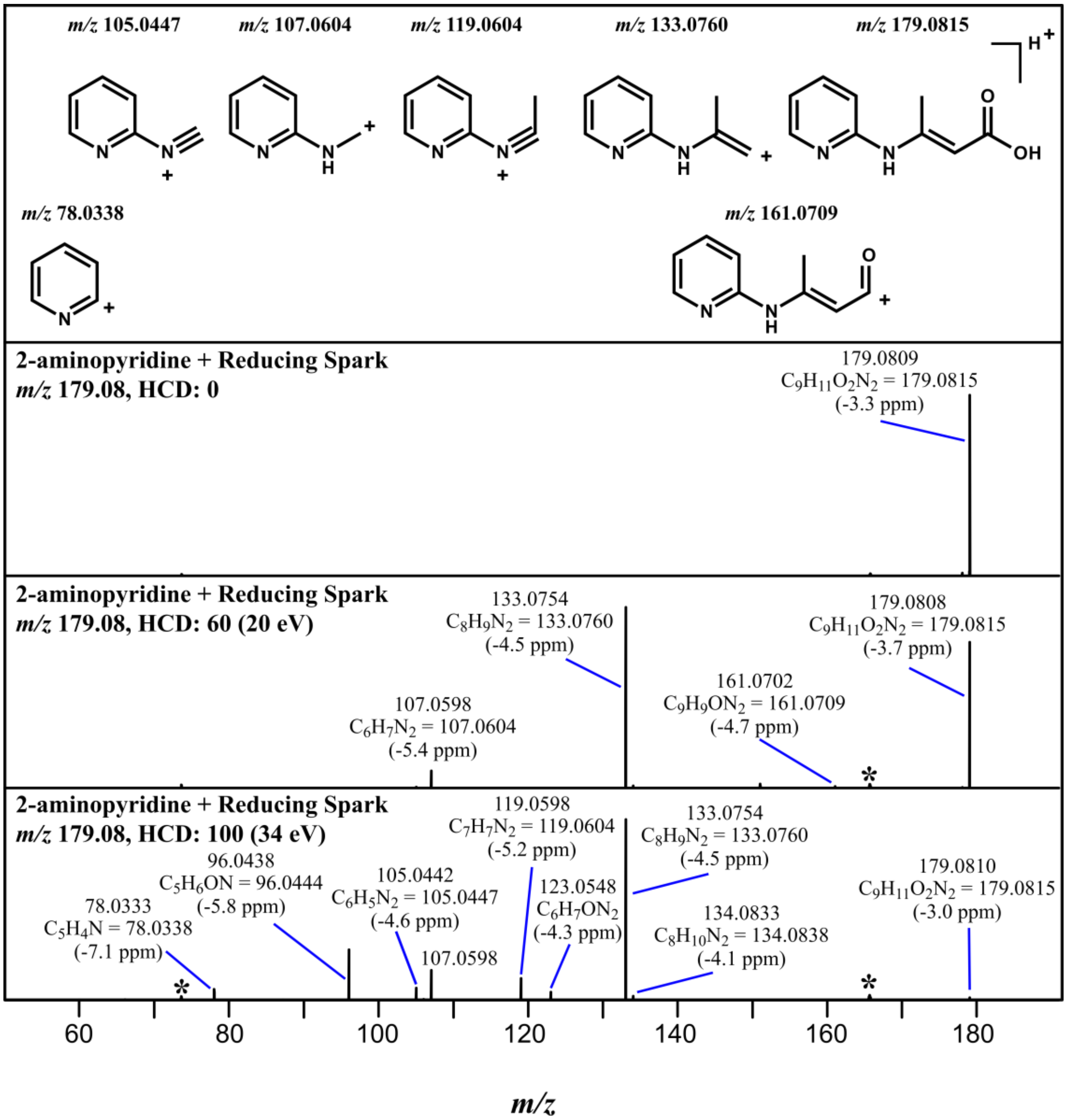

Figure S9: Product ion spectra of 2-aminopyridine adduct (*m/z* 179.08) produced via incubation of 2-aminopyridine with a spark mixture generated under a reducing atmosphere. Fragmentation of the precursor ion produces fragments consistent with a three-carbon carboxylic acid side chain (i.e., H_2O loss (*m/z* 161.0709) and HCOOH loss (*m/z* 133.0760)) attached to 2-aminopyridine. The fragments, *m/z* 96.0444 (hydroxypyridine) and *m/z* 78.0338, are both generated from the 2-aminopyridine ring (confirmed by MS/MS analysis of a 2-aminopyridine standard at HCD 100, data not shown). Note that the fragments drawn represent only one of two possible structures (the other being addition at the cyclic N1 position). The * indicates two common instrument artifacts (centered at *m/z* 165.9 and 73.7).

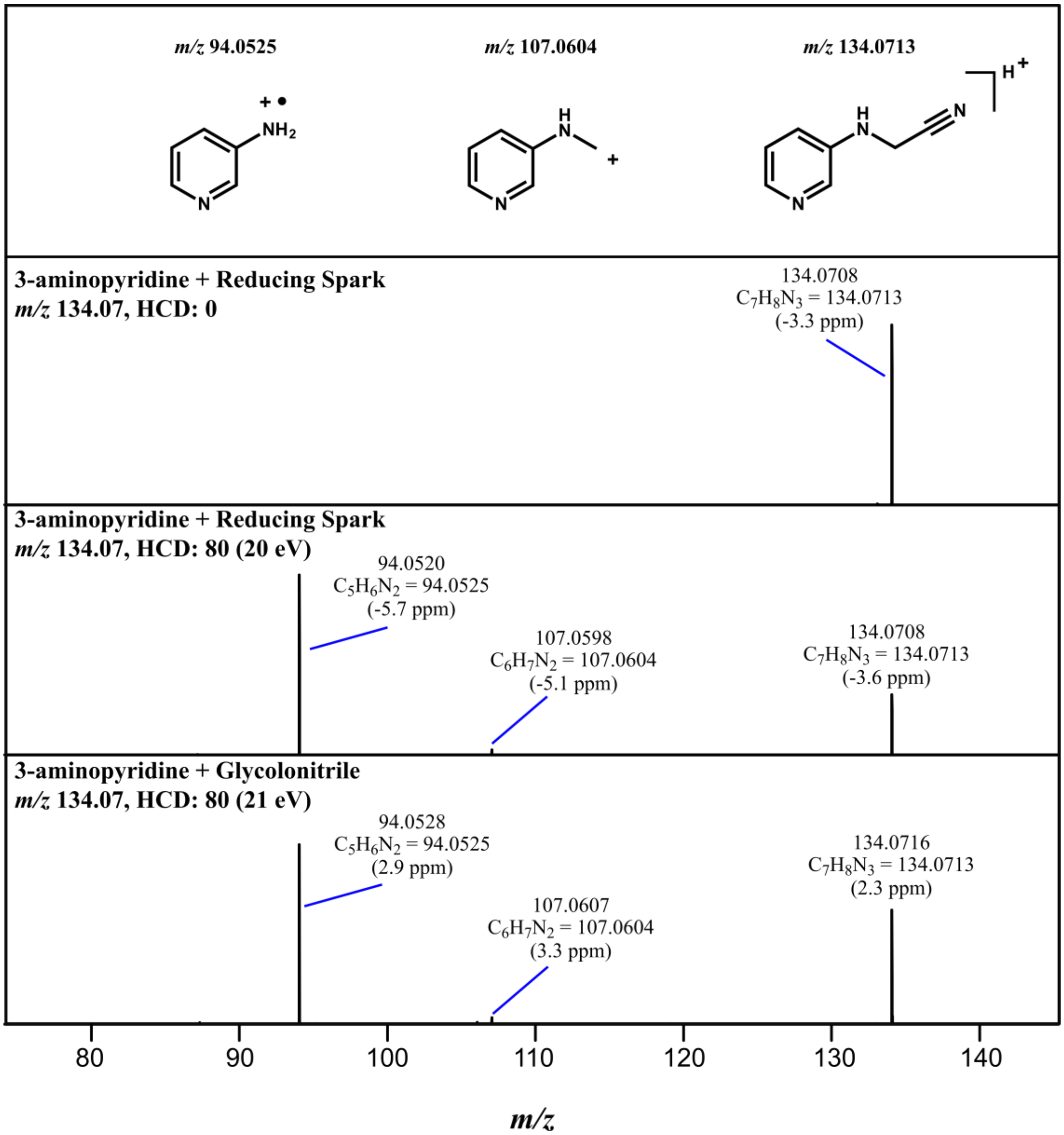

Figure S10: Product ion spectra of 3-aminopyridine adduct (*m/z* 134.07) produced via incubation of 3-aminopyridine with a spark mixture generated under a reducing atmosphere (second and third panel from the top) matches that generated from 3-aminopyridine and glycolonitrile (same *m/z* precursor ion, bottom panel). Fragmentation of the precursor ion produces fragments consistent with a three-carbon nitrile side chain (i.e., HCN loss) attached to 3-aminopyridine (*m/z* 94.0525). Note that the fragments drawn represent only one of two possible structures (the other being addition at the ring nitrogen).

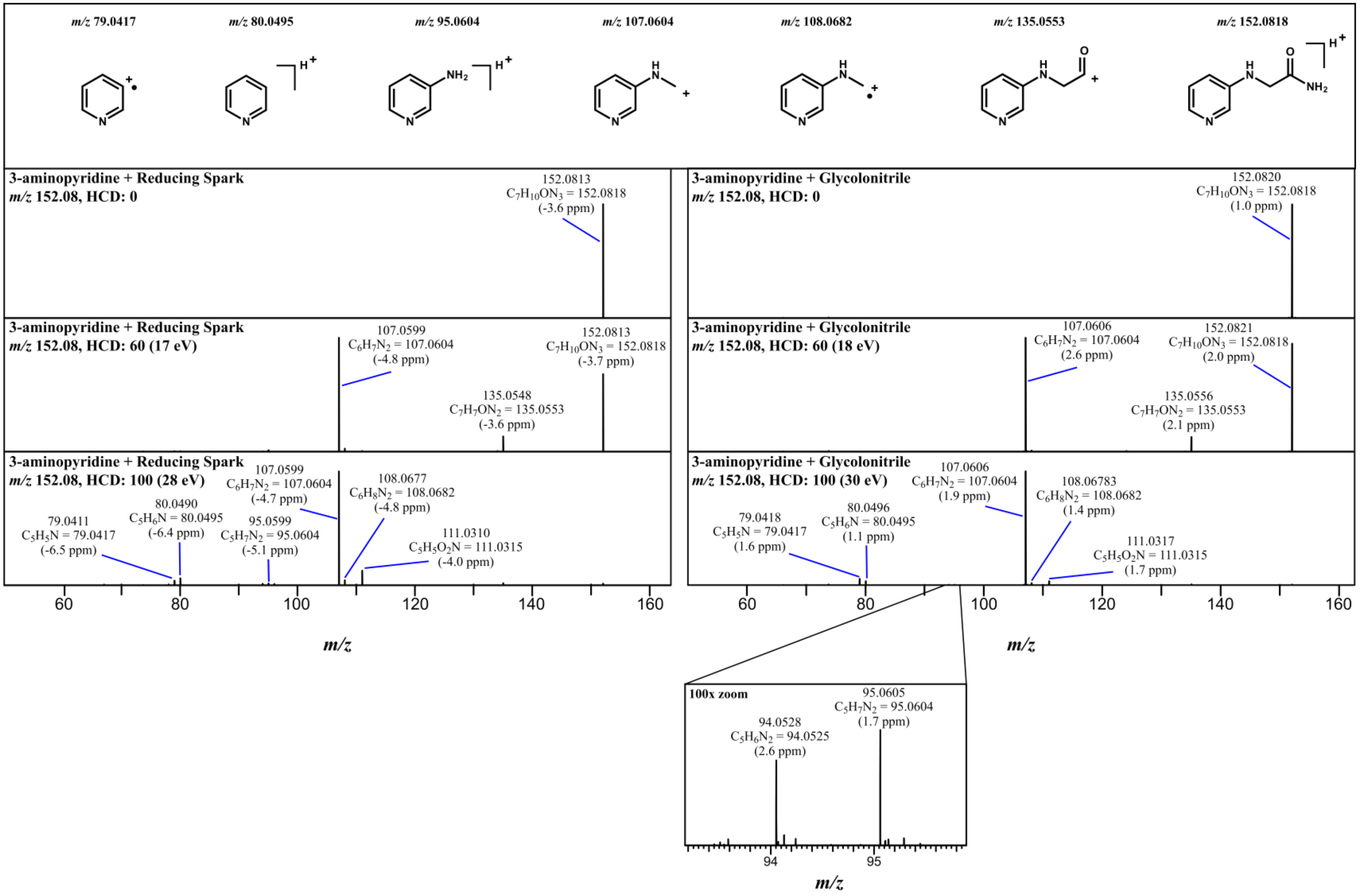

Figure S11: Product ion spectra of 3-aminopyridine adduct (*m/z* 152.08) produced via incubation of 3-aminopyridine with a spark mixture generated under a reducing atmosphere (left) matches that generated from 3-aminopyridine incubated with glycolonitrile (right). Fragmentation of the precursor ion produces fragments consistent with a three-carbon amide side chain (i.e., NH_3 and $HCONH_2$ loss) attached to 3-aminopyridine (*m/z* 95.0604). Note that the fragments drawn represent only one of two possible structures (the other being addition at the ring nitrogen).

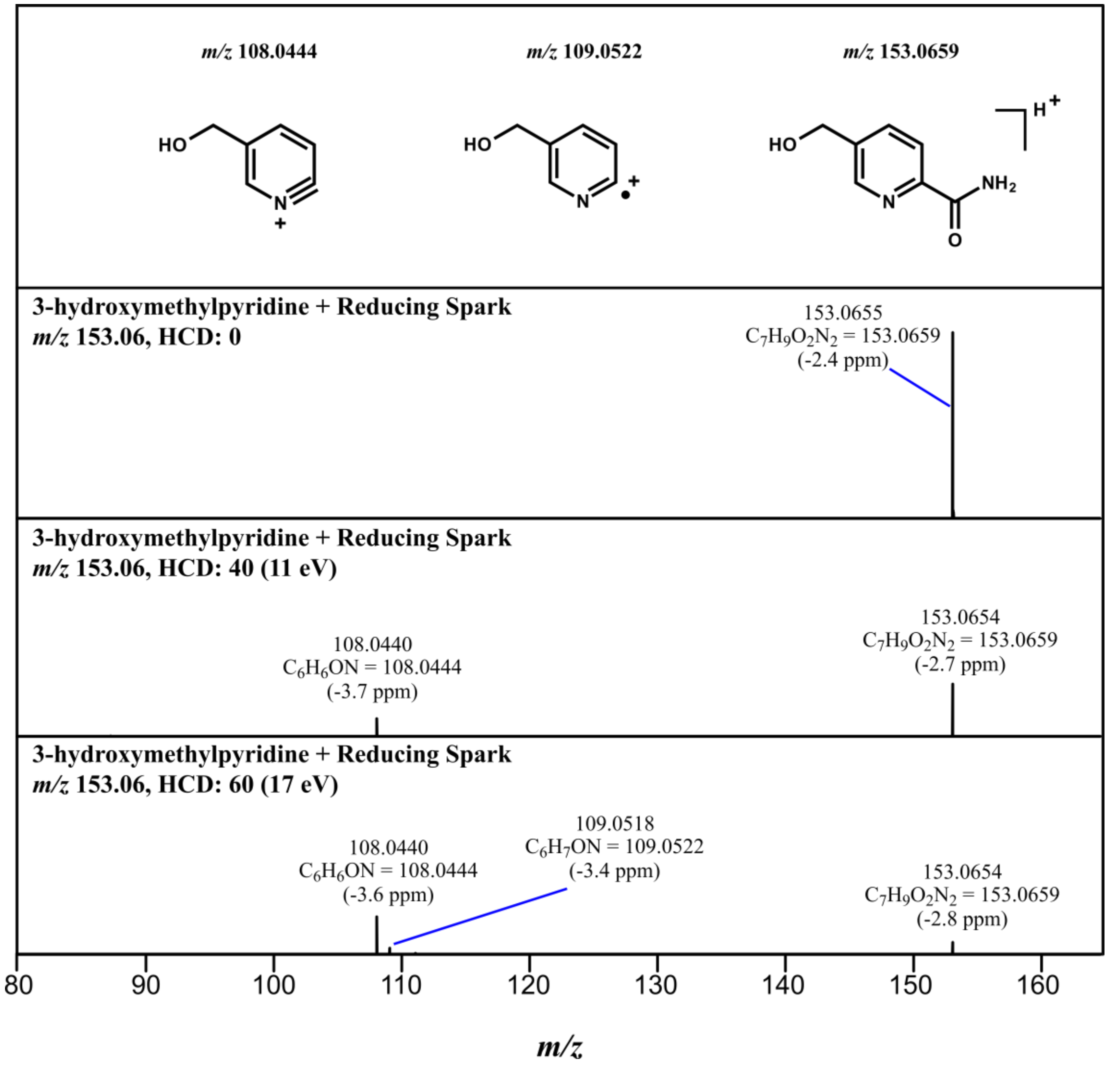

Figure S12: Product ion spectra of 3-hydroxymethylpyridine adduct (*m/z* 153.06) produced via incubation of 3-hydroxymethylpyridine with a spark mixture generated under a reducing atmosphere. Fragmentation of the precursor ion produces fragments consistent with a CH_2ON-containing side chain attached to 3-hydroxymethylpyridine. Note that the fragments drawn represent only one possible structure (the other being addition at one of the other ring carbons).

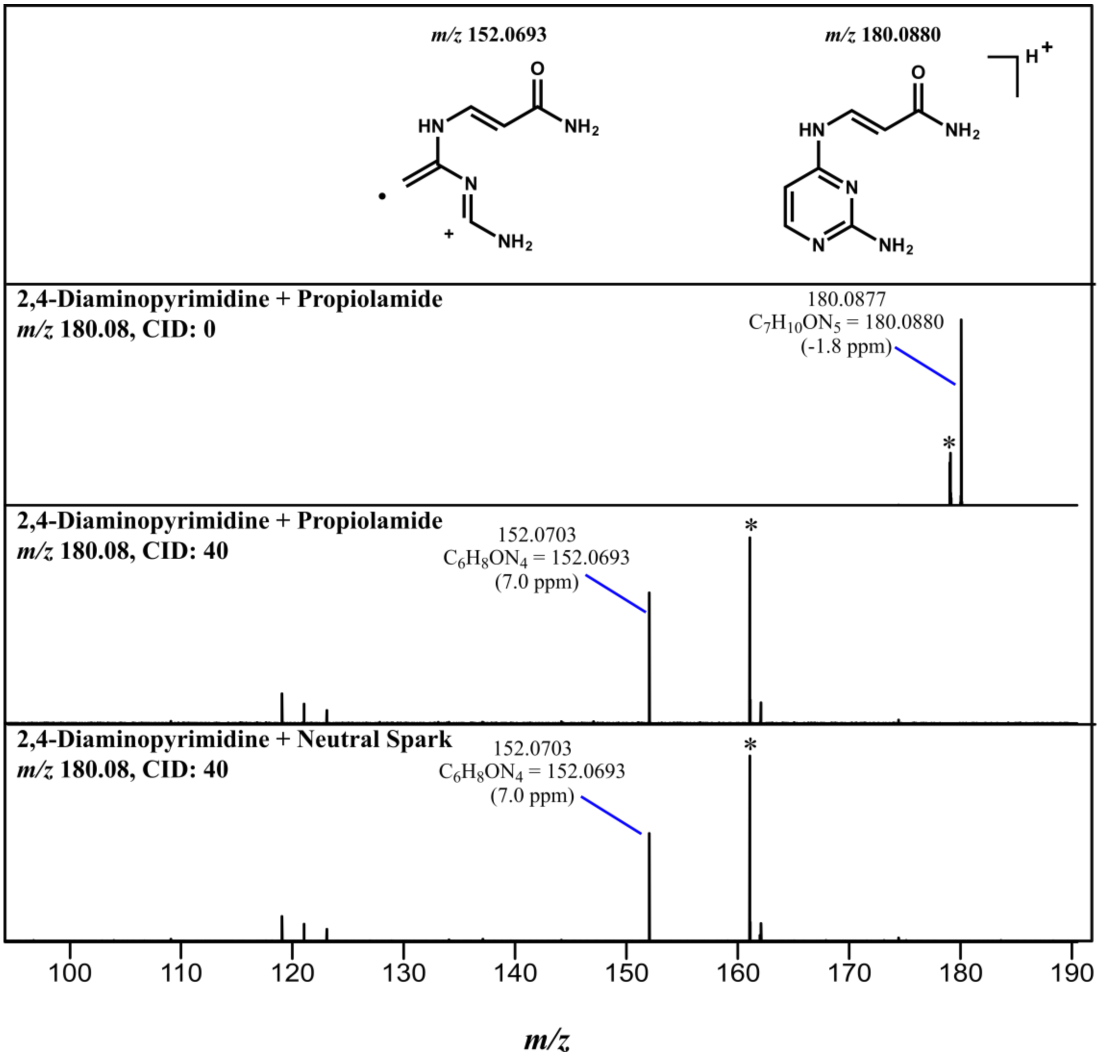

Figure S13: Product ion spectra of 2,4-diaminopyrimidine adduct (*m/z* 180.08) produced via incubation of 2,4-diaminopyrimidine with a spark mixture generated under a neutral atmosphere matches (bottom panel) that generated from 2,4-diaminopyrimidine incubated with propiolamide (second and third panels from the top). The * indicates interferences in the fragmentation spectra (i.e., *m/z* 179.1058 and 161.0958): having an assigned formula of $C_9H_{13}ON_3$ and $C_9H_{11}N_3$, respectively neither of these peaks can be fragments of the target product ion ($C_7H_{10}ON_5$).

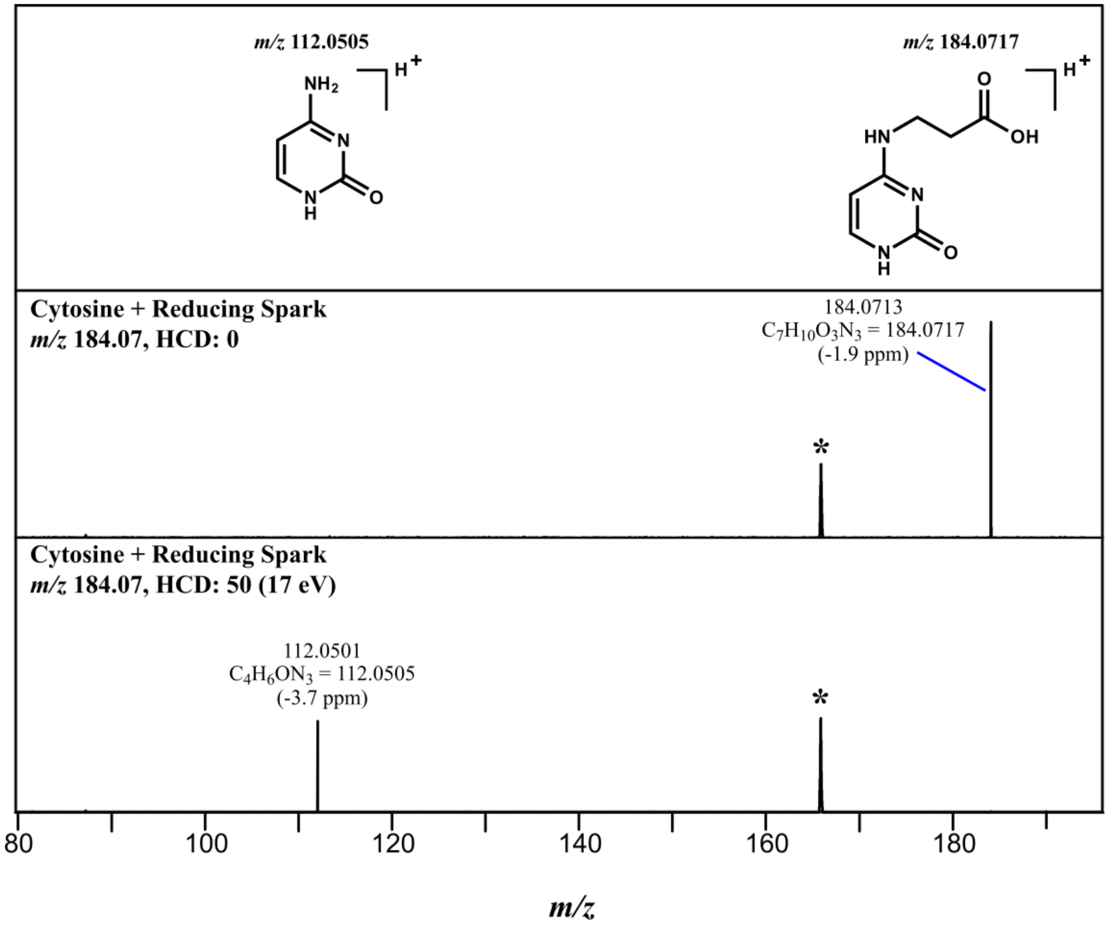

Figure S14: Product ion spectra of cytosine adduct (*m/z* 184.07) produced via incubation of cytosine with a spark mixture generated under a reducing atmosphere. Fragmentation of the precursor ion produces fragments consistent with a two-oxygen-containing side chain attached to cytosine (*m/z* 112.0501). Note that the precursor ion drawn represents only one possible structure as the reaction could have also occurred at the N1 ring nitrogen or C5 carbon. The * indicates a common instrument artifact (*m/z* 165.8). Note that this adduct was not included in Table S3 and was not listed as an adduct for cytosine (nor counted toward the total number of heterocycles that formed carbonyls in Table S6) as it was found with an intensity less than 1 magnitude of that in the spark-discharge control (when cytosine was added the signal intensity increased ~9 times the original amount). However, we have shown that cytosine reacts readily with acrylic acid (Table S5). Cytosine can be generated in spark discharge mixtures as its precursors are major products of spark discharge mixtures; in fact, we identified an ion with a mass corresponding to cytosine in the reducing spark mixture. Therefore, this carbonylated adduct may have been efficiently formed *in-situ*—that is from cytosine and acrylic acid generated in a one-pot reaction mixture derived from spark discharges through a reducing atmosphere.

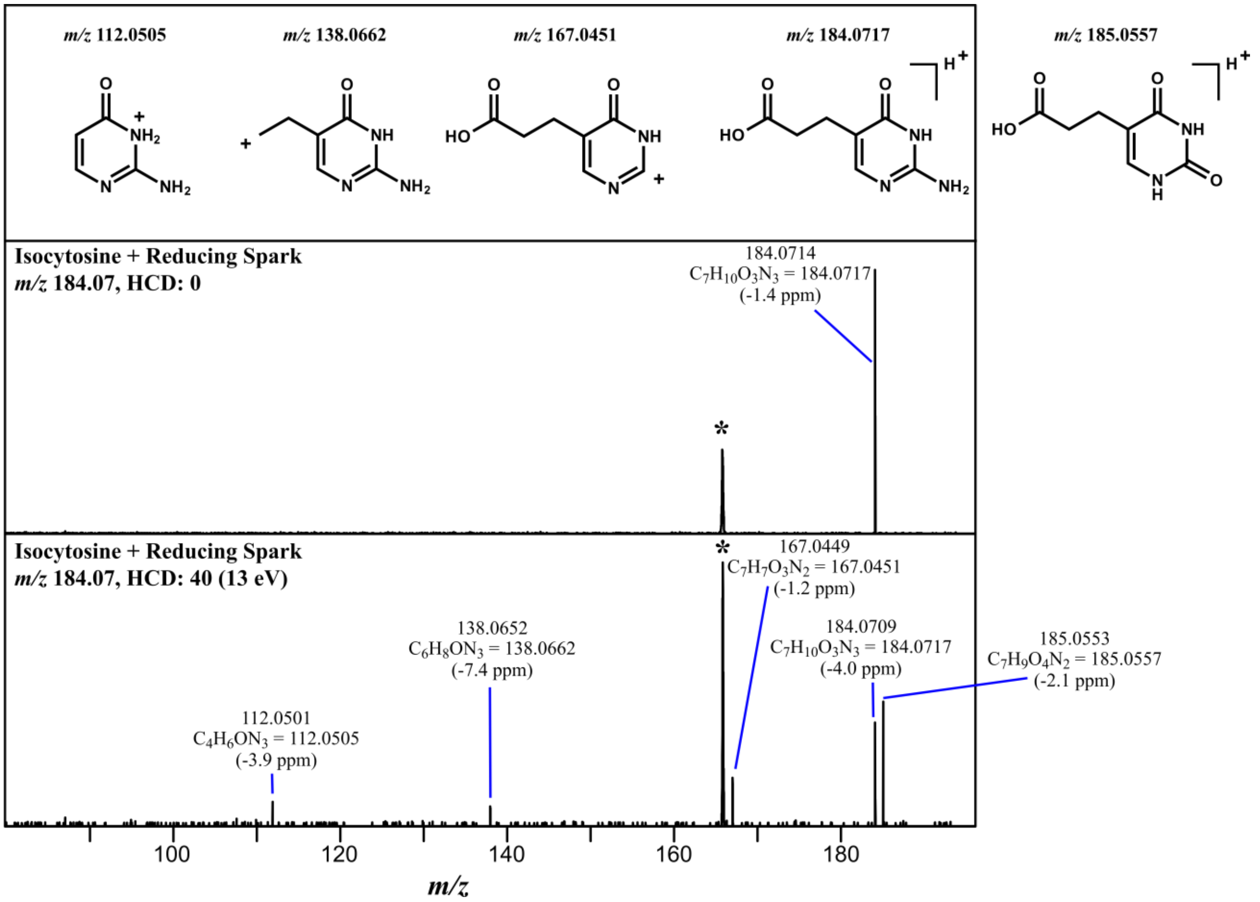

Figure S15: Product ion spectra of isocytosine adduct (*m/z* 184.07) produced from incubation of isocytosine with a spark mixture generated under a reducing atmosphere. Fragmentation of the precursor ion produces fragments consistent with a three-carbon carboxylic acid side chain (i.e., HCOOH loss) attached to isocytosine (*m/z* 112.0501). Note that the fragments drawn represent addition at the C5 atom; however, addition at the exocyclic amine as well as the N1 and N3 positions are also possible and cannot be ruled out. At 13 eV collision energy the precursor ion's exocyclic amine group is deaminated and hydrolyzed, forming uracil-propanoic acid (*m/z* 185.0557); this can only occur if the side chain was attached to the C5, N1, or N3 position and suggests that the reaction proceeded at least partially at a position other than the exocyclic amine in the spark mixture. The * indicates a common instrument artifact (*m/z* 165.9).

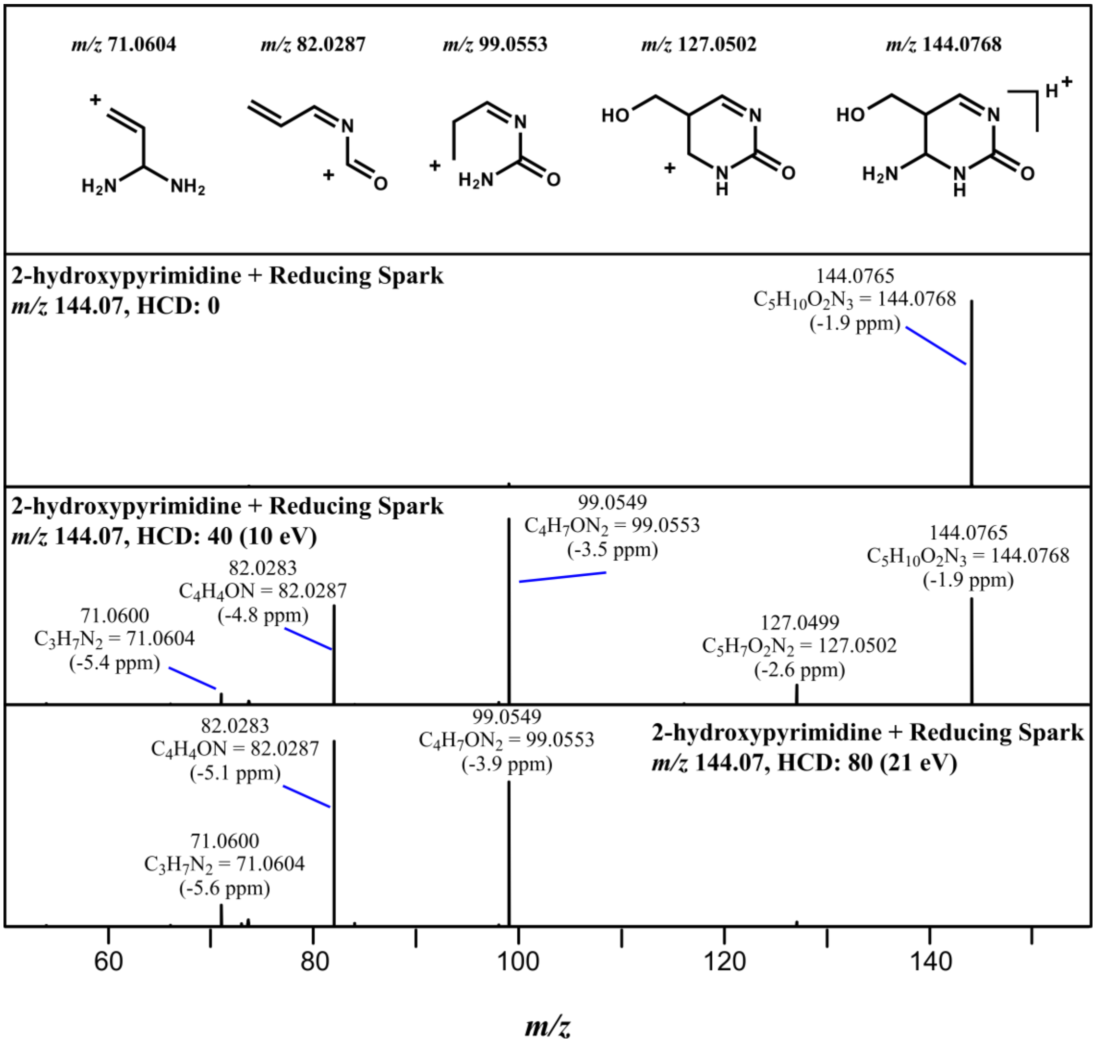

Figure S16: Product ion spectra of 2-hydroxypyrimidine adduct (*m/z* 144.07) produced via incubation of 2-hydroxypyrimidine with a spark mixture generated under a reducing atmosphere. Fragmentation of the precursor ion produces fragments consistent with loss of a terminal amine and hydroxymethyl group (i.e., NH_3 and CO loss). Note that other structural isomers may exist.

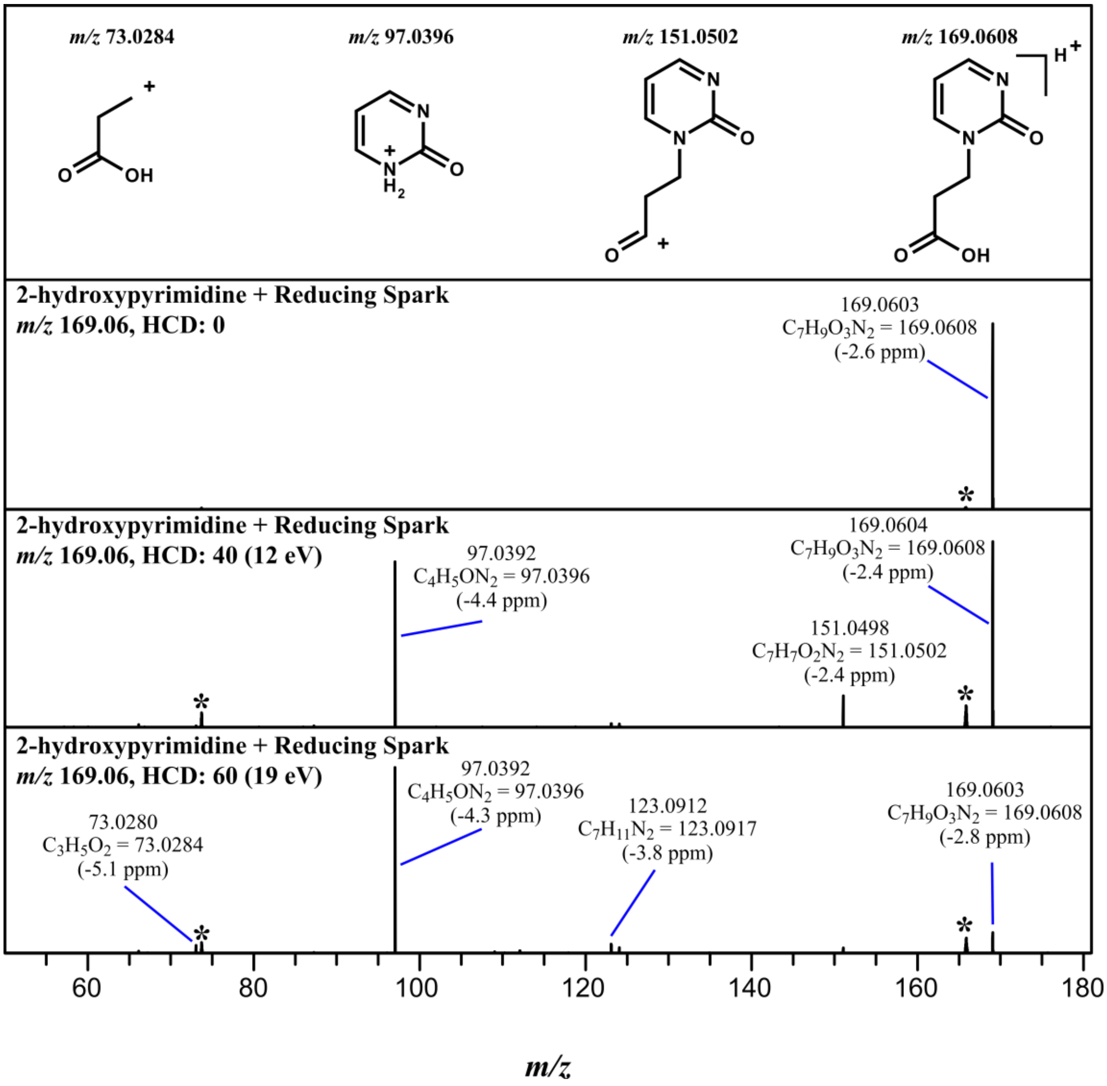

Figure S17: Product ion spectra of 2-hydroxypyrimidine adduct (*m/z* 169.06) produced via incubation of 2-hydroxypyrimidine with a spark mixture generated under a reducing atmosphere. Fragmentation of the precursor ion produces fragments consistent with a three-carbon carboxylic acid side chain (i.e., H_2O loss and fragment *m/z* 73.0284) attached to 2-hydroxypyrimidine (*m/z* 97.0396). Note that the fragments drawn represent only one of two possible structures (the other being addition at the C5 position). The * indicates two common instrument artifacts (centered at *m/z* 165.9 and 73.7).

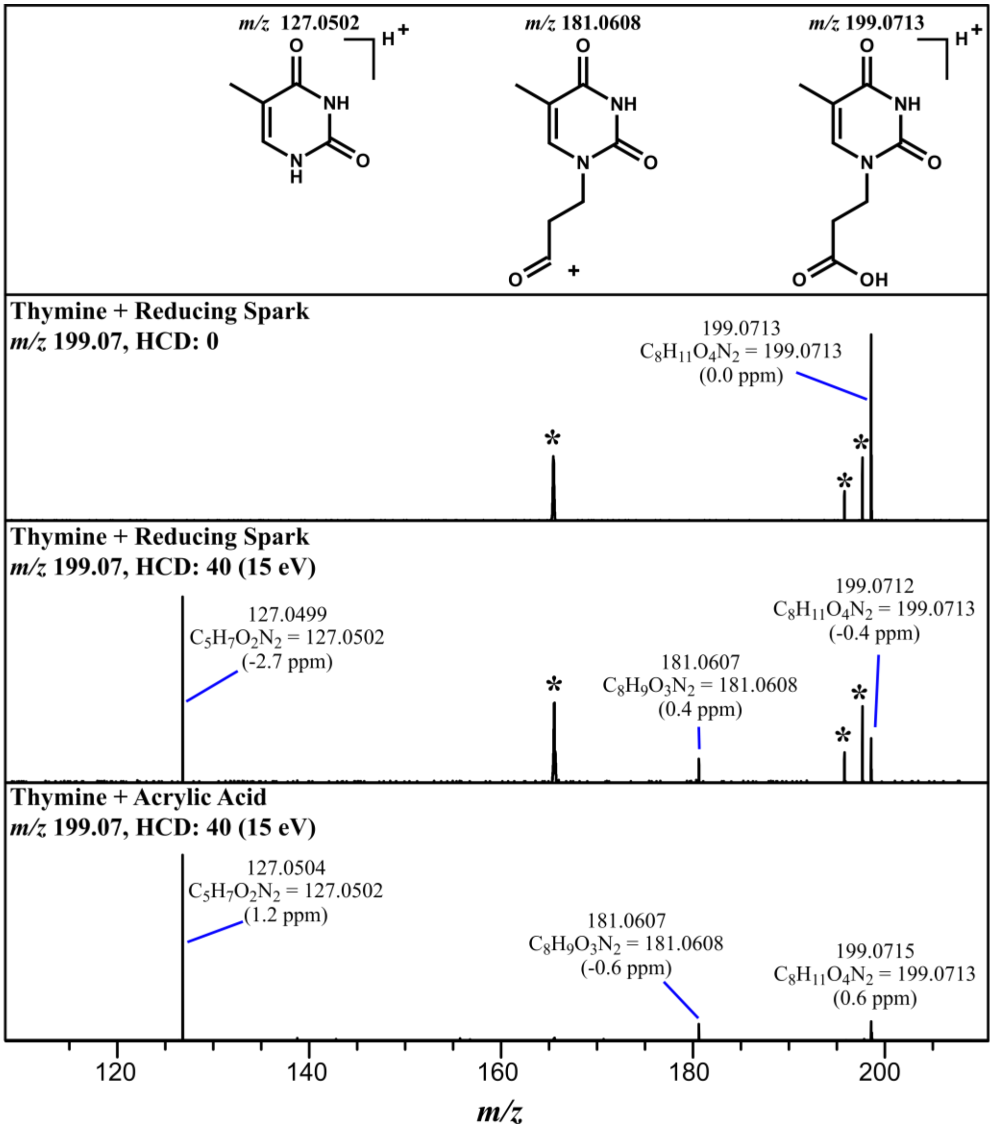

Figure S18: Product ion spectra of thymine adduct (*m/z* 199.07) produced via incubation of thymine with a spark mixture generated under a reducing atmosphere (second and third panels from the top) matches that from thymine incubated with acrylic acid (same *m/z* precursor ion, bottom panel). Fragmentation of the precursor ion produces fragments consistent with a side chain containing a terminal OH group (i.e., H_2O loss) attached to thymine (*m/z* 127.0502). * indicates a common instrument artifact (*m/z* 165.9) and interferences in the fragmentation spectra (*m/z* 196.2884 and *m/z* 198.1680) captured with the precursor ion (as seen by their presence in the HCD 0 spectra of the precursor ion isolated from the spark mixture).

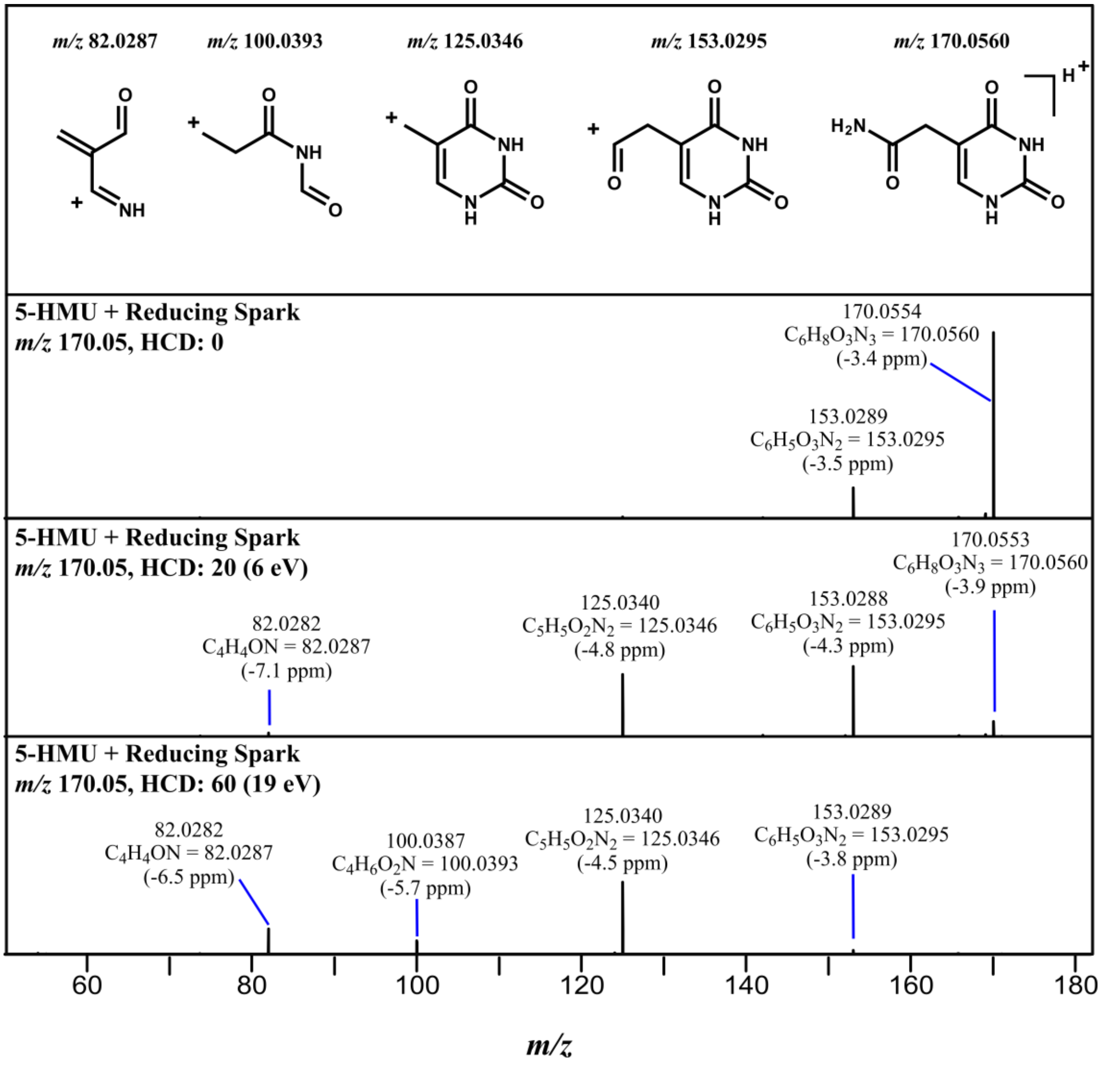

Figure S19: Product ion spectra of 5-hydroxymethyluracil (5-HMU) adduct (*m/z* 170.05) produced via incubation of 5-HMU with a spark mixture generated under a reducing atmosphere. Fragmentation of the precursor ion produces fragments consistent with a two-carbon amide side chain (i.e., NH_3 loss and $HCONH_2$ loss) attached to 5-HMU (note: 5-HMU fragments into *m/z* 125.0340 → 100.0387 → 82.0282).

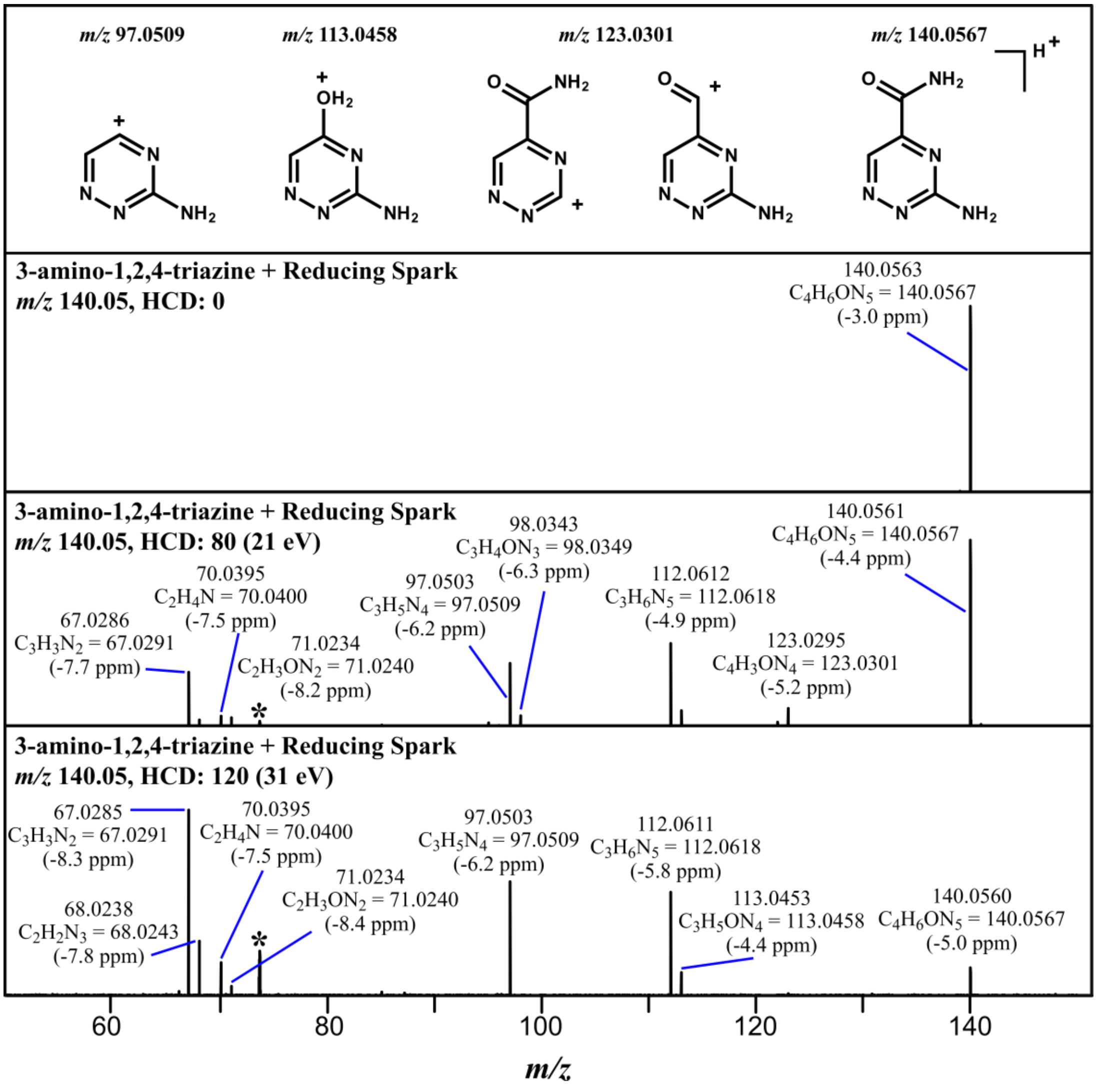

Figure S20: Product ion spectra of 3-amino-1,2,4-triazine adduct (*m/z* 140.05) produced via incubation of 3-amino-1,2,4-triazine with a spark mixture generated under a reducing atmosphere. Fragmentation of the precursor ion produces fragments consistent with an amide side chain (i.e., NH_2 and $CONH_2$ loss) attached to 3-amino-1,2,4-triazine (*m/z* 97.0509). Exact adduct structure has not been confirmed; however, it is likely that nucleophilic substitution of ^{-}CN would occur at the drawn C5 position as this atom is the most electron deficient in the ring and thus the most susceptible to nucleophilic attack. Incubation of 3-amino-1,2,4-triazine with HCN generates the same product (*m/z* 140.0567). The * indicates a common instrument artifact (*m/z* 73.6).

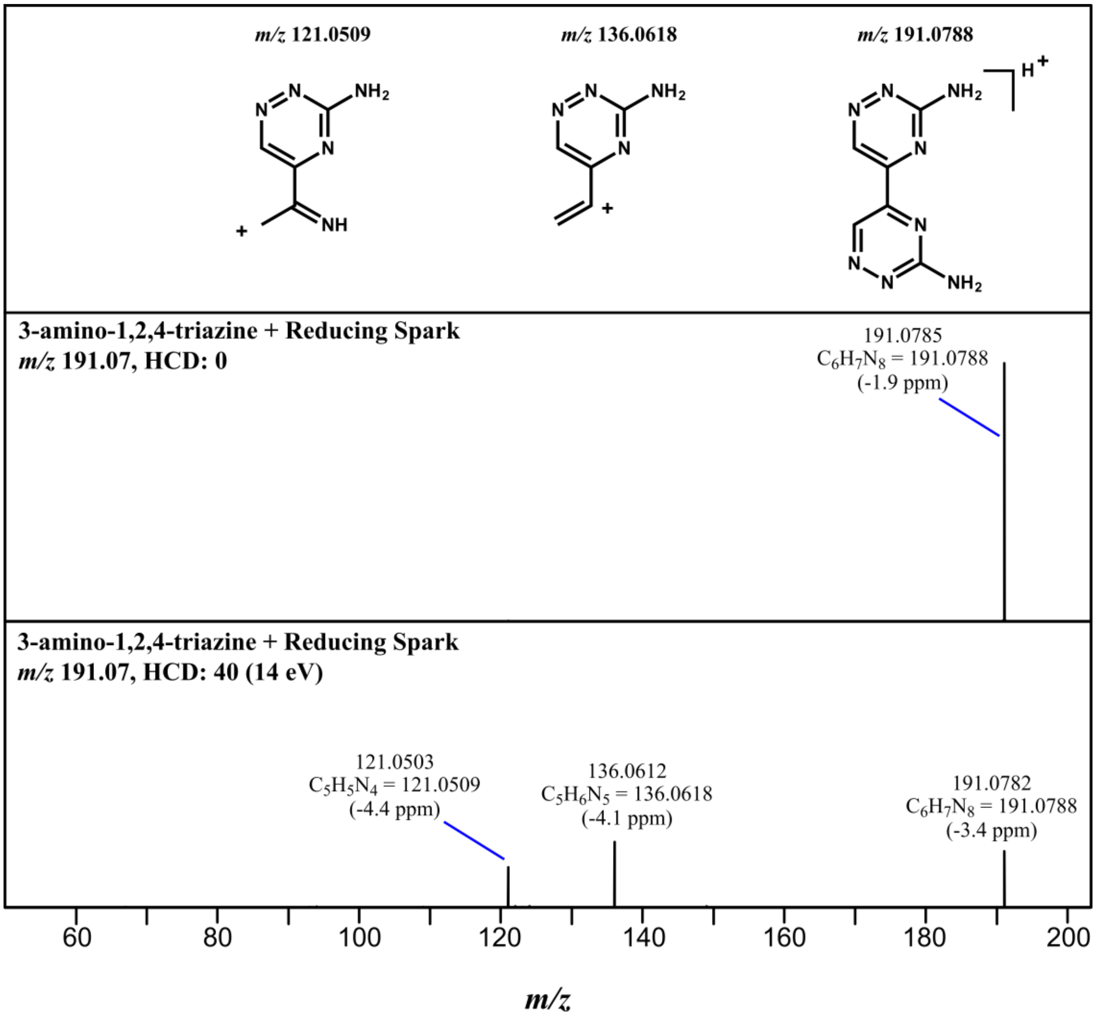

Figure S21: Product ion spectra of 3-amino-1,2,4-triazine adduct (*m/z* 191.07) produced via incubation of 3-amino-1,2,4-triazine with a spark mixture generated under a reducing atmosphere. Fragmentation of the precursor ion produces fragments consistent with a covalent dimer: both dimerization linked at the C5' positions (drawn) or by the exocyclic amine (not shown) are consistent with the fragmentation pattern. Note that the dimer is also formed in a solution 3-amino-1,2,4-triazine alone; however, when the spark mixture is added, the dimer ion count increases by 3 orders of magnitudes (from 10^4 to 10^7 counts, respectively). Similarly, adding NaCN to a solution of 3-amino-1,2,4-triazine increases the adduct *m/z* 191.0788 by 3 orders of magnitudes (10^7 counts, see Table S5). This result is also consistent with previous work that has shown that adding KCN to solutions of 5'-unsubstituted 1,2,4-triazines (including 3-amino-1,2,4-triazine) generates the 5-5' linked dimers (i.e., 5-5'-bi-1,2,4-triazinyl compounds)[69] as drawn here. These observations suggest that it is likely that the observed spark adduct is predominantly the 5-5'linked dimer.

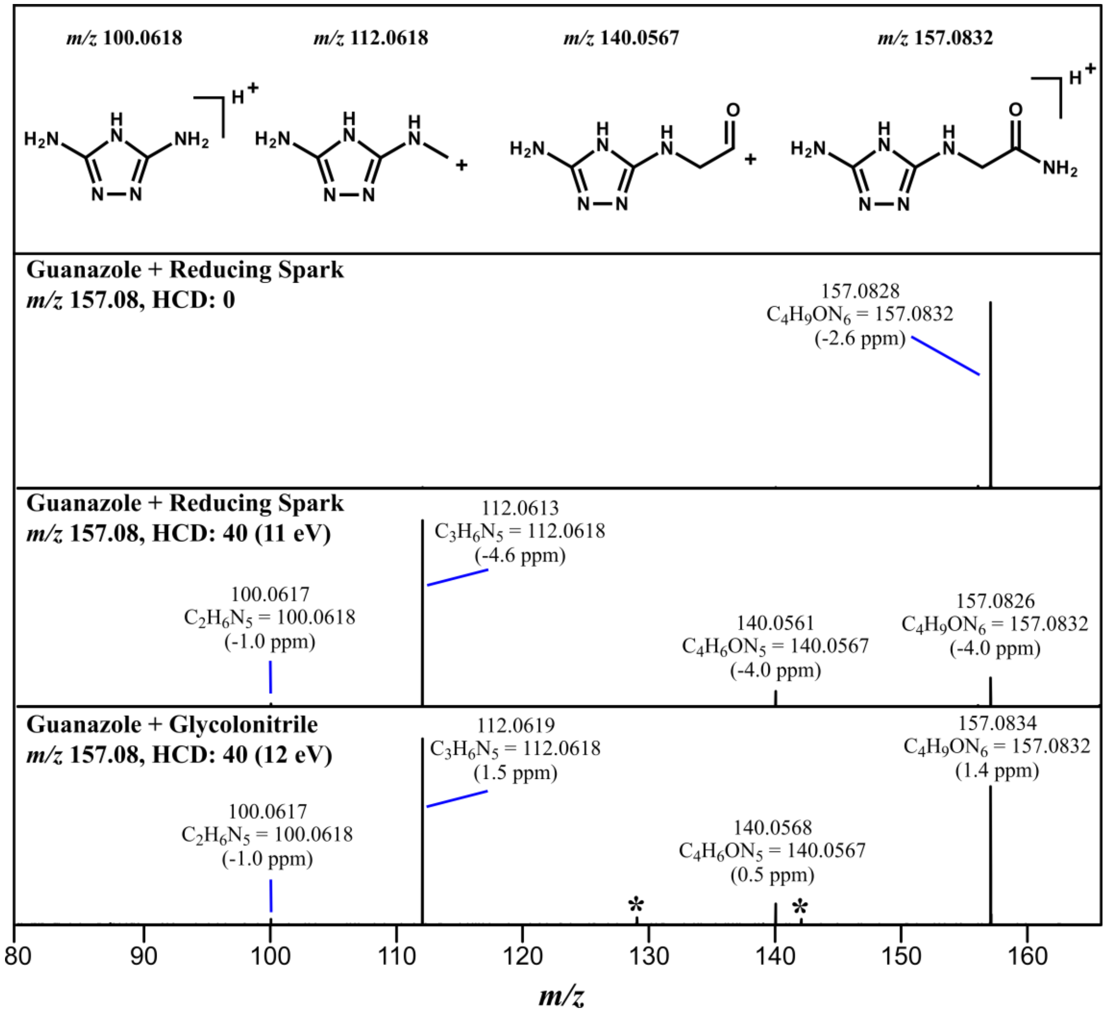

Figure S22: Product ion spectra of guanazole adduct (*m/z* 157.08) produced via incubation of guanazole with a spark mixture generated under a reducing atmosphere (second and third panels from the top) matches that from guanazole incubated with glycolonitrile (same *m/z* precursor ion, bottom panel). Fragmentation of the precursor ion produces fragments consistent with a two-carbon amide side chain (i.e., NH_3 loss and $HCONH_2$ loss) attached to guanazole (*m/z* 100.0618). Note that the fragments drawn represent only one of two possible structures (the other being addition at the ring nitrogen). The * indicates interferences in the fragmentation spectra (*m/z* 129.0700 and *m/z* 142.0777) that are consistent only with the chemical formula $C_{10}H_9$ and $C_{11}H_{10}$, respectively (within 25 ppm error): as the isolated adduct only has four carbons, these peaks cannot be a fragment coming off of the starting precursor ion ($C_4H_9ON_6$).

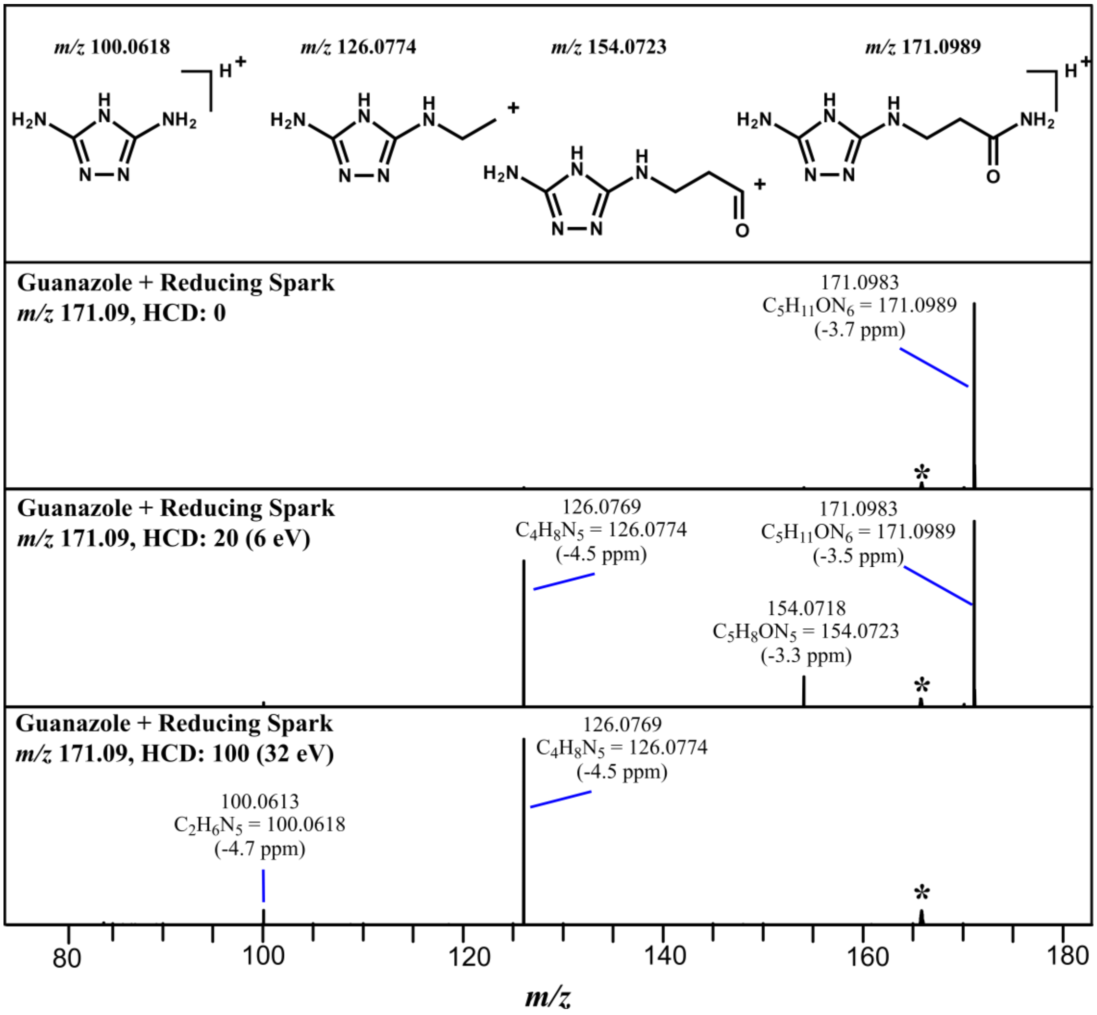

Figure S23: Product ion spectra of guanazole adduct (*m/z* 171.09) produced via incubation of guanazole with a spark mixture generated under a reducing atmosphere. Fragmentation of the precursor ion produces fragments consistent with a three-carbon amide side chain (i.e., NH_3 loss and $HCONH_2$ loss) attached to guanazole (*m/z* 100.0618). Note that the fragments drawn represent only one of two possible structures (the other being addition at the ring nitrogen). The * indicates a common instrument artifact (*m/z* 165.8).

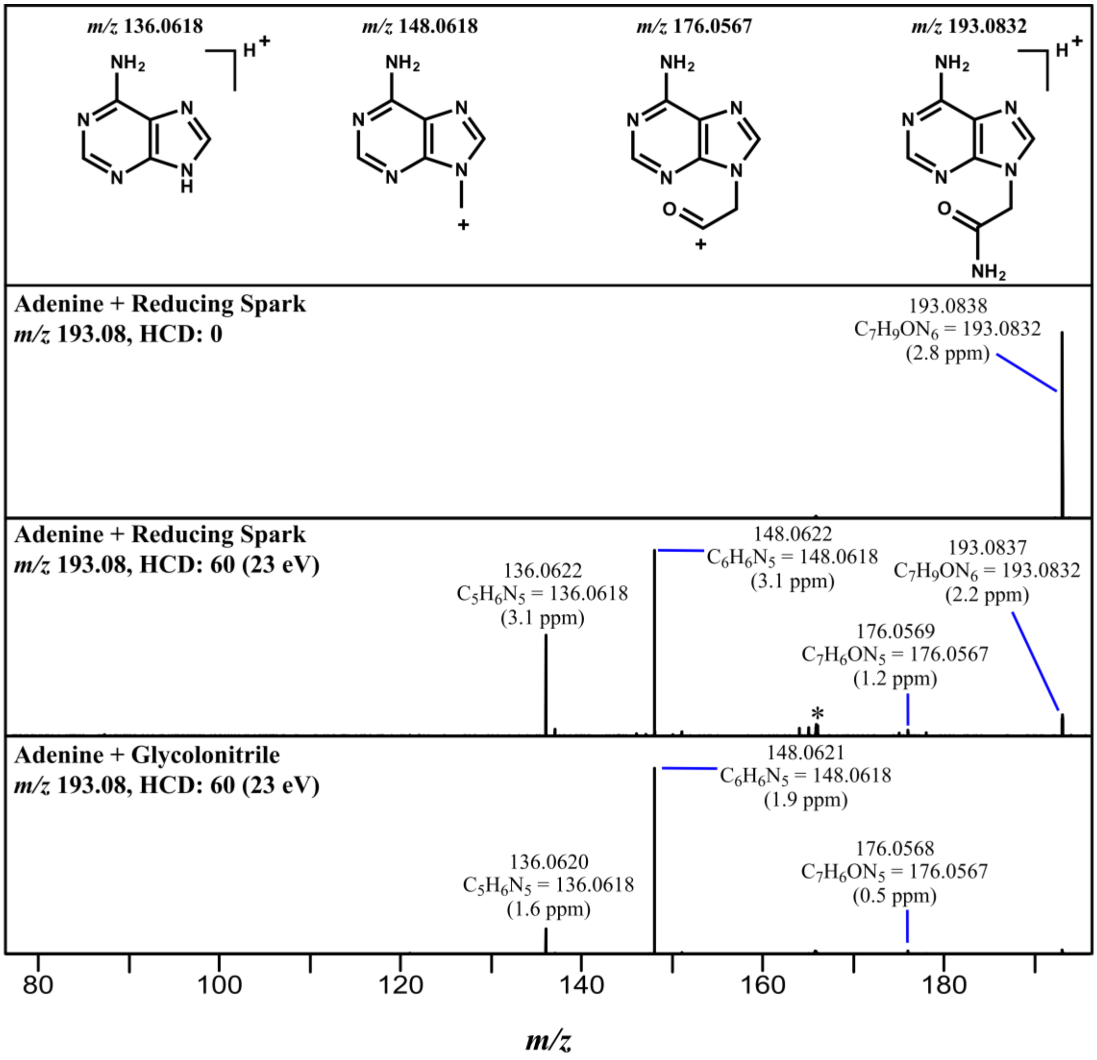

Figure S24: Product ion spectra of adenine adduct (*m/z* 193.08) produced via incubation of adenine with a spark mixture generated under a reducing atmosphere (second and third panels from the top) matches that from adenine incubated with glycolonitrile (same *m/z* precursor ion, bottom panel). Fragmentation of the precursor ion produces fragments consistent with a two-carbon side chain containing a terminal amide group (i.e., NH_3 and $HCONH_2$ loss) attached to adenine (*m/z* 136.0618). Note that the fragments drawn represent only one possible structure (the others being addition at the exocyclic amine group or the other ring nitrogens). * indicates a common instrument artifact (*m/z* 165.9).

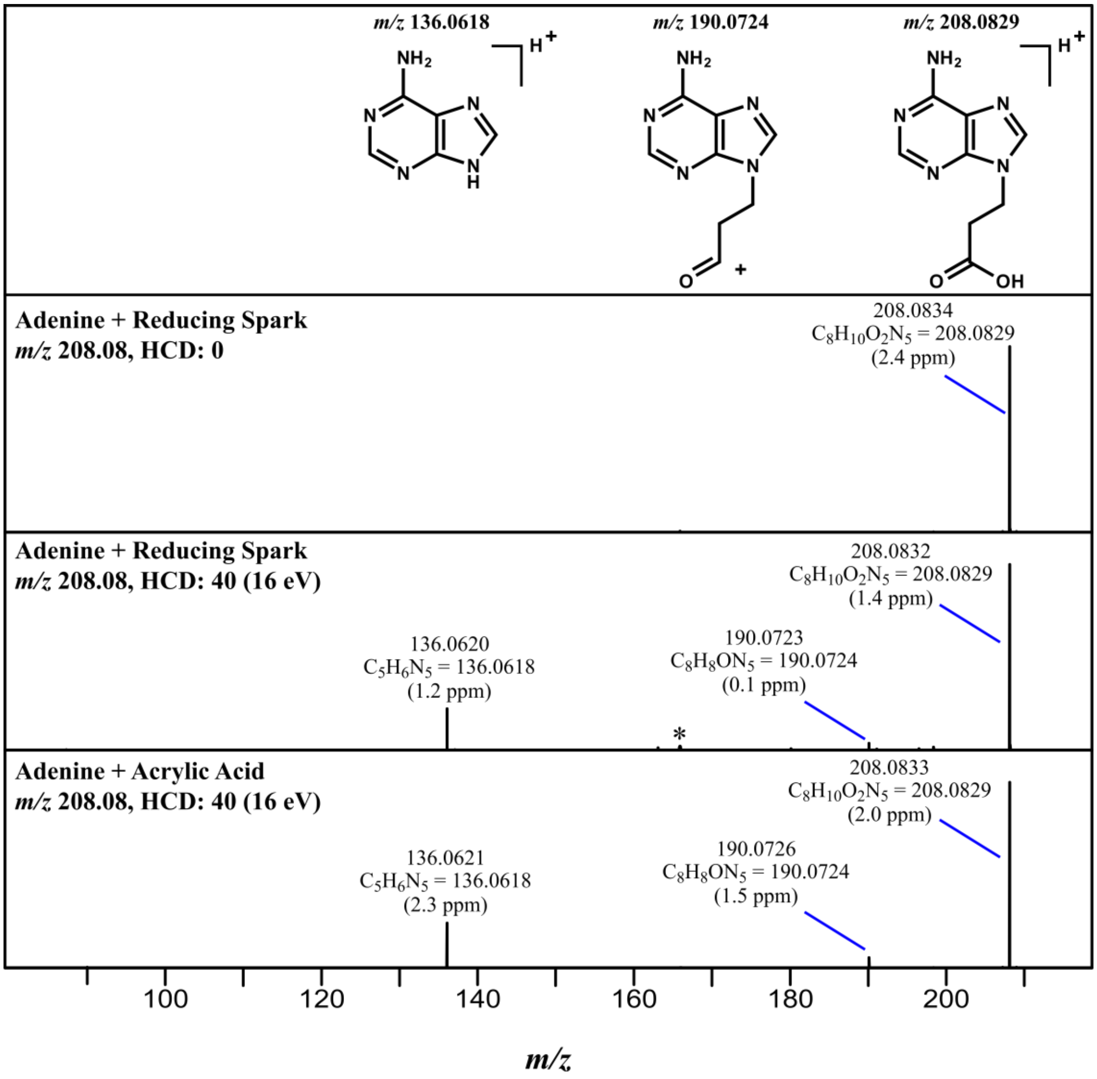

Figure S25: Product ion spectra of adenine adduct (*m/z* 208.08) produced via incubation of adenine with a spark mixture generated under a reducing atmosphere (second and third panels from the top) matches that from adenine incubated with acrylic acid (same *m/z* precursor ion, bottom panel). Fragmentation of the precursor ion produces fragments consistent with a three-carbon side chain containing a terminal OH group (i.e., H_2O loss) attached to adenine (*m/z* 136.0618). Note that the fragments drawn represent only one possible structure (the others being addition at the exocyclic amine group or the other ring nitrogens). * indicates a common instrument artifact (*m/z* 165.9).

5.0 SI References

1. Robertson, M. P. & Miller, S. L. Prebiotic synthesis of 5-substituted uracils: A bridge between the RNA world and the DNA-protein world. *Science* **268,** 702–705 (1995).

2. G. Schlesinger & Miller, S. L. Prebiotic synthesis in atmospheres containing CH_4, CO, and CO_2. II. Hydrogen cyanide, formaldehyde, and ammonia. *J. Mol. Evol.* **19,** 383–390 (1983).

3. Smith, K. E., House, C. H., Dworkin, J. P. & Callahan, M. P. Spontaneous oligomerization of nucleotide alternatives in aqueous solutions. *Orig. Life Evol. Biosph.* **47,** 3–11 (2017).

4. Kukharev, B. F., Stankevich, V. K., Klimenko, G. R. & Kukhareva, V. A. 2-Aminopyridine condensation with formaldehyde. *Russ. J. Org. Chem.* **45,** 1255–1256 (2009).

5. Schlesinger, G. & Miller, S. L. Equilibrium and kinetics of glycolonitrile formation in aqueous solution. *J. Am. Chem. Soc.* **95,** 3729–3735 (1973).

6. Johnson, S. L. & Morrison, D. K. Kinetics and mechanism of decarboxylation of N-arylcarbamates. Evidence for kinetically important zwitterionic carbamic acid species of short lifetime. *J. Am. Chem. Soc.* **94,** 1323–1334 (1972).

7. Warner, R. C. The kinetics of the hydrolysis of urea and of arginine. *J. Biol. Chem.* **142,** 705–723 (1942).

8. Cleaves, H. J. The reactions of nitrogen heterocycles with acrolein: scope and prebiotic significance. *Astrobiology* **2,** 403–15 (2002).

9. Crippa, S., Di Gennaro, P., Lucini, R., Orlandi, M. & and Rindone, B. Characterisation of adducts of nucleic bases and acrylic monomers. *Soc. Chim. Ital.* **123,** 197–203 (1993).

10. Nelson, K. E., Levy, M. & Miller, S. L. Peptide nucleic acids rather than RNA may have been the first genetic molecule. *Proc. Natl. Acad. Sci.* **97,** 3868–71 (2000).

11. Sanchez, R. A., Ferris, J. P. & Orgel, L. E. Studies in prebiotic synthesis: II. Synthesis of purine precursors and amino acids from aqueous hydrogen cyanide. *J. Mol. Biol*. **30,** 223–253 (1967).

12. Feldman, M. YA. The condensation of adenine and adenosine with formaldehyde. *Biochemistry* **27,** 321–326 (1962).

13. Feldman, M. YA. Reaction of formaldehyde with nucleotides and ribonucleic acid. *Biochim. Biophys. Acta* **149,** 20–34 (1967).

14. Johnson, F., Pillai, K. M. R., Grollman, A. P., Tseng, L. & Takeshita, L. Synthesis and

biological activity of a new class of cytotoxic agents: N-(3-oxoprop-2-enyl)-substituted pyrimidines and purines. *J.Med.Chem.* **27,** 954–958 (1984).

15. Furukawa, Y., Miyashita, O. & Honjo, M. A new reaction of nucleotides with cyanoacetylene. *Chem. Pharm. Bull.* **22,** 2552–2556 (1974).

16. Sanchez, R. A., Ferris, J. P. & Orgel, L. E. Cyanoacetylene in prebiotic synthesis. *Science* **154,** 784–785 (1966).

17. Ferris, J. P., Joshi, P. C. C., Edelson, E. H. & Lawless, J. G. HCN: a plausible source of purines, pyrimidines and amino acids on the primitive Earth. *J. Mol. Biol.* **11,** 293–311 (1978).

18. Borquez, E., Cleaves, H. J., Lazcano, A. & Miller, S. L. An investigation of prebiotic purine synthesis from the hydrolysis of HCN polymers. *Orig. Life Evol. Biosph.* **35,** 79–90 (2005).

19. Ferus, M. et al. Formation of nucleobases in a Miller–Urey reducing atmosphere. *Proc. Natl. Acad. Sci.* **114,** 4306–4311 (2017).

20. Menor-Salván, C., Ruiz-Bermejo, D. M., Guzmán, M. I., Osuna-Esteban, S. & Veintemillas-Verdaguer, S. Synthesis of pyrimidines and triazines in ice: implications for the prebiotic chemistry of nucleobases. *Chem. - A Eur. J.* **15,** 4411–4418 (2009).

21. Ruiz-Bermejo, M., Zorzano, M.-P. & Osuna-Esteban, S. Simple Organics and biomonomers identified in HCN polymers: an overview. *Life* **3,** 421–448 (2013).

22. Saladino, R. et al. One-pot TiO_2-catalyzed synthesis of nucleic bases and acyclonucleosides from formamide: implications for the origin of life. *ChemBioChem* **4,** 514–521 (2003).

23. Saladino, R. et al. Synthesis and degradation of nucleobases and nucleic acids by formamide in the presence of montmorillonites. *ChemBioChem* **5,** 1558–1566 (2004).

24. Miyakawa, S., Cleaves, H. J. & Miller, S. L. The cold origin of life: B. Implications based on pyrimidines and purines produced from frozen ammonium cyanide solutions. *Orig. Life Evol. Biosph.* **32,** 209–218 (2002).

25. Voet, A. B. & Schwartz, A. W. Uracil synthesis via HCN oligomerization. *Orig. Life* **12,** 45–49 (1982).

26. Ferris, J. P., Sanchez, R. A. & Orgel, L. E. Studies in prebiotic synthesis. III. Synthesis of pyrimidines from cyanoacetylene and cyanate. *J. Mol. Evol.* **33,** 693–704 (1968).

27. Harada, K. & Suzuki, S. The new synthesis of uracil and 1,3-dimethyluracil. *Tetrahedron Lett.* **27,** 2321–2322 (1976).

28. Oró, J. Non-enzymatic formation of purines and pyrimidines. *Fed. Proc.* **22,** 681 (1963).

29. Schlesinger, G., Miller, S. L., Diego, S. & Jolla, L. Prebiotic synthesis in atmospheres containing CH_4, CO, and CO_2. II. Hydrogen cyanide, formaldehyde, and ammonia. *J. Mol. Evol.* **19,** 383–390 (1983).

30. Cooper, G. J. T. *et al.* Miller–Urey spark-discharge experiments in the deuterium world. *Angew. Chem. Int. Ed. Engl.* **56,** 8079–8082 (2017).

31. Parker, E. T. *et al.* Conducting Miller-Urey experiments. *J. Vis. Exp.* **83,** e51039; 10.3791/51039 (2014).

32. Smith, K. E., Gerakines, P. A. & Callahan, M. P. Metabolic precursors in astrophysical ice analogs: implications for meteorites and comets. *Chem. Commun.* **51,** 11787–11790 (2015).

33. Smith, K. E., Callahan, M. P., Gerakines, P. A., Dworkin, J. P. & House, C. H. Investigation of pyridine carboxylic acids in CM2 carbonaceous chondrites: potential precursor molecules for ancient coenzymes. *Geochim. Cosmochim. Acta* **136,** 1–12 (2014).

34. Friedmann, N., Miller, S. L. & Sanchez, R. A. Primitive earth synthesis of nicotinic acid derivatives. *Science* **171,** 1026–1027 (1971).

35. Nuevo, M., Milam, S. N. & Sandford, S. A. Nucleobases and prebiotic molecules in organic residues produced from the ultraviolet photo-irradiation of pyrimidine in NH_3 and H_2O + NH_3 ices. *Astrobiology* **12,** 295–314 (2012).

36. Materese, C. K., Nuevo, M., Bera, P. P., Lee, T. J. & Sandford, S. A. Thymine and other prebiotic molecules produced from the ultraviolet photo-irradiation of pyrimidine in simple astrophysical ice analogs. *Astrobiology* **13,** 948–962 (2013).

37. Cleaves, H. J., Nelson, K. E. & Miller, S. L. The prebiotic synthesis of pyrimidines in frozen solution. *Naturwissenschaften* **93,** 228–231 (2006).

38. Gautier, T. *et al.* Development of HPLC-Orbitrap method for identification of N-bearing molecules in complex organic material relevant to planetary environments. *Icarus* **275,** 259–266 (2016).

39. Chen, M. C. *et al.* Spontaneous prebiotic formation of a β-ribofuranoside that self-assembles with a complementary heterocycle. *J. Am. Chem. Soc.* **136,** 5640–5646 (2014).

40. Saladino, R., Crestini, C., Costanzo, G., Negri, R. & Di Mauro, E. A possible prebiotic synthesis of purine, adenine, cytosine, and 4(3H)-pyrimidinone from formamide: implications for the origin of life. *Bioorganic Med. Chem.* **9,** 1249–1253 (2001).

41. Saladino, R., Botta, G., Delfino, M. & Di Mauro, E. Meteorites as catalysts for prebiotic chemistry. *Chem. Eur. J.* **19,** 16916–16922 (2013).

42. Menor-Salván, C. & Marín-Yaseli, M. R. A new route for the prebiotic synthesis of nucleobases and hydantoins in water/ice solutions involving the photochemistry of acetylene. *Chem. Eur. J.* **19,** 6488–6497 (2013).

43. Saladino, R. *et al.* Meteorite-catalyzed syntheses of nucleosides and of other prebiotic compounds from formamide under proton irradiation. *Proc. Natl. Acad. Sci.* **112,** E2746–E2755 (2015).

44. Bean, H. D. *et al.* Formation of a β-pyrimidine nucleoside by a free pyrimidine base and ribose in a plausible prebiotic reaction. *J. Am. Chem. Soc.* **129,** 9556–9557 (2007).

45. Sheng, Y., Bean, H. D., Mamajanov, I., Hud, N. V., & Leszczynski, J. Comprehensive investigation of the energetics of pyrimidine nucleoside formation in a model prebiotic reaction. *J. Am. Chem. Soc.* **131,** 16088–16095 (2009).

46. Van der Velden, W. & Schwartz, A. W. Search for purines and pyrimidines in the Murchison meteorite. *Geochim. Cosmochim. Acta* **41,** 961–968 (1977).

47. Stoks, P. G. & Schwartz, A. W. Uracil in carbonaceous meteorites. *Nature* **282,** 709–710 (1979).

48. Martins, Z. *et al.* Extraterrestrial nucleobases in the Murchison meteorite. *Earth Planet. Sci. Lett.* **270,** 130–136 (2008).

49. Mungi, C. V., Singh, S. K., Chugh, J. & Rajamani, S. Synthesis of barbituric acid containing nucleotides and their implications for the origin of primitive informational polymers. *Phys. Chem. Chem. Phys.* **18,** 20144–20152 (2016).

50. Cafferty, B. J. *et al.* Efficient self-assembly in water of long noncovalent polymers by nucleobase analogues. *J. Am. Chem. Soc.* **135,** 2447–2450 (2013).

51. Choughuley, A. S. U., Subbaraman, A. S., Kazi, Z. A. & Chadha, M. S. A possible prebiotic synthesis of thymine: uracil-formaldehyde-formic acid reaction. *Biosystems* **9,** 73–80 (1977).

52. Kolb, V. M., Dworkin, J. P. & Miller, S. L. Alternative bases in the RNA world: the prebiotic synthesis of urazole and its ribosides. *J. Mol. Evol.* **38,** 549–557 (1994).

53. Hayatsu, R., Studier, M. H., Oda, A., Fuse, K. & Anders, E. Origin of organic matter in early solar system-II. Nitrogen compounds. *Geochim. Cosmochim. Acta* **32,** 175–190 (1968).

54. Li, C., Cafferty, B. J., Karunakaran, S. C., Schuster, G. B. & Hud, N. V. Formation of supramolecular assemblies and liquid crystals by purine nucleobases and cyanuric acid in water: implications for the possible origins of RNA. *Phys. Chem. Chem. Phys.* **18,** 20091–20096 (2016).

55. Chan, Q. H. S. *et al.* Organic matter in extraterrestrial water-bearing salt crystals. *Sci. Adv.* **4,** eaao3521; 10.1126/sciadv.aao3521 (2018).

56. Saladino, R. *et al.* Synthesis and degradation of nucleic acid components by formamide and iron sulfur minerals. *J. Am. Chem. Soc.* **130,** 15512–15518 (2008).

57. Materese, C. K., Nuevo, M. & Sandford, S. A. The formation of nucleobases from the ultraviolet photoirradiation of purine in simple astrophysical ice analogues. *Astrobiology* **17,** 761-770 (2017).

58. Barks, H. L. *et al.* Guanine, adenine, and hypoxanthine production in UV-irradiated formamide solutions: relaxation of the requirements for prebiotic purine nucleobase formation. *ChemBioChem* **11,** 1240–1243 (2010).

59. Callahan, M. P. *et al.* Carbonaceous meteorites contain a wide range of extraterrestrial nucleobases. *Proc. Natl. Acad. Sci.* **108,** 13995–13998 (2011).

60. Stoks, P. G. & Schwartz, A. W. Nitrogen-heterocyclic compounds in meteorites: significance and mechanisms of formation. *Geochim. Cosmochim. Acta* **45,** 563–569 (1981).

61. Fuller, W. D., Sanchez, R. A. & Orgel, L. E. Studies in prebiotic synthesis. VII. Solid-state synthesis of purine nucleosides. *J. Mol. Evol.* **1,** 249–257 (1972).

62. Fuller, W. D., Sanchez, R. A. & Orgel, L. E. Studies in prebiotic synthesis. VI. Synthesis of purine nucleosides. *J. Mol. Biol.* **67,** 25–33 (1972).

63. Bera, P. P., Stein, T., Head-Gordon, M. & Lee, T. J. Mechanisms of the formation of adenine, guanine, and their analogues in UV-irradiated mixed NH_3:H_2O molecular ices containing purine. *Astrobiology* **17,** 771-785 (2017).

64. Šponer, J. E. *et al.* Emergence of the first catalytic oligonucleotides in a formamide-based origin scenario. *Chem. Eur. J.* **22,** 3572–3586 (2016).

65. Marín-Yaseli, M. R., Mompeán, C. & Ruiz-Bermejo, M. A prebiotic synthesis of pterins. *Chem. Eur. J.* **21,** 13531–13534 (2015).

66. Heinz, B., Ried, W. & Dose, K. Thermal generation of pteridines and flavines from amino acid mixtures. *Angew. Chem. Int. Ed. Engl.* **18,** 478–483 (1979).

67. Becker, S. et al. A high-yielding, strictly regioselective prebiotic purine nucleoside formation pathway. *Science* **352,** 833–836 (2016).

68. Mola, L. et al. Nucleophile-catalyzed additions to activated triple bonds. protection of lactams, imides, and nucleosides with MocVinyl and related groups. *J. Org. Chem.* **78,** 5832-5842 (2013).

69. Krass, D. K., Chen, T. K. & Paudler, W. W. 1,2,4-Triazines X: Dimerizations of 1,2,4-triaznes. *J. Heterocycl. Chem.* **10,** 343–345 (1973).
